# Faster Speech Slows Predictive Processing: Evidence from Mandarin

**DOI:** 10.1177/17470218261446782

**Published:** 2026-04-20

**Authors:** Huanhuan Yin, Patrick Sturt, Martin J. Pickering

**Affiliations:** 1 Department of Psychology, University of Edinburgh, UK

**Keywords:** speech rate, prediction, prediction mechanism, language comprehension, cognitive resources, contextual predictability

## Abstract

During language comprehension, people often predict what they are likely to encounter, but such predictions are affected by processing difficulty. In this study, we explored how speech rate influences listeners’ prediction speed using three different methods. Participants read sentence contexts that either were or were not highly predictive of a final word, and we measured how quickly participants judged whether a given letter was contained in that final word (Experiment 1), named a picture corresponding to that final word (Experiment 2), and named that final word (Experiment 3). We manipulated the speech rates and contextual predictability of the sentences. Across the experiments, participants responded more slowly at a faster speech rate, and this slowing was greater in more predictable than less predictable contexts. These results suggest that a faster speech rate slows down prediction speed, reinforcing the idea that prediction demands cognitive resources.

## Introduction

During language comprehension, people often try to predict what a speaker is going to say next. But making such predictions may not always be straightforward and may take up time and resources. In this paper, we consider the effect of speech rate on prediction and ask whether it affects prediction similarly for more or less predictable words. We addressed these questions by examining how speech rate influences listeners’ prediction speed in three experiments using Mandarin Chinese.

Prediction occurs when comprehenders pre-activate linguistic representations associated with predictable words (Altmann & Kamide, 1999; Federmeier, 2007; Wicha et al., 2003). Such predictions occur at multiple levels (for reviews, see Huettig, 2015; Kuperberg & Jaeger, 2016; Pickering & Gambi, 2018; Ryskin & Nieuwland, 2023), including semantics (Altmann & Kamide, 1999; Grisoni et al., 2017; Huang et al., 2023; Shao et al., 2022), syntax (Arai & Keller, 2013; Van Berkum et al., 2005; Wicha et al., 2004), and form (DeLong et al., 2005; Dikker et al., 2010; Ito et al., 2020; Kukona, 2020; Shen et al., 2021; Zhao et al., 2024).

One question, however, is whether prediction necessarily requires substantial cognitive resources. Some researchers have argued that at least part of prediction may be automatic and cost-free, such as pre-activation driven by associative priming (Heilbron et al., 2022; Huettig, 2015; Kuperberg & Jaeger, 2016). For example, Kukona et al. (2011) found that listeners initially predictively fixated not only contextually appropriate referents but also referents associated with the verb, even when they were not semantically compatible with the context (see also Corps et al., 2022; Kukona et al., 2014). These findings suggest that at least initial predictions may involve rapid automatic processing. But of course, just because predictions are rapid does not demonstrate that they are cost-free.

At the same time, many researchers argue that, despite its ubiquity, prediction is cognitively demanding and resource-limited (Dell & Chang, 2014; F. Chang et al., 2006; Federmeier, 2007; Huettig & Mani, 2016; Kuperberg & Jaeger, 2016; Pickering & Gambi, 2018). One influential proposal is the production-based account (Pickering & Gambi, 2018), according to which comprehenders covertly imitate the speaker to derive the speaker’s intention and run that intention through their production system, activating representations of the upcoming word as in actual production: from meaning, to syntax, and to sound, but without articulation. Importantly, the extent to which an upcoming word is pre-activated depends on time and resources. Consistent with a resource-dependent view, predictive effects are reduced under concurrent memory load (Allison et al., 2025; Ito et al., 2018; Liu et al., 2025), and are weaker in populations with lower domain-general executive resources (Kang et al., 2020), such as children (Gambi et al., 2018; Mani & Huettig, 2012) and older people (Dave et al., 2018; Federmeier et al., 2010; Salthouse, 1996; Schuckart et al., 2026; Wlotko et al., 2012).

On cognitively-demanding accounts, any factor that taxes resources should impair prediction. One likely factor is fast speech rate, which can tax processing resources for non-predictive aspects of comprehension (Müller et al., 2019; Simantiraki & Cooke, 2020); at the same time, faster speech induces entrainment: listeners’ speaking rate during production (and also their temporal processing in perception) converge toward the speaker’s rate. Such entrainment will lead listeners to expect the utterance to finish sooner, thereby affecting their response latencies. These two consequences of faster speech—greater resource demands and entrainment—may therefore interact in tasks that use response latency to assess prediction. Below, we review evidence for effects of fast speech on non-predictive aspects of comprehension, evidence for speech rate entrainment, and prior work on speech rate effects on prediction, and we explain how earlier findings may have been confounded by non-predictive comprehension costs or entrainment.

### Effects of Fast Speech Rate on Non-Predictive Aspects of Comprehension

Speech rate refers to the speed of articulation (A. C.-S. Chang, 2018; Iwarsson et al., 2021) and is typically measured as number of syllables or words uttered per unit of time. Although there is no consensus on what counts as fast or slow, typical British English speaking rate ranges from 3.16 to 5.33 syllables per second (Tauroza & Allison, 1990), and average Mandarin speaking rate is about 3.8 syllables per second (Lee & Chan, 2003; Yuan et al., 2006).

Fast speech taxes cognitive resources for non-predictive aspects of comprehension, likely because it degrades speech perception, for example, by blurring acoustic cues (Janse, 2003) or impairing word recognition (Koch & Janse, 2016). In a combined electroencephalography (EEG) and eye-tracking study using sentence-picture matching, Müller et al. (2019) found that fast speech elicited larger peak pupil dilations (indicating greater listening effort) but an earlier P2 peak (indexing neural speech tracking of the input), suggesting faster but more effortful processing. Simantiraki and Cooke (2020) found that listeners preferred faster speech in quiet environments but increasingly opted for slower speech as noise levels rose, suggesting that slower speech may help compensate for the reduced cognitive resources available in noisy conditions. Similarly, Winn and Teece (2021) had participants (who were cochlear-implant users) listen to sentences presented at fast and slow rates and found greater recovery of pupil size back to baseline after slow than after fast sentences, suggesting easier processing at the slow rate, even though participants found slow and fast sentences equally intelligible.

Together, these findings suggest that fast speech increases listeners’ listening effort (though it does not necessarily affect intelligibility). Because fast speech increases the effort required for perceptual decoding and for integrating the input (e.g., lexical access and syntactic processing), it can consume capacity that would otherwise support predictive processing, thereby leaving fewer resources available for prediction. Under resource-dependent accounts of prediction (e.g., Pickering & Gambi, 2018), faster speech should therefore slow predictive processing.

### Speech Rate Entrainment

During both comprehension and production, listeners entrain to a speaker’s rate. In production, for example, Jungers and Hupp (2009) found that participants described pictures more quickly after hearing a fast prime sentence than after a slow one. Schultz et al. (2016) found that participants’ speech rates converged with their partners over the course of a dialogue, and corpus analyses likewise show that speakers converge to their interlocutor’s rate (Cohen Priva et al., 2017).

In comprehension, listeners’ speech perception is affected by the speech rate of the preceding context. Dilley and Pitt (2010) manipulated the rate of the sentence context (e.g., *Deena doesn’t have any. . .*) and found that a speeded context caused listeners to fail to perceive a function word that had been uttered (i.e., hearing *leisure or time* as *leisure time*), whereas a slowed context caused listeners to erroneously perceive a function word that had not been uttered (hearing *leisure time* as *leisure or time*).

Some theories propose that listeners use such entrained timing to predict when the speaker will finish and to time the initiation of articulation (Garrod & Pickering, 2015; Wilson & Wilson, 2005). If so, speech rate during comprehension should influence when listeners initiate their responses. In accord with this, Corps et al. (2022) presented participants with questions in which the context (e.g., *Do you have a. . .*) was fast or slow relative to the final word (e.g., *dog?*). Participants responded more quickly after fast contexts, suggesting that listeners entrained to the fast speech rate, which led to earlier response initiation.

However, entrainment effects on response timing appear to be influenced by cues that predict turn endings. Torreira and Bögels (2022) had Dutch participants say *ja* (“yes”) after speech-like stimuli [ma] that varied in prosody and rates. Participants responded more quickly to fast stimuli, but only when stimuli lacked prosodic cues to the end of the turn; with clear cues, faster rate produced longer latencies. In a spontaneous conversational corpus, where turn-ending cues are highly likely to be available, Hoogland et al. (2023) found that higher speech rate was also associated with longer response latencies. Together, these findings suggest that the timing of response initiation reflects both entrainment and prediction, and that rate-driven entrainment can sometimes be overshadowed by turn-ending cues.

In sum, faster speech increases listening effort and thereby reduces resources available for prediction. But at the same time, it causes listeners to entrain to the fast rate, and so they expect the utterance to finish sooner, and thereby initiate their own responses earlier (in the absence of prediction).

### The Effect of Fast Speech Rate on Prediction

Some studies suggest that faster rate interferes with prediction. Wlotko and Federmeier (2015) had participants read two-sentence passages (e.g., *They wanted to make the hotel look more like a tropical resort. So along the driveway they planted rows of . . .*) in which the final word was predictable (*palms*), categorically related to the predictable word (*pines*), or unrelated (*tulips*). Words were presented at 500 or 250 ms stimulus-onset asynchronies (SOAs) in separate blocks. At 500 ms SOA, categorically related words elicited reduced N400 amplitudes compared to unrelated words. This finding provides some evidence for prediction (and replicates Federmeier & Kutas, 1999), but the effect occurred after the final word was encountered and may therefore be due to ease of integration rather than prediction (see Pickering & Gambi, 2018). At 250 ms, this effect of semantic similarity diminished; if the effect is due to prediction, then speeded written language interfered with it.^1^

In another ERP study, Ito et al. (2016) had participants read high-cloze sentence contexts (e.g., *Living alone is too expensive, so the students will share a . . .*), followed by a predictable word (*flat*), a word semantically related to the predictable word (*wall*), a word phonologically related to the predictable word (*flag*), or an unrelated word (*bell*), at a rate of 500 or 700 ms SOA. They found an N400 reduction for semantically related words at both SOAs, but an N400 reduction for form-related words only at 700 ms and only in very high-cloze sentences. In addition, they found a post-N400 enhanced positivity for form-related words in comparatively lower cloze sentences at the 700 ms but not the 500 ms SOA, suggesting that listeners could detect the form conflict between form-related words and predictable words in such contexts only at a slower presentation rate. These results suggested that comprehenders predicted less at the faster rate. But note that both Ito et al. (2016) and Wlotko and Federmeier (2015) manipulated presentation rate in reading, not acoustic speech rate during listening.

Eye-tracking studies show that fast speech rate impairs prediction. Huettig and Guerra (2019) had Dutch speakers view a target object (e.g., a bicycle) and three unrelated distractors for a short or a long time and then listen to Dutch sentences presented at a slow or normal rate (e.g., *Look at the displayed bicycle*). The determiner was compatible in gender with the target object but none of the distractors, thus allowing prediction. Participants looked predictively at the target object in both speech-rate conditions when they had a long preview (Experiment 1), but only at the slow rate condition when they had a short preview (Experiment 2). Thus, the slower rate appeared to enhance prediction. But the target picture had a different gender from the other three pictures, and so participants might have worked out that it would be the target before hearing the determiner.

However, there is also evidence that faster rate does not always constrain prediction. Fernandez et al. (2020) had participants listen to short stories (e.g., *One day a wolf and a deer were sleeping near a cave. The wolf became crazed and the wolf attacked the deer. A hawk watched as the deer escaped*) followed by a comprehension probe (e.g., *Point to who the wolf was attacking near the cave*) while viewing four objects (wolf, deer, hawk, cave). Speech rate was varied (3.5, 4.5, 5.5, 6.0 syllables per second). Both young and older adults made more anticipatory looks to the target picture after the verb at 4.5 than 3.5 syllables per second (Experiment 1), but the anticipatory looks decreased at the fastest rate (6.0). These findings indicate an upper bound on rate adaptation: Listeners can accommodate moderate increases, but prediction deteriorates when speech is too fast, likely because listening imposes excessive cognitive load.

Similarly, Kukona (2023) had participants listen to predictive sentences (e.g., *What the man will ride, which is shown on this page, is the bike*) while viewing visual arrays containing two objects (e.g., *a bike* and *a kite*). Participants moved their mouse cursor to the predictable referent (here, the bike) before hearing it. Importantly, this prediction effect occurred not only at a natural speech rate (~3 syllables/second), but also at rates twice (~6) or three times (~9) as fast as the natural rate, with predictions occurring even earlier at faster rates. However, prediction effect decreased as rate increased. These findings suggest that participants partially adapted their prediction to higher rates: Faster speech advanced the timing of prediction but constrained its strength.

In summary, the current evidence regarding the effect of speech rate on comprehenders’ prediction is mixed, with some studies potentially measuring integration rather than prediction (e.g., Wlotko & Federmeier, 2015). Another limitation of previous research is the failure to separate fast speech rate effects on prediction from effects on bottom-up comprehension processes. It is plausible that a faster speech rate alters aspects of comprehension other than prediction or a combination of rate-based entrainment and prediction. Finally, it remains unclear whether faster speech rate affects prediction equally across contexts with different levels of predictability.

### The Present Study

We therefore conducted a thorough examination of how speech rate affects prediction speed using three different paradigms, each serving a specific purpose. Experiment 1 used a letter-judgment task to test whether fast speech slows prediction in highly predictable contexts. Experiment 2 used picture naming to separate rate effects on prediction from effects on other aspects of comprehension by comparing predictable versus unpredictable fragments. Experiment 3 used word naming to assess whether rate effects on prediction vary with contextual predictability.

In all experiments, our primary dependent measure was response latency (time to make the judgment or initiate naming). Assuming that prediction is cognitively demanding, we hypothesized that faster speech would slow prediction and thus lengthen latencies. It is of course possible that effects of rate on latency could also reflect other comprehension processes such as word recognition; Experiment 2 was designed to separate effects of rate on latency during prediction from effects on latency caused by other processes during comprehension. Experiments 2 and 3 also manipulated contextual predictability: whether the sentence context made the target word more or less predictable.

Following evidence of speech rate entrainment in production (Corps et al., 2022; Jungers & Hupp, 2009) and perception (Dilley & Pitt, 2010), we expected shorter response durations in the fast-rate than the slow-rate conditions, and shorter response latencies in the fast-rate than the slow-rate condition for unpredictable sentences (in Experiments 2 and 3). In addition, we anticipated shorter response latencies in more predictable contexts than less predictable ones. Such effects would demonstrate the efficacy of our manipulations. Finally, motivated by evidence that task goals can enhance predictive processing (Brothers et al., 2017; see also Huettig & Guerra, 2019, Exp. 3), we instructed participants in all experiments to actively predict the final word while listening, to enhance lexical prediction.

## Experiment 1

In Experiment 1, we had participants listen to high-cloze sentences truncated before the final word, presented at a fast or slow speech rate, and participants were told to actively predict what they thought the final word would be. They then were asked to judge, after the offset of the recording, whether a given letter was contained in the Pinyin of the predictable word (and then typed the word they had predicted). Pinyin is a phonetic system that represents Chinese characters using the Latin alphabet based on their pronunciation (it uses 25 of the 26 letters of the Latin alphabet, and some are pronounced the same as English). As it is an integral part of learning the pronunciation of characters and the most commonly used method for typing Chinese, Pinyin is familiar to Mandarin Chinese speakers such as our participants.

On half of the trials, the letter was included in the word (positive trials); on the other half, it was not (negative trials). We expected that participants would predict the high-cloze final word regardless of speech rate. The key dependent measure was the time participants took to make a response, because we hypothesized that the response latency would reflect the time needed to pre-activate the predictable word. To make a judgment about whether a letter is contained in a predictable word, we assumed that comprehenders would first have to pre-activate this word. Assuming that prediction takes resources and a faster speech rate taxes the comprehension system and leaves fewer cognitive resources for prediction, we expected comprehenders to take more time to pre-activate a word at a fast than slow speech rate. Thus, they should take longer to respond in the fast than the slow condition (again, on both positive and negative trials).

### Data Availability

The code and data associated with this paper are available from https://osf.io/wtbfg/files/osfstorage.

### Method

#### Participants

One hundred and twenty Chinese undergraduates (54 males and 66 females) aged 18 to 28 (*M* = 20.82 years, *SD* = 1.97) who were recruited online took part in the experiment. All were native Mandarin Chinese speakers and were given an informed consent. Each participant was paid ¥15 (about U.S.$2) for participation. The experiment was conducted online via Testable and was approved by the Ethics Committee of the Department of Psychology, University of Edinburgh.

#### Design

We used a 2 (Speech Rate: fast vs. slow) × 2 (Trial Type: positive vs. negative) within-participants and within-item design. On positive trials, the letter to be judged occupied the final position of the Pinyin of the predictable word. The reason why we asked participants to judge the last letter was that it would maximize the effect of speech rate on prediction speed because we reasoned that comprehenders would be likely to pre-activate all the letters before making a judgment. On negative trials, the letter to be judged did not occur in the Pinyin of the predictable word. Speech rate was blocked and was counterbalanced between participants (i.e., half the participants encountered fast speech first, and half encountered slow speech first).

#### Materials

We constructed 120 items (from a candidate set of 140 items) in Mandarin Chinese that consisted of a highly constraining sentence context and the highly predictable word at the end of the sentence. To do this, a separate group of 37 undergraduates were recruited via online social platforms to perform a cloze probability test. All were Mandarin Chinese native speakers and were paid ¥12 (about £1.30) for their participation. They were presented with 140 sentences truncated before the final word and were asked to complete the sentence with the first noun that came to mind. The cloze probability of a word was defined as the percentage of participants who used the word to complete the sentence. We excluded candidate items if the predictable word had a cloze probability of <70%; in total, 20 candidate items were removed on this basis. Selected items had a mean cloze value of 90.00% (range 75.68%–100%); see Table 1 for example stimuli, and the Supplemental Material A for the complete set. In all selected items, the sentence-final target was a single-character noun. We indexed character frequency with logCHR (log_10_ of CHRCount) from the SUBTLEX-CH-CHR character file (Cai & Brysbaert, 2010). On the log_10_ scale, values ranged from 1.98 to 5.51 (*M* = 3.98, *SD* = 0.59). Following van Heuven et al. (2014), we treat values of 3 or below as indicating relatively low frequency.

**Table 1.** Example Sentence Contexts from Experiment 1.

Sentence context	Target word
艾米莉今年拿到了驾照，打算明年给自己买一台_ Ai Mili Jin Nian Na Dao Le Jia Zhao, Da Suan Ming Nian Gei Zi Ji Mai Yi Tai_ (Emily got her license this year, so next year she is going to buy herself a_)	车 Che (Car)
夏天天气变化非常快，我们出门最好带一把_ Xia Tian Tian Qi Bian Hua Fei Chang Kuai, Wo Men Chu Men Zui Hao Dai Yi Ba_ (The weather changes very quickly in summer, so when we go out we’d better take an_)	伞 San (Umbrella)

We converted the sentence fragments in all three experiments to speech using Voice Maker (a commercial online text to speech converter). The same fast and slow rates were consistently used across all three of our experiments. The fast version was 50% faster than Voice Maker’s normal rate, and the slow version was 50% slower than it. The mean speech rates were 6.36 (*SD* = 0.67) and 2.42 (*SD* = 0.29) syllables per second in the fast and slow version, respectively. This indicates that our slow rate falls below and our fast rate exceeds the bounds of typical natural speech. Both the fast and the slow versions were comprehensible, as judged by two native Mandarin Chinese speakers.

To assess naturalness prior to the main studies, four native Mandarin speakers from the same pool rated a subset of the audio on a 7-point Likert scale (1 = *very unnatural*, 7 = *very natural*). We sampled 48 base sentences in total (16 per experiment). For Experiment 1, each of the 16 sentences was presented in either the fast or slow version. For Experiments 2 and 3, each of the 16 sentences was presented in one condition from the 2 × 2 design: Cloze (high vs. low) × Speech Rate (fast vs. slow). We created four counterbalanced rating lists such that (a) each rater judged 48 items, (b) each sentence appeared once per list in only one version, and (c) across lists, versions were fully counterbalanced (i.e., each sentence occurred equally often in each condition. Raters judged how natural the audio sounded, and we averaged ratings across the four raters. Mean naturalness was 4.72 (*SD* = 1.09) for fast speech and 4.01 (*SD* = 0.72) for slow speech, suggesting that both the fast and slow speech sounded broadly natural, though slow speech was perceived as less natural than fast speech (*t* = −2.66, *p* = .011).

The four conditions in this experiment were: (a) Positive trial, Fast speech rate; (b) Positive trial, Slow speech rate; (c) Negative trial, Fast speech rate; (d) Negative trial, Slow speech rate. The positive and negative trials used the same letters for judgment. On the positive trials, the proportions of individual letters were *a* (18.33%), *n* (6.67%), *e* (13.33%), *i* (12.50%), *g* (15.83%), *u* (15.00%), *o* (18.33%); on negative trials, the proportions were *a* (8.46%), *n* (22.31%), *e* (8.46%), *i* (17.69%), *g* (11.54%), *u* (14.62%), *o* (15.38%). The 120 items were first divided into 4 counterbalanced lists, such that each list contained 30 items from each condition and 1 version of each item. Each list was divided into two further lists, one in which the fast-rate speech block came first, and one in which the slow-rate speech block came first. The order of the sentences within each block was randomized for each participant.

#### Procedure

**Figure 1.**
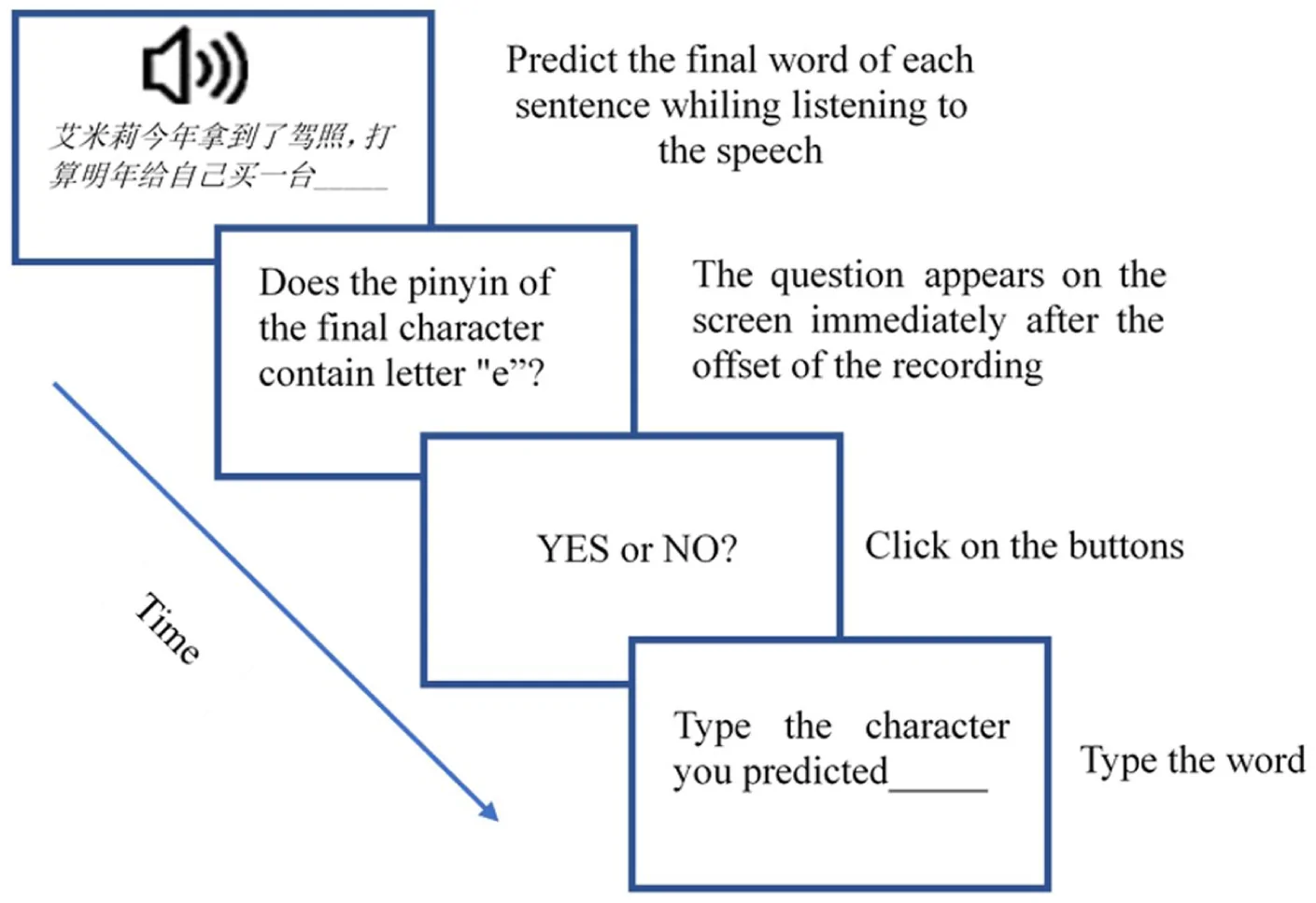
An example of a trial in Experiment 1.

Participants were randomly assigned to one of the eight lists. They sat in front of a computer screen, and the stimuli were presented in two blocks of 60 sentences, with a 5-min break between the blocks. An overview of the experimental paradigm is shown in Figure 1.

Participants were informed that they would hear sentences that were missing the final word and were instructed to actively predict the final word of each sentence while they were listening. Immediately after the offset of the penultimate word, they were asked to judge whether a letter was contained in the Pinyin of the predictable word. The participants responded by clicking a “YES” or “NO” button on the screen, and the time to respond was recorded. Each question remained on the screen until participants pressed a button. After the button press, they were instructed to type the word they had predicted. They then clicked on “Next” to begin the next trial. No feedback was given during the experiment. The experiment began with two practice trials that were used for familiarizing the participants with the procedure, and it took approximately 30 min.

#### Data Analysis

Trials in which participants did not type a word that was consistent with the word from the cloze norm test, or where response latencies were greater than 3,000 ms (18.43%), were excluded before analyzing the effects of speech rate and trial type on letter judgment accuracy. Furthermore, trials in which participants did not make a correct judgment (3.45%) and trials in which response latencies deviating more than 2 standard deviations from a participant’s conditional mean (2.78%) were excluded before analyzing the effects of speech rate and trial type on response latency.

We used the lme4 package (version 1.1.26; Bates et al., 2015) in the statistical software R (version 4.0.5; R Core Team, 2021) to analyze the effects of speech rate and trial type (positive or negative) on letter judgment accuracy and response latency. The effects of speech rate and trial type on judgment accuracy were analyzed in a generalized linear mixed effects model, using a binomial link function, and the effects of speech rate and trial type on response latency were analyzed in a linear mixed effects model. Because response latencies were right-skewed, we analyzed log-transformed latencies. The effects of speech rate and trial type in both models used sum contrast coding (Speech Rate: slow was set to −1, fast to 1; Trial Type: negative was set to −1, Positive to 1). In both models, as fixed effects, we entered speech rate and trial type (with interaction term) into the model. As random effects, we specified the maximal random effects structure in both models, for both participants and items, and if the maximal model did not converge, complexity was removed in the order of removing random correlations, interactions of random effects, and random slopes corresponding to main effects until models converged as suggested in Barr et al. (2013). The final converged model for response latency was lmer (log [RT] ~ SpeechRate * TrialType + [SpeechRate + TrialType | participant] + [SpeechRate | item], control = lmerControl [optimizer = “bobyqa”]); The final converged model for Judgment Accuracy was glmer (JudgementAccuracy ~ SpeechRate * TrialType + [SpeechRate + TrialType | participant] + [SpeechRate | item], family = “binomial,” control = glmerControl [“bobyqa”]). Significance was calculated using the lmerTest package (Kuznetsova et al., 2017). To control the family-wise error rate within the experiment, we applied Holm-Bonferroni step-down correction to the set of planned fixed-effect tests aggregated over the two models; effects were considered significant if their adjusted *p*-value was <.05.

### Results

We analyzed the effects of speech rate and trial type on comprehenders’ response latency and letter judgment accuracy.

#### Response Latency

**Figure 2.**
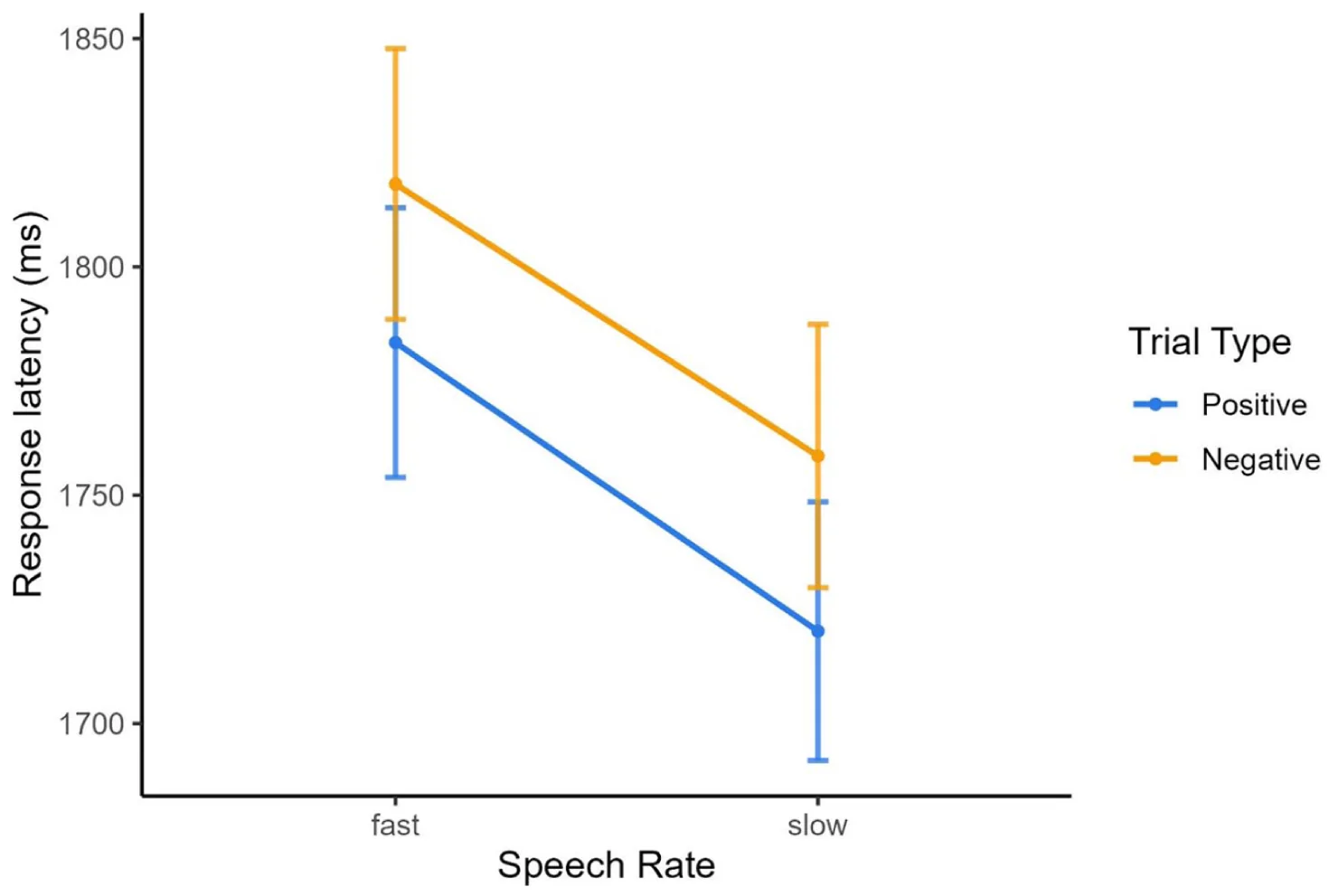
Mean response latencies (ms) in the four conditions in Experiment 1. *Note*. Error bars represent the standard error of the mean, aggregated by participants.

Table 2 lists the means and standard deviations of response latency and judgment accuracy aggregated over participants in Experiment 1 (also see Figure 2). There was an effect of speech rate^2^ (*B* = 0.02, SE = 0.01, *t* = 3.78, *p* = .002): Participants took longer to make a response when the speech rate was fast than slow. There was no effect of trial type on response latency after applying the multiple-comparisons correction (*B* = −0.01, SE = 0.00, *t* = −2.35, *p* = .083), nor was there an interaction between speech rate and trial type (*B* = 0.00, SE = 0.00, *t* = 0.21, *p* = .831).

**Table 2.** Means (and Standard Deviations) Aggregated Over Participants for Response Latency and Judgment Accuracy in Experiment 1.

Condition	Response latency (milliseconds), mean (*SD*)	Judgment accuracy (%), mean (*SD*)
Positive: fast	1,783 (323)	89.8 (13.9)
Negative: fast	1,818 (325)	94.1 (11.2)
Positive: slow	1,720 (310)	90.9 (12.7)
Negative: slow	1,759 (316)	94.8 (11.5)

#### Judgment Accuracy

There was an effect of speech rate on response accuracy (*B* = −0.17, SE = 0.06, *z* = −3.07, *p* = .01): Participants were more accurate when the speech rate was slow than fast. There was also an effect of trial type (*B* = −0.47, SE = 0.05, *z* = −9.52, *p* < .001): Participants were more accurate in the positive than the negative conditions (i.e., participants were more likely to respond YES than NO). There was no interaction between speech rate and trial type (*B* = 0.01, SE = 0.04, *z* = 0.33, *p* = .74), suggesting that fast speech rate reduced comprehenders’ prediction accuracy regardless of trial type.

### Discussion

The results of Experiment 1 showed that participants responded slower and less accurately at a faster speech rate. We interpreted this as evidence that faster speech rate slowed down comprehenders’ prediction speed (the judgment-accuracy data indicated that there was no speed-accuracy trade-off in the response-time data).

However, the longer response latency at a faster rate in Experiment 1 may not necessarily result from slowed prediction speed. They could instead stem from impaired bottom-up processing in language comprehension (e.g., word recognition) under rapid speech, considering the slightly better judgment accuracy at a slower rate. To rule out this possibility, we needed an experiment that could disentangle the effects of speech rate on prediction from its effects on other aspects of comprehension.

## Experiment 2

To address this potential issue with Experiment 1, in Experiment 2, we had participants listen to fast- or slow-rate sentence fragments that either had a predictable ending (high-cloze) or did not (low-cloze) and then asked them to name a sentence-final picture corresponding to the target word. In the low-cloze condition, the unpredictable contexts prevented reliance on prediction and thus primarily engaged bottom-up comprehension. Additionally, the experimental procedure kept participants’ attention on the picture naming task, further minimizing predictive process during listening. Thus, naming latencies in the low-cloze condition, where facilitation from prediction was unlikely, served as a baseline to compare with the high-cloze condition, where successful prediction should facilitate naming. Comparing rate effects across these conditions allowed us to isolate the impact of speech rate on prediction from its effects on bottom-up comprehension.

### Method

#### Participants

One hundred and twenty undergraduates from Chinese universities (43 males and 77 females) aged 18 to 24 (*M* = 19.78 years, *SD* = 1.52), who were recruited online, took part in the experiment. All were native Mandarin Chinese speakers and gave informed consent. Each participant was paid ¥15 (about £1.48). The experiment was conducted online via Testable and was approved by the Ethics Committee of the Department of Psychology, University of Edinburgh.

#### Design

We used a 2 (Speech Rate: fast vs. slow) × 2 (Cloze Probability: high cloze vs. low cloze) within-subjects and within-item design. We assumed that picture naming would be facilitated if participants predicted the predictable words (in the high-cloze condition); therefore, the amount of facilitation in naming latency would depend on the extent and speed of participants’ prediction. In the low-cloze condition, where the final word was unpredictable and thus processing replied primarily on bottom-up comprehension, any difference in response latency between fast and slow speech rates would reflect the effect of speech rate on bottom-up comprehension. A longer response latency at a fast rate, compared to a slow rate, would suggest that the speeded speech rate slows down bottom-up comprehension processes. Conversely, if response latency remains unaffected or even decreases at a faster rate (potentially due to speech rate entrainment), it would indicate that bottom-up comprehension is not additionally taxed by increased rate.

In the high-cloze condition, where sentences were predictable, both bottom-up comprehension and prediction processes would be involved in the processing of the named word, with response latency reflecting the net effect of speech rate on the two processes. If analysis of the low-cloze condition reveals that a fast speech rate does not impair bottom-up comprehension and primarily induces speech rate entrainment (as evidenced by earlier response latency and shorter response duration), then longer response latency but shorter response duration (evidence for entrainment) at a faster rate in the high-cloze condition would indicate that the increased cognitive demands of prediction by the faster rate outweighs advantages of faster latency due to speech rate entrainment. This would suggest that the longer latency observed for predictable sentences at a faster rate in Experiment 1 was due to prediction rather than other aspects of comprehension, supporting the view that increased speech rates hinder prediction by leaving fewer resources available for a prediction process, assuming that prediction is cognitively demanding.

Alternatively, if no significant latency difference is observed between fast and slow rates in the high-cloze condition, but response duration is shorter at a faster speech (indicating speech rate entrainment), it would suggest that any advantages of faster latency due to speech rate entrainment are offset by the increased cognitive demands of prediction by the faster rate. This would still support the view that increased speech rates hinder prediction by leaving fewer resources available for such a process, assuming that prediction is cognitively demanding. We also expected shorter response latencies in the high- than the low-cloze conditions (due to facilitatory effects of prediction), and shorter response durations in the fast- than the slow-rate conditions (due to entrainment).

#### Materials

The experimental stimuli consisted of 96 pairs of high- and low-constraint sentence fragments matched with a target picture. To create the stimuli, we first constructed 120 sentence fragment pairs in written Chinese. In each pair, both fragments shared the same to-be-named picture (final word) but differed in content and contextual constraint, with one sentence fragment that we judged to be high constraint and the other that we judged to be low constraint (see Table 3). Forty additional participants from the same participant pool were paid ¥10 (about U.S.$1.50) to fill in the missing final (target) word for these fragments. The pairs were divided into two lists of 120 sentences containing one version of each pair (60 high-constraint and 60 low-constraint fragments). Twenty participants were asked to complete each list with the first word that came to mind. The cloze probability of a word was defined as the percentage of participants who used the exact word to complete the sentence. We selected 96 sentence fragment pairs for the main experiment and paired them with a target word (which was the most frequent completion in the high-constraint condition). This word had a mean cloze value of 94.80% (range 81.25%–100%) in the high-constraint condition, and a mean cloze value of 4.38% (range 0%–24%) in the low-constraint condition; see Table 3 for example stimuli and the Supplemental Material B for the compete set. In the selected items, the sentence-final targets were single-, two-, or three-character nouns (13, 73, and 10, respectively). We indexed word frequency with logWCount, the base-10 logarithm of WCount (total word tokens), from the SUBTLEX-CH corpus (Cai & Brysbaert, 2010). On the log_10_ scale, word frequency values ranged from 2.18 to 4.26 (*M* = 2.96, *SD* = 0.54). Following van Heuven et al. (2014), we treat values of 3 or below as indicating relatively low frequency.

**Table 3.** Example Sentence Fragments Used Experiment 2.

Close probability	Sentence context	Target word
High cloze	男朋友送了她一枚戒指，上面镶嵌着一颗闪闪发光的_ Nan Peng You Song Le Ta Yi Mei Jie Zhi, Shang Mian Xiang Qian Zhe Yi Ke Shan Shan Fa Guang De _ (The boyfriend gave her a ring studded with a sparkling _)	钻石 Zuan Shi Diamond
Low cloze	小偷趁主人不在家入室盗窃，偷走了放在保险柜里的_ Xiao Tou Chen Zhu Ren Bu Zai Jia Ru Shi Dao Qie, Tou Zou Le Fang Zai Bao Xian Xiang Li De_ (Thieves, taking advantage of the owner’s absence, broke into the safe and stole the_)	钻石 Zuan Shi Diamond

These sentence fragments were converted to speech using Voice Maker, at the same fast and slow rate as in Experiment 1, and thus both the fast and the slow versions were comprehensible and natural. In addition, there were 6 practice trials and 18 fillers similar to the experimental sentences (e.g., “There is no bridge over the river, so you will have to take a boat”) followed by a comprehension question (e.g., “To go across the river, do we have to take a boat?”), with half fillers requiring a “Yes” answer, and half a “No” answer. The fillers were to ensure that participants paid close attention to the recording.

Each pair of sentences was matched with a picture whose target name corresponded to the predictable word in the high-cloze fragment. The pictures were taken from Zhou and Chen (2017), who investigated naming latencies and norms in Mandarin Chinese for 435 images. We used this study because they included color and texture information, which can facilitate object processing and improve name agreement (Rossion & Pourtois, 2004), and the data were collected quite recently (in 2017). For all pictures that we selected, name agreement was at least 0.87, and two further Chinese native speakers both agreed they were easy to identify.

#### Procedure

**Figure 3.**
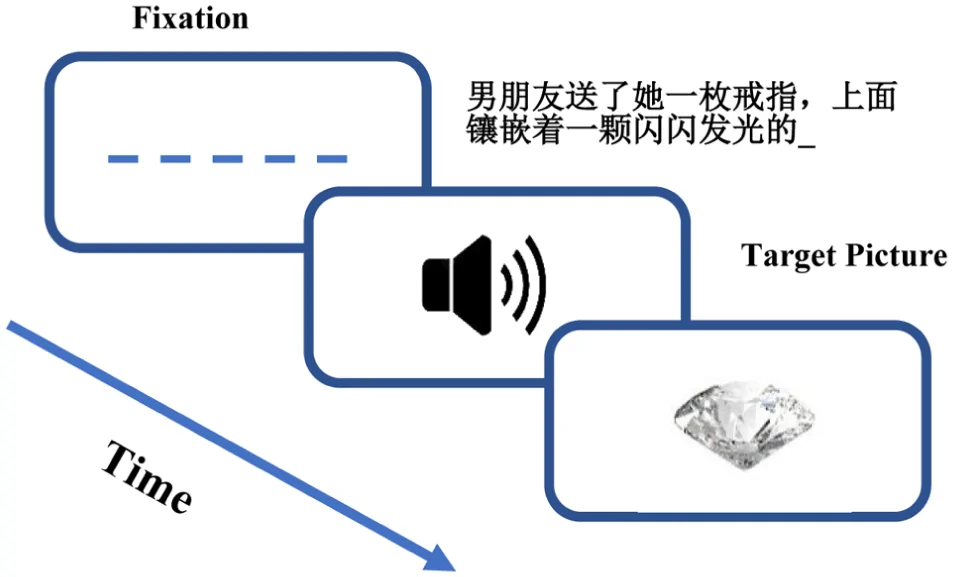
An example of a trial in Experiment 2.

An overview of the experimental paradigm is shown in Figure 3. The experiment was conducted online, and participants were asked to do it in a quiet room on a computer.

Participants were first instructed to listen to each sentence fragment. After the offset of each recording, a picture corresponding to the target word appeared on the screen, and they were asked to name the picture as quickly as possible. Each picture remained on the screen until the voice key detected a response. No feedback was given during the experiment. Stimuli were presented in two blocks of 60 trials each, with each block beginning with three practice trials, and a 5-min break between the blocks. Speech rate was blocked, and the block order of speech rate (fast vs. slow first) was counterbalanced between participants. The order of the sentences within each block was randomized for each participant and the whole experiment took approximately 30 min.

#### Data Analysis

Two participants were removed from further analyses for trials because more than 60% of the responses were wrong (0.80% of the data). Trials on which participants did not name the pictures correctly (4.08%) or response latencies were longer than 2,000 ms (1.68%) were eliminated. Furthermore, trials in which response latencies deviating more than 2 standard deviations from a participant’s conditional mean were excluded before further analysis (6.49%). Response durations for each trial were manually calculated using Audacity, an open-source audio software.

We used the lme4 package (version 1.1.26; Bates et al., 2015) in the statistical software R (version 4.0.5; R Core Team, 2021) to analyze the effects of speech rate and cloze probability (positive or negative) on response latency and response duration. The effects of speech rate and cloze probability on response latency and voice response duration were analyzed in two linear mixed effects models. The effects of speech rate and trial type in both models used sum contrast coding (Speech Rate: fast was set to −1, slow to 1; Cloze Probability: low was set to −1, high to 1). Because both response latency and duration had right-skewed distributions, we applied log transformations to these variables in our models. In both models, as fixed effects, we entered speech rate and cloze probability (with interaction term) into the model. As random effects, we specified the maximal random effects structure in both models, and if the maximal model did not converge, the model was progressively simplified in the order of removing random correlations, interactions of random effects, and random slopes corresponding to main effects, until the models converged (final model on response latency: lmer (log [RT] ~ SpeechRate * Cloze + [SpeechRate + Cloze | participant] + [SpeechRate + Cloze | picture] + [1|sentence-context], control = lmerControl [optimizer = “bobyqa”]); final model on response duration: lmer (log [duration] ~ SpeechRate * Cloze + [SpeechRate + Cloze | participant] + [SpeechRate + Cloze | picture] + [1|sentence-context], control = lmerControl [optimizer = “bobyqa”]). Significance was calculated using the lmerTest package (Kuznetsova et al., 2017). To control the family-wise error rate within the experiment, we applied Holm-Bonferroni correction to the set of planned fixed-effect tests within the experiment; effects were considered significant if their adjusted *p*-value was <.05.

### Results

#### Response Latency

**Figure 4.**
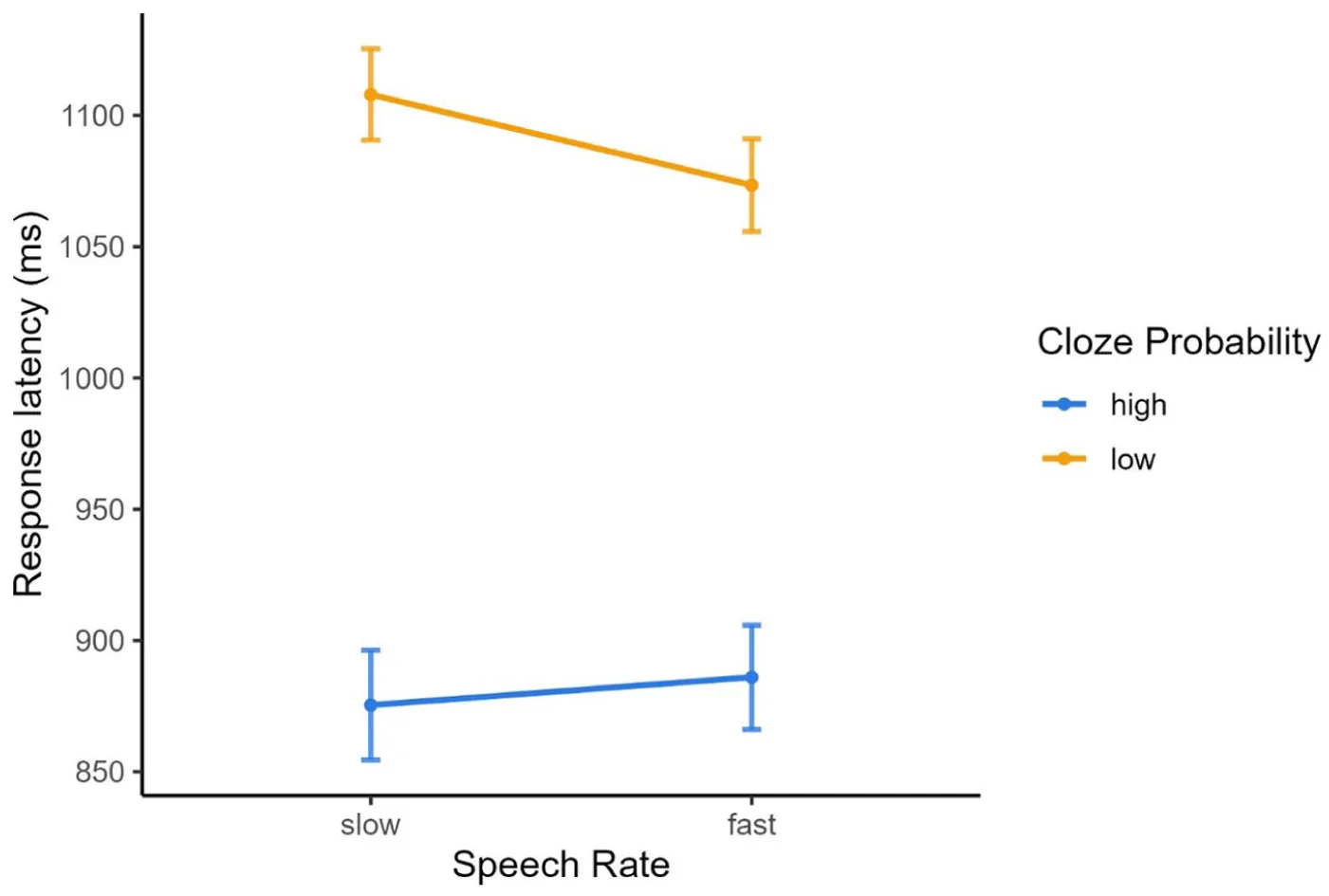
Mean response latencies (ms) in the four conditions in Experiment 2. *Note.* Error bars represent the standard error of the mean, aggregated by participants.

**Figure 5.**
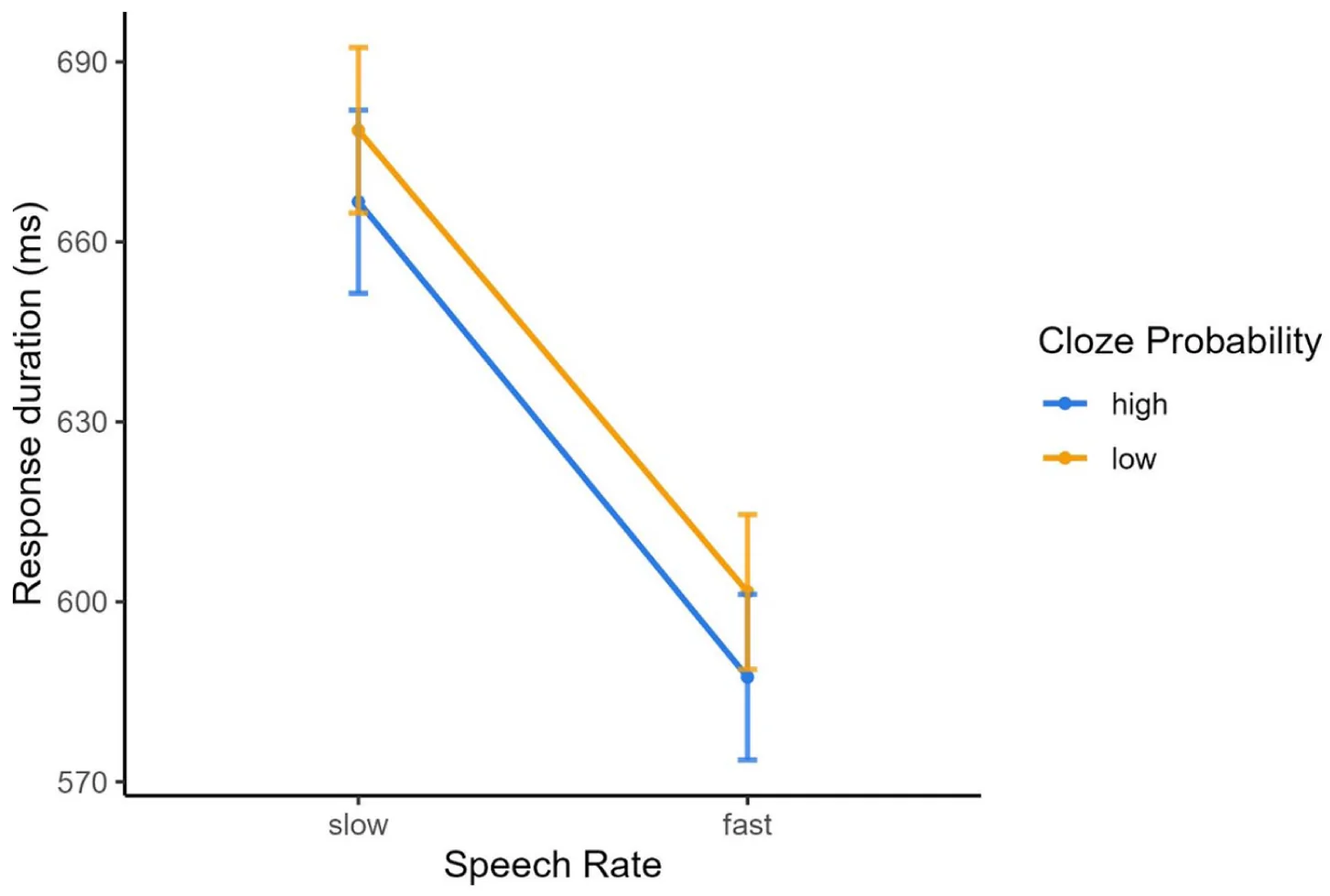
Mean response duration (ms) in the four conditions in Experiment 2. *Note.* Error bars represent the standard error of the mean, aggregated by participants.

Table 4 lists the means and standard deviations of response latency (see also Figure 4) and response durations (see also Figure 5) aggregated over participants in Experiment 2. For latency, there was an effect of cloze probability (*B* = −0.13, SE = 0.01, *t* = −14.79, *p* < .001): Participants responded faster in the high- than the low-cloze condition. There was no effect of speech rate (*B* = −0.00, SE = 0.01, *t* = −0.03, *p* = .974).^3^ However, there was an interaction between speech rate and cloze probability (*B* = −0.02, SE = 0.00, *t* = −6.28, *p* < .001). Additional analyses indicated that participants responded faster when the speech rate was fast than slow in the low-cloze condition (*B* = 35.3, SE = 13.2, *t* = 2.67, *p* = .012) but that there was no difference between fast and slow speech rate in the high-cloze condition (*B* = −10.2, SE = 13.2, *t* = −0.78, *p* = .500).

**Table 4.** Means (and Standard Deviations) for Response Latency and Response Duration in Experiment 2.

Condition	Response latency (milliseconds), mean (*SD*)	Response duration (milliseconds), mean (*SD*)
High cloze: slow speech	875 (227)	667 (166)
High cloze: fast speech	886 (215)	587 (149)
Low cloze: slow speech	1,108 (189)	679 (150)
Low cloze: fast speech	1,073 (191)	602 (140)

#### Response Duration

To determine whether there was speech rate entrainment, we further analyzed the effect of speech rate and cloze probability on response durations. There was an effect of speech rate (*B* = 0.06, SE = 0.01, *t* = 7.77, *p* < .001): The response duration was shorter when the speech rate was fast than slow. There was also an effect of cloze probability (*B* = −0.01, SE = 0.00, *t* = −4.16, *p* < .001): The response duration was shorter when sentences were predictable than unpredictable. But there was no interaction between speech rate and cloze probability (*B* = 0.00, SE = 0.00, *t* = 0.36, *p* = .716).

### Discussion

Experiment 2 did not show an overall main effect of speech rate on participants’ response latency. However, we observed an interaction between speech rate and cloze probability. Specifically, in the low-cloze condition, participants responded faster at the fast than slow rate. But there was no effect of speech rate on response latency in the high-cloze condition. As expected, we also found that participants’ response duration was shorter when the speech rate was fast than slow and when sentences were predictable than unpredictable.

In the low-cloze condition, sentences were unpredictable, so picture naming could not be facilitated by prediction. The primary effect of the fast speech rate was therefore rate entrainment, as evidenced by the shorter response duration at this quicker rate. This finding provides a baseline for understanding the effect of the faster speech rate on bottom-up comprehension alone, which then allows for comparison with naming conditions involving both bottom-up comprehension and prediction processes.

In the high-cloze condition, both bottom-up comprehension and prediction contributed to the picture naming, so we interpret the observed latency as the combined impact of speech rate on these two processes. We believed that the lack of a significant effect of speech rate on latency could be due to two counteracting effects: faster rate shortens response latencies via rate entrainment, but simultaneously increases the cognitive demands of prediction, which lengthens latencies. The absence of a significant latency difference suggests that these opposing influences largely canceled each other out, consistent with the view that prediction is resource-demanding. Notably, the descriptive pattern (a slight 11 ms increase in latency at the faster rate) suggests that the increased cognitive load from a faster speech rate was of a broadly similar magnitude as the benefits of reduced latency through entrainment.

The design of Experiment 2 may have encouraged participants to focus more on picture naming (as a bottom-up task), potentially reducing the engagement of the active top-down prediction process, even in highly predictable contexts. This, in turn, may have made it more difficult to detect an effect of speech rate on prediction speed. In contrast, the effect of speech rate might be easier to observe when the experimental task itself fully engages participants with the prediction process. Thus, we conducted Experiment 3, in which we presented participants with sentence fragments and asked them to produce a final word as quickly as possible—a task that allows for the full engagement of the prediction mechanism and provides a more direct test of speech rate effect on prediction speed.

## Experiment 3

We again had participants listen to a sentence fragment that was presented at a fast or slow rate. To further investigate how contextual predictability modulates the effect of speech rate on prediction speed, the sentences had either a highly predictable (high-cloze) or a less predictable (medium-cloze) ending, and participants were told to produce what they thought the final word would be while listening. We used medium- rather than low-cloze sentences because our design required participants to produce a particular word on a large fraction of trials–something that would happen in medium-cloze sentences but not in low-cloze sentences. Our main claims relate to trials on which participants did produce this word. We expected that the response latency for the predictable word should be longer in the fast- than the slow-rate condition, and this effect should be greater in the high- than the medium-cloze condition. We also expected shorter response durations in the fast- than the slow-rate condition.

### Method

#### Participants

One hundred and twenty Chinese undergraduates (55 males and 65 females, aged 18 to 24, *M* = 20.17 years, *SD* = 1.4) recruited from the same pool as in Experiment 2 took part in the experiment. All were native Chinese speakers and were given informed consent. Each participant was paid ¥15 (about £1.68) for participating in the experiment. Like Experiment 1 and Experiment 2, Experiment 3 was also conducted online via Testable and was approved by the Ethics Committee of the Department of Psychology, University of Edinburgh.

#### Materials

In Experiment 3, we added some medium cloze (35%–65%) sentence contexts to the materials used Experiment 2. We first constructed 120 medium-cloze sentence fragments whose highest-frequency continuations correspond to the target words of the 120 high-cloze sentence fragments used in the first experiment. Then we recruited a group of 20 undergraduates from different Chinese universities to perform the cloze probability test. All these undergraduates were Chinese native speakers, and each of them was paid ¥10 (about U.S.$1.5) for the cloze probability task. They were presented with the 120 sentence fragments and were asked to complete the sentence with the first word that came to their mind. The cloze probability of a word was defined as the percentage of participants who used the word to complete the sentence. We excluded items if this word was not the target word, or if it had a cloze probability of greater than 65% or lower than 35% (and note that the target word was always the highest-frequency word).

In total, 84 sentence fragment pairs were used in the third experiment. In each pair, both fragments shared the same to-be-named final word but differed in content and contextual constraint, with one sentence high constraint and the other medium constraint. The high constraint fragments had a mean cloze value of 91% (range 81%–100%), and the medium constraint fragments had a mean cloze value of 51% (range 35%–65%); see Table 5 for example stimuli and the Supplemental Material C for the compete set. In all selected items, the sentence-final target was a single-character noun, and the frequency values, indexed with logCHR from the SUBTLEX-CH-CHR character file, ranged from 1.98 to 5.05 (*M* = 3.92, *SD* = 0.58). Following van Heuven et al. (2014), we treat values of 3 or below as indicating relatively low frequency. These sentence fragments were recorded via Voicemaker at two speech rates the same way as in Experiment 1 and Experiment 2, and thus both the fast and the slow versions were comprehensible and natural.

**Table 5.** Example Sentence Fragments Used in the Third Experiment.

Cloze probability	Sentence context	Target word
High cloze	夏天天气变化非常快，我们出门最好带一把_ Xia Tian Tian Qi Bian Hua Fei Chang Kuai, Wo Men Chu Men Zui Hao Dai Yi Ba_ (Summer weather changes quickly, so we’d better take an _)	伞 San Umbrella
Medium cloze	无论春夏秋冬，我每次出门都会随身携带_ Wu Lun Chun Xia Qiu Dong, Wo Mei Ci Chu Men Dou Hui Sui Shen Xie Dai_ (Whenever I go out, I will always carry an _)	伞 San Umbrella

#### Design

We used a 2 (Speech Rate: fast vs. slow) × 2 (Cloze Probability: High Cloze vs. Medium Cloze) within-subjects and within-item design.

#### Procedure

**Figure 6.**
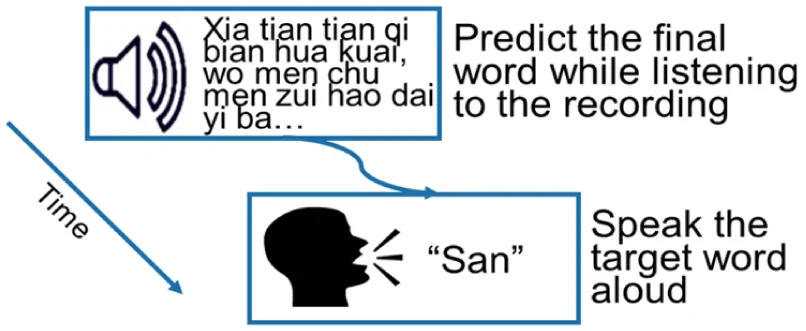
An example of a trial in Experiment 3.

Stimuli were presented in two blocks of 46 trials each. The first four trials in each block were practice trials. Participants were instructed to listen to sentence fragments and were told to actively predict the final word of the sentences while listening. After the offset of the recording, they were asked to speak the final word aloud as soon as possible, and the time to initiate a response was recorded. After they finished speaking the final word, the next trial began automatically. The experiment took about 30 min. An overview of the experimental paradigm is shown in Figure 6.

#### Data Analysis

Three participants were excluded from analyses because their recordings had loud audio noise (2.50%). Then we listened to each voice response and manually classified these responses into three categories: target responses, non-target responses, and errors. Target responses were responses in which participants produced the target word. Non-target responses were responses in which participants produced a different word. Errors were responses that were missing or unrecognizable. We excluded error trials (4.57%), trials in which response latencies were longer than 2,000 ms (9.66%), and trials in which response latencies were more than 2 *SD* away from a participant’s conditional mean (2.40%). The response duration in each trial was calculated using seewave package (Sueur et al., 2008) in R. Trials in which response durations were more than 2 *SD* away from a participant’s conditional mean (4.54%) were excluded from the analysis of response duration.

We analyzed dominant response proportion, response latency, and response duration in R (v4.0.5; R Core Team, 2021) using lme4 package (version 1.1.26; Bates et al., 2015). Dominant response proportion was modeled with a generalized linear mixed-effects model; response latency and duration were modeled with linear mixed-effects models. Because fast and slow trials were presented in separate blocks and the block order was counterbalanced across participants, we included block order and its interactions to assess potential order effects. Both effects in both models used sum contrast coding (Speech Rate: fast was set to −1, slow to 1; Cloze Probability: medium was set to −1, high to 1). As the distributions of response latency and response duration were right-skewed, we applied a log transformation to the dependent variable in our models.

In our model, as fixed effects, we entered speech rate, cloze probability (with interaction term), and block order (with interaction term) into the models. As random effects, we specified the maximal random effects structure in both models, and if the maximal model did not converge, complexity was removed in the order of removing random correlations, interactions of random effects, and random slopes corresponding to main effects until models converged. Significance was calculated using the lmerTest package (Kuznetsova et al., 2017). As in Experiments 1 and 2, we applied Holm-Bonferroni correction to the set of planned fixed-effect tests; effects were considered significant if their adjusted *p*-value was <.05.

Final model on dominant response proportion: glmer (Dominant~ SpeechRate * Cloze + [SpeechRate + Cloze | participant] + [Cloze | target-word] + [1|sentence-context], family = “binomial,” control = glmerControl [optimizer = “bobyqa”]);

Final model on response latency: lmer (log [RT] ~ SpeechRate * Cloze * BlockOrder + [SpeechRate | participant] + [1 | target-word] + [1|sentence-context], control = lmerControl [optimizer = “bobyqa”]);

Final model on response duration: lmer (log[duration] ~ SpeechRate * Cloze + [1 | participant] + [SpeechRate | target-word] + [1|sentence-context], control = lmerControl [optimizer = “bobyqa”]).

### Results

We analyzed the effects of speech rate and cloze probability on three dependent variables: proportion of target responses, response latency, and response duration.

#### Proportion of Target Responses

Table 6 lists the means and standard deviations of target response proportions aggregated over participants across different conditions. There was no effect of speech rate on the proportion of target responses (*B* = −0.07, SE = 0.04, *z* = −1.78, *p* = .377), but there was a main effect of cloze probability (*B* = 1.64, SE = 0.13, *z* = 12.78, *p* < .001), with a higher proportion in the high-cloze condition than the medium-cloze condition. There was no interaction between speech rate and cloze probability (*B* = 0.02, SE = 0.04, *z* = 0.47, *p* = .518). Note that the mean values are similar to the mean values in the pre-test.

**Table 6.** Means (and Standard Deviations) Aggregated Over Participants for Target Response Proportion in Experiment 3.

Condition	Proportion of target response (%)	
	Mean	*SD*
High cloze: fast speech	86.9	15.7
Medium cloze: fast speech	47.8	19.6
High cloze: slow speech	88.1	16.6
Medium cloze: slow speech	46.8	19.4

#### Response Latency

**Figure 7.**
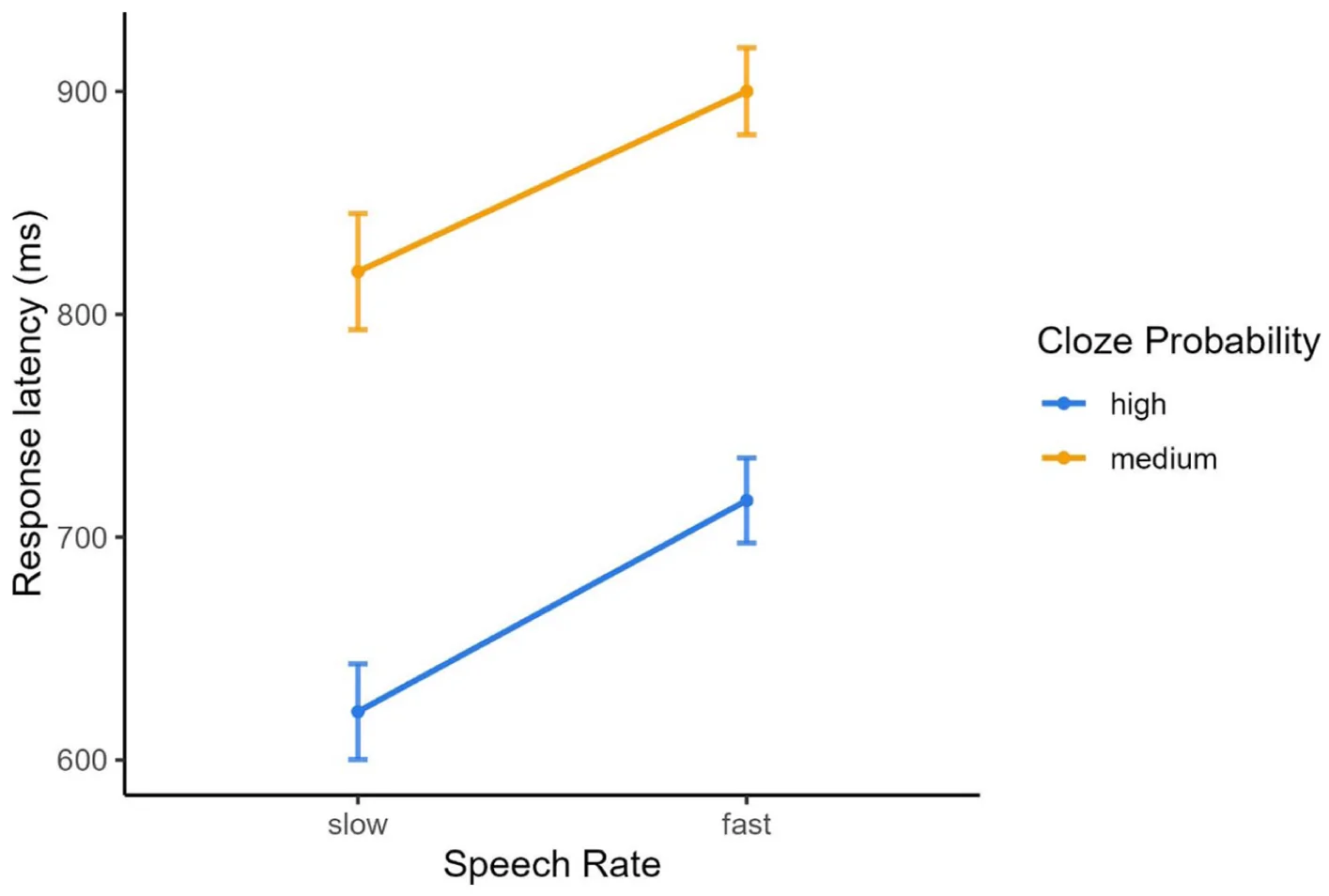
Mean response latencies (ms) in the four conditions in Experiment 3. *Note.* Error bars represent the standard error of the mean, aggregated by participants.

**Figure 8.**
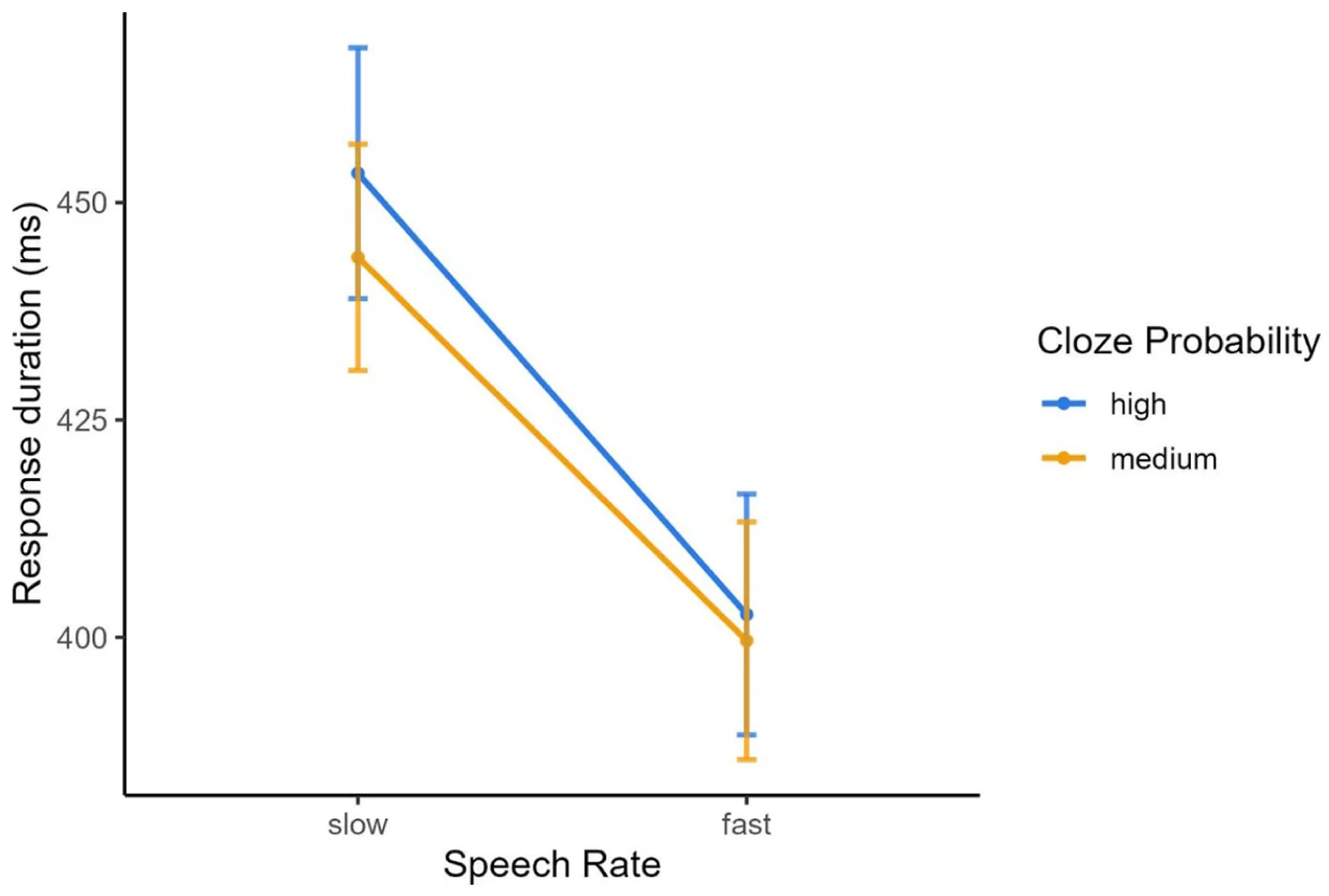
Mean response duration (ms) in the four conditions in Experiment 3. *Note.* Error bars represent the standard error of the mean, aggregated by participants.

We conducted two separate analyses of response latencies. The primary analysis considered trials on which participants produced the target word, because such responses were the same across conditions.^4^ Table 7 lists the means and standard deviations of response latencies^5^ (see also Figure 7) as well as response durations (see also Figure 8) aggregated over participants for trials in which participants produced the target word. There was an effect of speech rate^6^ on response latency (*B* = −0.07, SE = 0.01, *t* = −4.52, *p* < .001), with longer latencies in the fast- than the slow-rate condition. There was also an effect of cloze probability (*B* = −0.11, SE = 0.02, *t* = −5.99, *p* < .001), with longer latencies in the medium- than the high-cloze condition. In addition, there was an interaction between speech rate and cloze probability (*B* = −0.02, SE = 0.01, *t* = −3.91, *p* < .001). Simple effects showed that within medium-cloze contexts, fast speech led to longer response latency than slow speech (slow/fast ratio = 0.907, *t* = −3.02, *p* = .003, Holm-adjusted). Within high-cloze contexts, fast speech also produced longer latencies than slow speech, but the fast rate effect was larger than in medium-cloze contexts (slow/fast ratio = 0.818, *t* = −6.89, *p* < .001, Holm-adjusted).

**Table 7.** Means (and Standard Deviations) Aggregated Over Participants for Response Latency^a^ and Rsponse Duration When Participants Spoke a Target Word in Experiment 3.

Condition	Response latency (milliseconds), mean (*SD*)	Response duration (milliseconds), mean (*SD*)
High cloze: slow speech	622 (232)	453 (151)
High cloze: fast speech	716 (207)	403 (147)
Medium cloze: slow speech	819 (282)	444 (139)
Medium cloze: fast speech	900 (210)	400 (145)

#### Response Duration

We conducted an analysis of response duration for trials in which participants produced the target word. There was an effect of speech rate (*B* = 0.08, SE = 0.01, *t* = 10.10, *p* < .001), with longer durations in the slow- than the fast-rate condition. But there was no effect of cloze probability (*B* = 0.01, SE = 0.01, *t* = 1.50, *p* = .695) and no interaction (*B* = 0.01, SE = 0.00, *t* = 1.34, *p* = .716) between speech rate and cloze probability.

It is possible that participants took longer to initiate a response at the faster speech rate because they needed longer to plan the shorter-duration words that tended to occur in this condition (though we do not know of any evidence for this claim). To investigate this possibility, we included response duration as a covariate in the model on the effects of speech rate and cloze probability on response latency (Final model: lmer (log[RT] ~ speechrate * cloze + duration + [speechrate | participant] + [speechrate | target_word] + [1 | sentence_ context], control = lmerControl [optimizer = “bobyqa”]). However, we did not find an effect of response duration on response latency (*B* = 0.00, SE = 0.00, *t* = 1.19, *p* = .234), and there was still an effect of speech rate on response latency (*B* = −0.08, SE = −0.01, *t* = −3.78, *p* < .001) when response duration was included. Thus, the longer initiation time in the fast-rate condition did not appear to be due to the longer preparation time when articulation was fast.

### Discussion

As in Experiment 1, response latencies were longer in the fast- than the slow-rate condition, and more importantly, the effect of speech rate was greater in the high- than the medium-cloze condition. We also found that participants were more likely to produce a target response in the high- than medium-cloze condition (and the results were similar to the pre-test), but speech rate had no effect on the proportion of target responses. Participants responded earlier in the higher than the medium cloze condition, suggesting that highly predictable words are more quickly pre-activated than less predictable words. Response durations were also shorter in the fast- than the slow-rate condition, consistent with the results of Experiment 2.

The analysis of the target responses showed that participants exhibited a longer response latency to name a predictable word when they heard its context being presented at a fast than a slow speech rate. Thus, the results are compatible with Experiments 1 and 2, which also revealed that a fast rate lengthened time to respond. Participants also responded earlier in the higher than the medium cloze condition, suggesting that highly predictable words are more quickly pre-activated than less predictable words. More importantly, there was an interaction between speech rate and cloze probability, with the faster speech rate increasing response latency to a greater extent for more predictable than for less predictable sentence contexts.

## General Discussion

We investigated how comprehenders’ prediction speed is affected by speech rate and contextual predictability using three different experimental paradigms: a letter-judgment task to first determine the effect of a fast speech rate on prediction (Experiment 1), a picture naming task to distinguish whether the observed fast speech rate effect in highly predictable contexts was due to prediction or other aspects of comprehension (Experiment 2), and a word naming task to investigate how the effect of a fast speech rate on prediction was modulated by contexts of different predictability (Experiment 3). We found that a fast speech rate led to longer response latencies than a slow speech rate in Experiments 1 and 3, with this rate effect being larger in more predictable than less predictable contexts (Experiment 3). In Experiment 2, there was an interaction between speech rate and cloze probability: In the low-cloze condition, participants responded faster at the fast than at the slow rate, whereas in the high-cloze condition, speech rate had no effect on response latency. In both Experiments 2 and 3, response durations were shorter for fast than for slow speech.

### Effect of Fast Speech Rate on Predictive Processing

Despite using different paradigms, our three experiments provide a compatible set of findings. In Experiment 1, participants heard sentence fragments whose final word was highly predictable (high-cloze). After the offset of each fragment, they judged whether a given letter was contained in the Pinyin of the predictable word (and then typed the word they had predicted to verify their prediction). This task required pre-activation of the target, and the response latency was therefore taken as an indicator of prediction speed. Participants responded more slowly at the fast speech rate. However, the longer latencies at the faster rate in these highly predictable sentences may not necessarily reflect slower prediction per se; they could also arise from degraded bottom-up processing (i.e., difficulty comprehending), or from a combined effect of entrainment and prediction.

Experiment 2 addressed this concern by presenting participants with high- or low-constraint sentence fragments followed by picture naming of the sentence-final target. This design separates the effects of speech rate on prediction from the effects on bottom-up comprehension. In low-cloze contexts, unpredictable contexts prevented prediction from aiding naming, and pictures could be named irrespective of prior prediction, minimizing the top-down influence of prediction. Accordingly, the fast-slow latency difference in low-cloze contexts served as an entrainment baseline for bottom-up processing.

As expected, participants showed speech-rate entrainment: response durations were shorter at the fast than the slow speech rate, consistent with previous findings (Cohen Priva et al., 2017; Dilley & Pitt, 2010; Jungers & Hupp, 2009; Schultz et al., 2016). Such entrainment presumably caused listeners to initiate earlier response latencies at the fast rate in low-cloze contexts. In high-cloze contexts, however, the entrainment-related speed-up at the fast rate was offset by prediction-related costs (increased processing load), producing little to no latency difference between the fast and slow rates. Together, this pattern suggests that slower responses at the fast rate in Experiment 1 reflect costs to prediction rather than to other comprehension processes.

Experiment 3 used an online cloze task that requires greater engagement of predictive mechanism than Experiment 2: participants heard high- or medium-constraint fragments and were instructed to speak the final target word as quickly as possible. As in Experiment 1, the fast rate produced longer latencies than the slow rate, and crucially, this increase was larger in predictable (high-cloze) contexts than in less predictable (medium-cloze) contexts. This aligns with previous finding that faster speech (presentation) rate led to reduced predictive effect (Huettig & Guerra, 2019; Ito et al., 2016), suggesting that faster speech impairs prediction, and that the more the prediction system is engaged, the greater the impairment. When prediction is strongly engaged, the detrimental effect of fast speech on prediction can outweigh its entrainment-related speed-up, masking the entrainment effect.

### Implications for Prediction Mechanism

The impaired prediction at a faster speech rate, and especially greater impairment of fast speech in more predictable than less predictable contexts, aligns with the accounts that argue that prediction is cognitively demanding (Dell & Chang, 2014; F. Chang et al., 2006; Federmeier, 2007; Huettig & Mani, 2016; Pickering & Gambi, 2018; Pickering & Garrod, 2013). This interpretation is consistent with previous studies showing that the availability of cognitive resources, manipulated through a secondary task, can affect comprehenders’ predictions (Federmeier et al., 2010; Huettig & Janse, 2016; Ito et al., 2018).

We interpret our findings within Pickering and Gambi’s (2018) production-based prediction account: Comprehenders derive the speaker’s intention by covertly imitating them, and then run this intention through their production system to produce the representations of the predictable word in the order of meaning, syntax, and form. Assuming this process is resource-intensive and optional, taxing conditions (here, faster speech) leave fewer resources for prediction, resulting in slower predictive processing.

The interaction we observed—fast speech slowing responses more after highly predictable contexts than less predictable contexts—matches results from explicit load manipulations with a secondary task (Schuckart et al., 2024), where the cognitive load effect is larger in more predictable contexts. Our interpretation is that the prediction mechanism is engaged more in highly predictable contexts (where accurate prediction is more feasible) than less predictable contexts; therefore, any rate-induced resource reduction has a bigger impact in high cloze contexts. In short, because prediction draws on limited resources, faster speech reduces what is available and amplifies the rate effect on prediction in high-cloze contexts relative to lower-cloze contexts.

Although speeded speech appeared to slow down comprehenders’ prediction speed, it is important to note that our findings are not informative about the sub-processes through which the prediction process was slowed down by the fast speech rate. For instance, it is conceivable that the increased speech rate might impede the pre-activation of the semantic aspects of a predictable word, while leaving the pre-activation of the word’s syntactic or form aspects unaffected. Conversely, it could potentially influence all stages of the prediction process. To gain more clarity, future research could investigate whether the impact of speech rate encompasses all these processes or selectively targets individual sub-processes.

Note that the slow speech rate used in our study (2.42 syllables/second) is quite slow and may itself have increased cognitive load (Kemper & Harden, 1999). Fernandez et al. (2020) report greater predictive behavior at 4.5 than at 3.5 syllables/second, with a decline at 6.0 syllables/second, suggesting that there is an optimal speech rate for prediction. Our slow rate may lie to the left of this peak, and therefore be suboptimal. Nevertheless, our results showed that participants predicted more slowly at the fast than at the slow rate, suggesting that although the potentially suboptimal slow rate may have imposed some load, the difficulty associated with the fast rate (6.36 syllables/second) exceeded any difficulty due to the slow rate.

## Conclusion

We reported three experiments testing how fast speech rate affects prediction speed. Across tasks, we found that participants slowed down their response latency at a faster speech rate, and this effect was larger more predictable than less predictable contexts. Crucially, we demonstrated that the rate effect reflects prediction rather than other aspects of comprehension. Together, these findings suggest that a faster speech rate slows down prediction, supporting the view that prediction is resource-demanding.

## Supplemental Material

sj-docx-1-qjp-10.1177_17470218261446782 – Supplemental material for Faster Speech Slows Predictive Processing: Evidence from Mandarin Supplemental material, sj-docx-1-qjp-10.1177_17470218261446782 for Faster Speech Slows Predictive Processing: Evidence from Mandarin by Huanhuan Yin, Patrick Sturt and Martin J. Pickering in Quarterly Journal of Experimental Psychology

## Notes

1. An analysis by block order suggested that semantic similarity effect did not occur at 250 ms when this block came first, but did occur when it came after the 500 ms block.

2. There was an interaction between speech rate and block order (*B* = 0.04, SE = 0.01, *z* = 7.93, *p* < .001). When slow speech was presented before fast speech, participants took longer to respond at the slow than the fast rate (fast/slow = 1.126, *z* = 8.27, *p* = .003, Holm-adjusted); when fast speech was presented first, participants took longer to respond at fast than slow (slow/fast = 0.959, *z* = −2.94, *p* = .003, Holm-adjusted).

3. We also checked for a block order effect. There was no main effect of block order (β = −.01, SE = 0.01, *t* = −0.32, *p* = .748) and no interaction between speech rate and block order after applying the multiple-comparisons correction (β = −.01, SE = 0.00, *t* = −1.95, *p* = .208).

4. However, we also considered trials in which participants produced non-target responses. Since there were so few non-target responses in the high-cloze condition, we analyzed the non-target responses in only the medium-cloze condition. The mean response latencies in the fast and slow conditions were 1,587 and 1,528 ms, respectively. For these non-target responses, we did not find an effect of speech rate (*B* = −0.02, SE = 0.02, *t* = −0.86, *p* = .39).

5. The shorter mean response latencies in Experiment 3 compared to Experiment 2 provide additional support for the idea that participants in Experiment 2 may have focused primarily on the picture naming process, passively waiting for the speech to end before initiating their response, rather than actively engaging in top-down prediction. In contrast, Experiment 3 required participants to predict and produce the final word of a sentence fragment as quickly as possible, which likely encouraged earlier lexical preparation through active prediction. This early engagement of the prediction mechanism may have contributed to the reduced response latencies observed in Experiment 3.

6. We also checked for a block order effect. There was no main effect of block order (β = −.01, SE = 0.02, *t* = 0.19, *p* = .849) and no interaction between speech rate and block order after applying the multiple-comparisons correction (β = .032, SE = 0.01, *t* = 2.20, *p* = .208).

## References

[bibr1-17470218261446782] Allison C. Huettig F. Fernandez L. Lachmann T. (2025). Visuospatial working memory load reduces semantic prediction in the visual world. Language, Cognition and Neuroscience, 40(9), 1252–1261.

[bibr2-17470218261446782] Altmann G. T. M. Kamide Y. (1999). Incremental interpretation at verbs: Restricting the domain of subsequent reference. Cognition, 73(3), 247–264. 10.1016/S0010-0277(99)00059-1 10585516

[bibr3-17470218261446782] Arai M. Keller F. (2013). The use of verb-specific information for prediction in sentence processing. Language and Cognitive Processes, 28(4), 525–560. 10.1080/01690965.2012.658072

[bibr4-17470218261446782] Barr D. J. Levy R. Scheepers C. Tily H. J. (2013). Random effects structure for confirmatory hypothesis testing: Keep it maximal. Journal of Memory and Language, 68(3), 10.1016/j.jml.2012.11.001. 10.1016/j.jml.2012.11.001 PMC3881361 24403724

[bibr5-17470218261446782] Bates D. Mächler M. Bolker B. Walker S. (2015). Fitting linear mixed-effects models using lme4. Journal of Statistical Software, 67, 1–48. 10.18637/jss.v067.i01

[bibr6-17470218261446782] Brothers T. Swaab T. Y. Traxler M. J. (2017). Goals and strategies influence lexical prediction during sentence comprehension. Journal of Memory and Language, 93, 203–216. 10.1016/j.jml.2016.10.002

[bibr7-17470218261446782] Cai Q. Brysbaert M. (2010). SUBTLEX-CH: Chinese word and character frequencies based on film subtitles. PLoS One, 5(6), e10729. 10.1371/journal.pone.0010729 PMC2880003 20532192

[bibr8-17470218261446782] Chang A. C.-S. (2018). Speech Rate in Second Language Listening. In The TESOL encyclopedia of english language teaching (pp. 1–6). John Wiley & Sons, Ltd.

[bibr9-17470218261446782] Chang F. Dell G. S. Bock K. (2006). Becoming syntactic. Psychological Review, 113(2), 234. 10.1037/0033-295X.113.2.234 16637761

[bibr10-17470218261446782] Cohen Priva U. Edelist L. Gleason E. (2017). Converging to the baseline: Corpus evidence for convergence in speech rate to interlocutor’s baseline. The Journal of the Acoustical Society of America, 141(5), 2989–2996. 10.1121/1.4982199 28599520

[bibr11-17470218261446782] Corps R. E. Brooke C. Pickering M. J. (2022). Prediction involves two stages: Evidence from visual-world eye-tracking. Journal of Memory and Language, 122, 104298.

[bibr12-17470218261446782] Dave S. Brothers T. A. Traxler M. J. Ferreira F. Henderson J. M. Swaab T. Y. (2018). Electrophysiological evidence for preserved primacy of lexical prediction in aging. Neuropsychologia, 117, 135–147. 10.1016/j.neuropsychologia.2018.05.023 29852201

[bibr13-17470218261446782] Dell G. S. Chang F. (2014). The P-chain: Relating sentence production and its disorders to comprehension and acquisition. Philosophical Transactions of the Royal Society B: Biological Sciences, 369(1634), 20120394. 10.1098/rstb.2012.0394 PMC3866424 24324238

[bibr14-17470218261446782] DeLong K. A. Urbach T. P. Kutas M. (2005). Probabilistic word pre-activation during language comprehension inferred from electrical brain activity. Nature Neuroscience, 8(8), Article 8. 10.1038/nn1504 16007080

[bibr15-17470218261446782] Dikker S. Rabagliati H. Farmer T. A. Pylkkänen L. (2010). Early occipital sensitivity to syntactic category is based on form typicality. Psychological Science, 21(5), 629–634. 10.1177/0956797610367751 20483838

[bibr16-17470218261446782] Dilley L. C. Pitt M. A. (2010). Altering context speech rate can cause words to appear or disappear. Psychological Science, 21(11), 1664–1670. 10.1177/0956797610384743 20876883

[bibr17-17470218261446782] Federmeier K. D. (2007). Thinking ahead: The role and roots of prediction in language comprehension. Psychophysiology, 44(4), 491–505. 10.1111/j.1469-8986.2007.00531.x PMC2712632 17521377

[bibr18-17470218261446782] Federmeier K. D. Kutas M. (1999). A rose by any other name: Long-term memory structure and sentence processing. Journal of memory and Language, 41(4), 469–495.

[bibr19-17470218261446782] Federmeier K. D. Kutas M. Schul R. (2010). Age-related and individual differences in the use of prediction during language comprehension. Brain and Language, 115(3), 149–161. 10.1016/j.bandl.2010.07.006 PMC2975864 20728207

[bibr20-17470218261446782] Fernandez L. B. Engelhardt P. E. Patarroyo A. G. Allen S. E. (2020). Effects of speech rate on anticipatory eye movements in the visual world paradigm: Evidence from aging, native, and non-native language processing. Quarterly Journal of Experimental Psychology, 73(12), 2348–2361. 10.1177/1747021820948019 32705947

[bibr21-17470218261446782] Gambi C. Gorrie F. Pickering M. J. Rabagliati H. (2018). The development of linguistic prediction: Predictions of sound and meaning in 2- to 5-year-olds. Journal of Experimental Child Psychology, 173, 351–370. 10.1016/j.jecp.2018.04.012 29793772

[bibr22-17470218261446782] Garrod S. Pickering M. J. (2015). The use of content and timing to predict turn transitions. Frontiers in Psychology, 6, 751. 10.3389/fpsyg.2015.00751 PMC4463931 26124728

[bibr23-17470218261446782] Grisoni L. Miller T. M. Pulvermüller F. (2017). Neural correlates of semantic prediction and resolution in sentence processing. Journal of Neuroscience, 37(18), 4848–4858. 10.1523/JNEUROSCI.2800-16.2017 PMC5426574 28411271

[bibr24-17470218261446782] Heilbron M. Armeni K. Schoffelen J.-M. Hagoort P. de Lange F. P. (2022). A hierarchy of linguistic predictions during natural language comprehension. Proceedings of the National Academy of Sciences of the United States of America, 119(32), e2201968119. 10.1073/pnas.2201968119 PMC9371745 35921434

[bibr25-17470218261446782] Hoogland D. White L. Knight S. (2023). Speech rate and turn-transition pause duration in Dutch and English spontaneous question-answer sequences. Languages, 8(2), Article 2. 10.3390/languages8020115

[bibr26-17470218261446782] Huang Z. Feng C. Qu Q. (2023). Predicting coarse-grained semantic features in language comprehension: Evidence from ERP representational similarity analysis and Chinese classifier. Cerebral Cortex, 33(13), 8312–8320. 10.1093/cercor/bhad116 PMC10786091 37015899

[bibr27-17470218261446782] Huettig F. (2015). Four central questions about prediction in language processing. Brain Research, 1626, 118–135. 10.1016/j.brainres.2015.02.014 25708148

[bibr28-17470218261446782] Huettig F. Guerra E. (2019). Effects of speech rate, preview time of visual context, and participant instructions reveal strong limits on prediction in language processing. Brain Research, 1706, 196–208. 10.1016/j.brainres.2018.11.013 30439351

[bibr29-17470218261446782] Huettig F. Janse E. (2016). Individual differences in working memory and processing speed predict anticipatory spoken language processing in the visual world. Language, Cognition and Neuroscience, 31(1), 80–93. 10.1080/23273798.2015.1047459

[bibr30-17470218261446782] Huettig F. Mani N. (2016). Is prediction necessary to understand language? Probably not. Language, Cognition and Neuroscience, 31(1), 19–31. 10.1080/23273798.2015.1072223

[bibr31-17470218261446782] Ito A. Corley M. Pickering M. J. (2018). A cognitive load delays predictive eye movements similarly during L1 and L2 comprehension. Bilingualism: Language and Cognition, 21(2), 251–264. 10.1017/S1366728917000050

[bibr32-17470218261446782] Ito A. Corley M. Pickering M. J. Martin A. E. Nieuwland M. S. (2016). Predicting form and meaning: Evidence from brain potentials. Journal of Memory and Language, 86, 157–171. 10.1016/j.jml.2015.10.007

[bibr33-17470218261446782] Ito A. Gambi C. Pickering M. J. Fuellenbach K. Husband E. M. (2020). Prediction of phonological and gender information: An event-related potential study in Italian. Neuropsychologia, 136, 107291. 10.1016/j.neuropsychologia.2019.107291 31805283

[bibr34-17470218261446782] Iwarsson J. Naes J. Hollen R. (2021). Full article: Measuring speaking rate: How do objective measurements correlate with audio-perceptual ratings? Retrieved September 7, 2025, from https://www.tandfonline.com/doi/full/10.1080/4015439.2021.1988702 10.1080/14015439.2021.1988702 34672858

[bibr35-17470218261446782] Janse E. (2003). Production and perception of fast speech (LOT Dissertation Series 69) [Doctoral dissertation, Utrecht University]. LOT Publications.

[bibr36-17470218261446782] Jungers M. K. Hupp J. M. (2009). Speech priming: Evidence for rate persistence in unscripted speech. Language and Cognitive Processes, 24(4), 611–624. 10.1080/01690960802602241

[bibr37-17470218261446782] Kang C. Ma F. Li S. Kroll J. F. Guo T. (2020). Domain-general inhibition ability predicts the intensity of inhibition on non-target language in bilingual word production: An ERP study. Bilingualism: Language and Cognition, 23(5), 1056–1069. 10.1017/S1366728920000085

[bibr38-17470218261446782] Kemper S. Harden T. (1999). Experimentally disentangling what’s beneficial about elderspeak from what’s not. Psychology and Aging, 14(4), 656. 10.1037//0882-7974.14.4.656 10632152

[bibr39-17470218261446782] Koch X. Janse E. (2016). Speech rate effects on the processing of conversational speech across the adult life spana). The Journal of the Acoustical Society of America, 139(4), 1618–1636. 10.1121/1.4944032 27106310

[bibr40-17470218261446782] Kukona A. (2020). Lexical constraints on the prediction of form: Insights from the visual world paradigm. Journal of Experimental Psychology: Learning, Memory, and Cognition, 46(11), 2153. 10.1037/xlm0000935 32658542

[bibr41-17470218261446782] Kukona A. (2023). Predictive sentence processing at speed: Evidence from online mouse cursor tracking. Cognitive Science, 47(4), e13285. 10.1111/cogs.13285 37186474

[bibr42-17470218261446782] Kukona A. Cho P. W. Magnuson J. S. Tabor W. (2014). Lexical interference effects in sentence processing: Evidence from the visual world paradigm and self-organizing models. Journal of Experimental Psychology: Learning, Memory, and Cognition, 40(2), 326–347. 10.1037/a0034903 PMC4033295 24245535

[bibr43-17470218261446782] Kukona A. Fang S.-Y. Aicher K. A. Chen H. Magnuson J. S. (2011). The time course of anticipatory constraint integration. Cognition, 119(1), 23–42. 10.1016/j.cognition.2010.12.002 PMC3047461 21237450

[bibr44-17470218261446782] Kuperberg G. R. Jaeger T. F. (2016). What do we mean by prediction in language comprehension? Language, Cognition and Neuroscience, 31(1), 32–59. 10.1080/23273798.2015.1102299 PMC4850025 27135040

[bibr45-17470218261446782] Kuznetsova A. Brockhoff P. B. Christensen R. H. B. (2017). LmerTest Package: Tests in linear mixed effects models. Journal of Statistical Software, 82, 1–26. 10.18637/jss.v082.i13

[bibr46-17470218261446782] Lee P. S. K. Chan A. H. S. (2003). Chinese speaking times. International Journal of Industrial Ergonomics, 31(5), 313–321. 10.1016/S0169-8141(02)00222-6

[bibr47-17470218261446782] Liu S. Hu W. Liu X. (2025). Different effects of verbal and visual working memory loads on Language prediction. Scientific Reports, 15(1), 22432. 10.1038/s41598-025-03556-w PMC12216834 40595768

[bibr48-17470218261446782] Mani N. Huettig F. (2012). Prediction during language processing is a piece of cake—But only for skilled producers. Journal of Experimental Psychology: Human Perception and Performance, 38(4), 843. 10.1037/a0029284 22774799

[bibr49-17470218261446782] Müller J. A. Wendt D. Kollmeier B. Debener S. Brand T. (2019). Effect of speech rate on neural tracking of speech. Frontiers in Psychology, 10, 449. 10.3389/fpsyg.2019.00449 PMC6418035 30906273

[bibr50-17470218261446782] Pickering M. J. Gambi C. (2018). Predicting while comprehending language: A theory and review. Psychological Bulletin, 144, 1002–1044. 10.1037/bul0000158 29952584

[bibr51-17470218261446782] Pickering M. J. Garrod S. (2013). An integrated theory of language production and comprehension. Behavioral and Brain Sciences, 36(4), 329–347. 10.1017/S0140525X12001495 23789620

[bibr52-17470218261446782] R Core Team. (2021). R: A language and environment for statistical computing. R Foundation for Statistical Computing. https://www.R-project.org/

[bibr53-17470218261446782] Rossion B. Pourtois G. (2004). Revisiting snodgrass and vanderwart’s object pictorial set: The role of surface detail in basic-level object recognition. Perception, 33(2), 217–236. 10.1068/p5117 15109163

[bibr54-17470218261446782] Ryskin R. Nieuwland M. S. (2023). Prediction during language comprehension: What is next? Trends in Cognitive Sciences, 27(11), 1032–1052. 10.1016/j.tics.2023.08.003 PMC11614350 37704456

[bibr55-17470218261446782] Salthouse T. A. (1996). The processing-speed theory of adult age differences in cognition. Psychological Review, 103(3), 403–428. 10.1037/0033-295X.103.3.403 8759042

[bibr56-17470218261446782] Schuckart M. Martin S. Tune S. Schmitt L. M. Hartwigsen G. Obleser J. (2024). Limited cognitive resources reduce the language predictability benefit across the adult lifespan. bioRxiv. 10.1101/2024.05.08.592872 PMC12851580 41603353

[bibr57-17470218261446782] Schuckart M. Martin S. Tune S. Schmitt L.-M. Hartwigsen G. Obleser J. (2024). Limited cognitive resources reduce the language predictability benefit across the adult lifespan. 10.7554/eLife.108176 PMC12851580 41603353

[bibr58-17470218261446782] Schuckart M. M. Martin S. Tune S. Schmitt L. M. Hartwigsen G. Obleser J. (2026). Executive resources shape the impact of language predictability across the adult lifespan. eLife, 14, RP108176. 10.7554/eLife.108176 PMC12851580 41603353

[bibr59-17470218261446782] Schultz B. G. O’brien I. Phillips N. McFarland D. H. Titone D. Palmer C. (2016). Speech rates converge in scripted turn-taking conversations. Applied Psycholinguistics, 37(5), 1201–1220. 10.1017/S0142716415000545

[bibr60-17470218261446782] Shao X. Li M. Yang Y. Li X. Han Z. (2022). The neural basis of semantic prediction in sentence comprehension. Journal of Cognitive Neuroscience, 34(2), 236–257. 10.1162/jocn_a_01793 34813653

[bibr61-17470218261446782] Shen W. Hyönä J. Wang Y. Hou M. Zhao J. (2021). The role of tonal information during spoken-word recognition in Chinese: Evidence from a printed-word eye-tracking study. Memory & Cognition, 49(1), 181–192. 10.3758/s13421-020-01070-0 32676885

[bibr62-17470218261446782] Simantiraki O. Cooke M. (2020). Exploring Listeners’ Speech Rate Preferences. Interspeech 2020, 1346–1350. 10.21437/Interspeech.2020-1832

[bibr63-17470218261446782] Sueur J. Aubin T. Simonis C. (2008). Seewave, a free modular tool for sound analysis and synthesis. Bioacoustics, 18(2), 213–226. 10.1080/09524622.2008.9753600

[bibr64-17470218261446782] Tauroza S. Allison D. (1990). Speech rates in british english. Applied Linguistics, 11(1), 90–105.

[bibr65-17470218261446782] Torreira F. Bögels S. (2022). Vocal reaction times to speech offsets: Implications for processing models of conversational turn-taking. Journal of Phonetics, 94, 101175. 10.1016/j.wocn.2022.101175

[bibr66-17470218261446782] Van Berkum J. J. A. Brown C. M. Zwitserlood P. Kooijman V. Hagoort P. (2005). Anticipating upcoming words in discourse: Evidence from ERPs and reading times. Journal of Experimental Psychology: Learning, Memory, and Cognition, 31(3), 443–467. 10.1037/0278-7393.31.3.443 15910130

[bibr67-17470218261446782] van Heuven W. J. B. Mandera P. Keuleers E. Brysbaert M. (2014). Subtlex-UK: A New and Improved Word Frequency Database for British English. Quarterly Journal of Experimental Psychology, 67(6), 1176–1190. 10.1080/17470218.2013.850521 24417251

[bibr68-17470218261446782] Wicha N. Y. Y. Moreno E. M. Kutas M. (2003). Anticipating words and their gender: An event-related brain potential study of semantic integration, gender expectancy, and gender agreement in Spanish sentence reading. Cortex, 39(3), 483–508. 10.1016/S0010-9452(08)70260-0 PMC3380438 15453979

[bibr69-17470218261446782] Wicha N. Y. Y. Moreno E. M. Kutas M. (2004). Anticipating words and their gender: An event-related brain potential study of semantic integration, gender expectancy, and gender agreement in Spanish sentence reading. Journal of Cognitive Neuroscience, 16(7), 1272–1288. 10.1162/0898929041920487 PMC3380438 15453979

[bibr70-17470218261446782] Wilson M. Wilson T. P. (2005). An oscillator model of the timing of turn-taking. Psychonomic Bulletin & Review, 12(6), 957–968. 10.3758/BF03206432 16615316

[bibr71-17470218261446782] Winn M. B. Teece K. H. (2021). Slower speaking rate reduces listening effort among listeners with cochlear implants. Ear and Hearing, 42(3), 584–595. 10.1097/AUD.0000000000000958 PMC8005496 33002968

[bibr72-17470218261446782] Wlotko E. W. Federmeier K. D. (2015). Time for prediction? The effect of presentation rate on predictive sentence comprehension during word-by-word reading. Cortex, Special Issue: Prediction in Speech and Language Processing, 68, 20–32. 10.1016/j.cortex.2015.03.014 PMC4832567 25987437

[bibr73-17470218261446782] Wlotko E. W. Federmeier K. D. Kutas M. (2012). To predict or not to predict: Age-related differences in the use of sentential context. Psychology and Aging, 27(4), 975–988. 10.1037/a0029206 PMC3685629 22775363

[bibr74-17470218261446782] Yuan J. Liberman M. Cieri C. (2006). Towards an integrated understanding of speaking rate in conversation. Interspeech 2006, paper 1795-Mon3A3O.1-0. 10.21437/Interspeech.2006-204

[bibr75-17470218261446782] Zhou D. Chen Q. (2017). Color image norms in Mandarin Chinese. Frontiers in Psychology, 8, 1880. 10.3389/fpsyg.2017.01880 PMC5660975 29118730

[bibr76-17470218261446782] Zhao Z. Ding J. Wang J. Chen Y. Li X. (2024). The flexibility and representational nature of phonological prediction in listening comprehension: Evidence from the visual world paradigm. Language and Cognition, 16(2), 481–504. 10.1017/langcog.2023.38

