# Supplementary material for: Faster Speech Slows Predictive Processing: Evidence from Mandarin
Source: Q J Exp Psychol (Hove). 2026 Apr 20;79(10):2836–55. doi: 10.1177/17470218261446782 (PMC13601700; doi:10.1177/17470218261446782)
Supplement: Supplementary file 1 [file sj-docx-1-qjp-10.1177_17470218261446782.docx]

Supplementary Material for:

# **Faster Speech Slows Predictive Processing: Evidence from Mandarin**

Huanhuan Yin*, Patrick Sturt & Martin J. Pickering

Department of Psychology, University of Edinburgh, United Kingdom

*Address correspondence to: Huanhuan Yin, Department of Psychology, University of Edinburgh, 7 George Square, EH8 9JZ, Edinburgh, Scotland, United Kingdom.

E-mail address: H.Yin-9@sms.ed.ac.uk

**Stimuli for Experiment 1**

All 120 sentences from the experiment are listed below with the predictable character and its Pinyin. The mean cloze value of the target word is shown at the end of each sentence. Below each sentence are the letter judgement questions used in positive trials and negative trials respectively.

1. 今天是母亲节，我要去花店给母亲买（花hua）。(81%)

Today is Mother's Day. I am going to the flower shop to buy my mom flowers

您所预测的字的拼音中是否包含字母 "a" ?

您所预测的字的拼音中是否包含字母 "g" ?

Does the pinyin of your predicted word contain the letter "a"?

Does the pinyin of your predicted word contain the letter "g"?

2. 今天的天空特别蓝，上边还飘着几朵白（云yun）。(95% )

The sky is particularly blue today, with a few white clouds

您所预测的字的拼音中是否包含字母 "n" ?

您所预测的字的拼音中是否包含字母 "a" ?

Does the pinyin of your predicted word contain the letter "n"?

Does the pinyin of your predicted word contain the letter "a"?

3. 在大街上要注意文明，不要随地吐（痰tan）。(92% )

Be civilized on the street and don't spit

您所预测的字的拼音中是否包含字母 "n" ?

您所预测的字的拼音中是否包含字母 "e" ?

Does the pinyin of your predicted word contain the letter "n"?

Does the pinyin of your predicted word contain the letter "e"?

4. 夏天到了，女孩子们都喜欢穿上漂亮的裙子去逛（街jie）。(89% )

Summer is here, and girls like to wear beautiful skirts and go shopping in the street

您所预测的字的拼音中是否包含字母 "e" ?

您所预测的字的拼音中是否包含字母 "o" ?

Does the pinyin of your predicted word contain the letter "e"?

Does the pinyin of your predicted word contain the letter "o"?

5. 吃鱼的时候要特别小心，因为里面有很多（刺ci）。(86% )

Be especially careful when eating fish because there are many thorns

您所预测的字的拼音中是否包含字母 "i" ?

您所预测的字的拼音中是否包含字母 "a" ?

Does the pinyin of your predicted word contain the letter "i"?

Does the pinyin of your predicted word contain the letter "a"?

6. 因为我有糖尿病，所以我很少吃 (糖tang) 。( 83%)

Because I have diabetes, I rarely eat sugar.

您所预测的字的拼音中是否包含字母 "g" ?

您所预测的字的拼音中是否包含字母 "i" ?

Does the pinyin of your predicted word contain the letter "g"?

Does the pinyin of your predicted word contain the letter "i"?

7. 不是我不想买这件衣服，而是因为口袋里没有（钱qian）。(95% )

It's not that I don't want to buy this dress, but because I don't have (money)

您所预测的字的拼音中是否包含字母 "n" ?

您所预测的字的拼音中是否包含字母 "o" ?

Does the pinyin of your predicted word contain the letter "n"?

Does the pinyin of your predicted word contain the letter "o"?

8. 杰克写给安娜的情书很真诚，字里行间都充满了对她的（爱ai）。（84%）

Jack's love letter to Anna is very sincere, and the lines are full of love

您所预测的字的拼音中是否包含字母 "i" ?

您所预测的字的拼音中是否包含字母 "e" ?

Does the pinyin of your predicted word contain the letter "i"?

Does the pinyin of your predicted word contain the letter "e"?

9. 你要多吃饭多运动，不然会很容易生（病bing）。（89% ）

You have to eat more and exercise more, otherwise you will easily get sick

您所预测的字的拼音中是否包含字母 "g" ?

您所预测的字的拼音中是否包含字母 "u" ?

Does the pinyin of your predicted word contain the letter "g"?

Does the pinyin of your predicted word contain the letter "u"?

10. 这条河上没有桥，要去河对面必须要坐（船chuan）。（89% ）

To go to the other side of the river, you have to take a boat because there is no bridge on this river.

您所预测的字的拼音中是否包含字母 "n" ?

您所预测的字的拼音中是否包含字母 "i" ?

Does the pinyin of your predicted word contain the letter "n"?

Does the pinyin of your predicted word contain the letter "i"?

11. 李明是个素食主义者，他从来不吃（肉rou）。（92% ）

Li Ming is a vegetarian, he never eats (meat)

您所预测的字的拼音中是否包含字母 "u" ?

您所预测的字的拼音中是否包含字母 "a" ?

Does the pinyin of your predicted word contain the letter "u"?

Does the pinyin of your predicted word contain the letter "a"?

12. 小王打完篮球回来出了一身汗，所以第一件事就是去洗（澡zao）。（92% ）

Xiao Wang was sweating after playing basketball, so the first thing he did was to take a bath

您所预测的字的拼音中是否包含字母 "o" ?

您所预测的字的拼音中是否包含字母 "u" ?

Does the pinyin of your predicted word contain the letter "o"?

Does the pinyin of your predicted word contain the letter "u"?

13. 艾米莉今年拿到了驾照，打算明年给自己买一台(车che)。（92%）

Emily got her driver's license this year and next year plans to buy herself a car

您所预测的字的拼音中是否包含字母 "e" ?

您所预测的字的拼音中是否包含字母 "g" ?

 Does the pinyin of your predicted word contain the letter "e"?

Does the pinyin of your predicted word contain the letter "g"?

14. 夏天天气变化非常快，我们出门最好带一把(伞san)。（97% ）

The weather changes very quickly in summer, so when we go out we'd better bring an umbrella

您所预测的字的拼音中是否包含字母 "n" ?

您所预测的字的拼音中是否包含字母 "o" ?

Does the pinyin of your predicted word contain the letter "n"?

Does the pinyin of your predicted word contain the letter "o"?

15. 今天是中秋节，大家吃完饭后一起坐在院子里赏(月yue)。（95% ）

Today is the Mid-Autumn Festival. After eating, everyone sits in the yard to see the moon

您所预测的字的拼音中是否包含字母 "e" ?

您所预测的字的拼音中是否包含字母 "g" ?

Does the pinyin of your predicted word contain the letter "e"?

Does the pinyin of your predicted word contain the letter "g"?

16. 丽丽每天晚上都睡得很晚，因为他喜欢熬(夜ye)。（92% ）

Lili goes to bed very late every night because she likes to stay up late

您所预测的字的拼音中是否包含字母 "e" ?

您所预测的字的拼音中是否包含字母 "i" ?

Does the pinyin of your predicted word contain the letter "e"?

Does the pinyin of your predicted word contain the letter "i"?

17. 我姥姥生病了，我要去医院给她买(药yao)。（95% ）

My grandma is sick, and I'm going to the hospital to buy her medicine

您所预测的字的拼音中是否包含字母 "o" ?

您所预测的字的拼音中是否包含字母 "g" ?

Does the pinyin of your predicted word contain the letter "o"?

Does the pinyin of your predicted word contain the letter "g"?

18. 妈妈早上刚拖完地，进门请换(鞋xie)。（92%）

Mom just finished mopping the floor in the morning, so when entering the door please change your shoes.

您所预测的字的拼音中是否包含字母 "e" ?

您所预测的字的拼音中是否包含字母 "o" ?

Does the pinyin of your predicted word contain the letter "e"?

Does the pinyin of your predicted word contain the letter "o"?

19. 以前通信技术不发达，人们之间联系主要靠写(信xin)。（89% ）

A long time ago, the communication technology was underdeveloped, and the communication between people mainly depended on writing letters

您所预测的字的拼音中是否包含字母 "n" ?

您所预测的字的拼音中是否包含字母 "u" ?

Does the pinyin of your predicted word contain the letter "n"?

Does the pinyin of your predicted word contain the letter "u"?

20. 铃声响了，老师走进教室给同学们上(课ke)。（89% ）

The bell rang, and the teacher walked into the classroom to teach (lesson)

您所预测的字的拼音中是否包含字母 "e" ?

您所预测的字的拼音中是否包含字母 "a" ?

Does the pinyin of your predicted word contain the letter "e"?

Does the pinyin of your predicted word contain the letter "a"?

21. 早上吃的饭太咸了，现在我特别渴,想喝(水shui)。（92% ）

The meal I ate in the morning was too salty. Now I am very thirsty and want to drink (water)

您所预测的字的拼音中是否包含字母 "i" ?

您所预测的字的拼音中是否包含字母 "g" ?

Does the pinyin of your predicted word contain the letter "i"?

Does the pinyin of your predicted word contain the letter "g"?

22. 植树节到了，学校组织我们去山上种(树shu) 。 （92% ）

Arbor Day is here, and the school organizes us to plant trees.

您所预测的字的拼音中是否包含字母 "u" ?

您所预测的字的拼音中是否包含字母 "o" ?

Does the pinyin of your predicted word contain the letter "u"?

Does the pinyin of your predicted word contain the letter "o"?

23. 小红是个懂事的孩子，吃完饭总是抢着帮妈妈洗(碗wan)。（92% ）

Xiaohong is a sensible child, always rushing to help her mother wash dishes.

您所预测的字的拼音中是否包含字母 "n" ?

您所预测的字的拼音中是否包含字母 "i" ?

Does the pinyin of your predicted word contain the letter "n"?

Does the pinyin of your predicted word contain the letter "i"?

24. 现在的女孩子都特别注重自己的形象，出门前都要化(妆zhuang)。（89%）

 Girls nowadays pay special attention to their own image, and before going out they have to put on makeup.

您所预测的字的拼音中是否包含字母 "g" ?

您所预测的字的拼音中是否包含字母 "i" ?

Does the pinyin of your predicted word contain the letter "g"?

Does the pinyin of your predicted word contain the letter "i"?

25. 家里的窗户被打碎了，门锁也被撬了，肯定进了(贼zei)。（84% ）

The windows at home were broken and the door lock was picked, so there must be a thief

您所预测的字的拼音中是否包含字母 "i" ?

您所预测的字的拼音中是否包含字母 "o" ?

Does the pinyin of your predicted word contain the letter "i"?

Does the pinyin of your predicted word contain the letter "o"?

26. 他实在太胖了，真的需要赶紧减(肥fei)。（92%  ）

He is too fat and really needs to lose weight

您所预测的字的拼音中是否包含字母 "i" ?

您所预测的字的拼音中是否包含字母 "a" ?

Does the pinyin of your predicted word contain the letter "i"?

Does the pinyin of your predicted word contain the letter "a"?

27. 为了锻炼身体，我每天吃完晚饭后都去操场跑(步bu)。（92% ）

In order to exercise, every day on the playground after dinner I go for a run.

您所预测的字的拼音中是否包含字母 "u" ?

您所预测的字的拼音中是否包含字母 "n" ?

Does the pinyin of your predicted word contain the letter "u"?

Does the pinyin of your predicted word contain the letter "n"?

28. 最近天气太热了，真期待能下一场大(雨yu)。（92% ）

The weather has been too hot recently. I really look forward to a heavy rain

您所预测的字的拼音中是否包含字母 "y" ?

您所预测的字的拼音中是否包含字母 "n" ?

Does the pinyin of your predicted word contain the letter "y"?

Does the pinyin of your predicted word contain the letter "n"?

29. 春节快要到了，我期待学校赶紧放寒(假jia)。（95% ）

The Spring Festival is coming soon, and I am looking forward to the winter vacation

您所预测的字的拼音中是否包含字母 "a" ?

您所预测的字的拼音中是否包含字母 "e" ?

Does the pinyin of your predicted word contain the letter "a"?

Does the pinyin of your predicted word contain the letter "e"?

30. 这个大钻戒看起来好漂亮，在灯光下闪闪发(光guang)。（100% ）

This big diamond ring looks so beautiful, and under the light it is sparkling

您所预测的字的拼音中是否包含字母 "g" ?

您所预测的字的拼音中是否包含字母 "e" ?

Does the pinyin of your predicted word contain the letter "g"?

Does the pinyin of your predicted word contain the letter "e"?

31. 刚接的开水不要立马就去喝，不然会烫(嘴zui)。（76% ）

Don’t drink the boiled water you just received, otherwise it will burn your mouth

您所预测的字的拼音中是否包含字母 "i" ?

您所预测的字的拼音中是否包含字母 "g" ?

Does the pinyin of your predicted word contain the letter "i"?

Does the pinyin of your predicted word contain the letter "g"?

32. 王刚考了班级倒数第一名，他现在很伤(心xin)。（92% ）

Wang just took the exam and ranked last in the class. He is very sad now

您所预测的字的拼音中是否包含字母 "n" ?

您所预测的字的拼音中是否包含字母 "o" ?

Does the pinyin of your predicted word contain the letter "n"?

Does the pinyin of your predicted word contain the letter "o"?

33. 爷爷退休之后很清闲，每天都去河边钓(鱼yu)。（92% ）

Grandpa has a lot of leisure time after retirement. He goes to the river to going fishing

您所预测的字的拼音中是否包含字母 "y" ?

您所预测的字的拼音中是否包含字母 "a" ?

Does the pinyin of your predicted word contain the letter "y"?

Does the pinyin of your predicted word contain the letter "a"?

34. 今天是开学第一天，老师会给我们发新(书shu)。（86% ）

Today is the first day of school, the teacher will send us a new book

您所预测的字的拼音中是否包含字母 "u" ?

您所预测的字的拼音中是否包含字母 "g" ?

Does the pinyin of your predicted word contain the letter "u"?

Does the pinyin of your predicted word contain the letter "g"?

35. 爸爸给我买的这双鞋太小了，穿起来磨(脚jiao)。（95% ）

The pair of shoes my father bought for me are too small, and they wear out their feet.

您所预测的字的拼音中是否包含字母 "o" ?

您所预测的字的拼音中是否包含字母 "n" ?

Does the pinyin of your predicted word contain the letter "o"?

Does the pinyin of your predicted word contain the letter "n"?

36. 我想把这个西瓜切开，请去厨房给我拿一把(刀dao)。（95% ）

I want to cut this watermelon, please go to the kitchen and get me a (knife)

您所预测的字的拼音中是否包含字母 "o" ?

您所预测的字的拼音中是否包含字母 "i" ?

Does the pinyin of your predicted word contain the letter "o"?

Does the pinyin of your predicted word contain the letter "i"?

37. 我们要节约用电，白天光线充足的时候不要开(灯deng)。（89% ）

We must save electricity, and when there is sufficient light during the day, please do not turn on (lights)

您所预测的字的拼音中是否包含字母 "g" ?

您所预测的字的拼音中是否包含字母 "u" ?

Does the pinyin of your predicted word contain the letter "g"?

Does the pinyin of your predicted word contain the letter "u"?

38. 我们要讲文明守秩序，在食堂打饭的时候不要插(队dui)。（92% ）

We must talk about civility and order, and when eating in the cafeteria don't intervene queuing

您所预测的字的拼音中是否包含字母 "i" ?

您所预测的字的拼音中是否包含字母 "n" ?

Does the pinyin of your predicted word contain the letter "i"?

Does the pinyin of your predicted word contain the letter "n"?

39. 他刚做过手术，医生嘱咐他不要抽烟喝(酒jiu)。（92% ）

He just had an operation, and the doctor told him not to smoke or drink wine

您所预测的字的拼音中是否包含字母 "u" ?

您所预测的字的拼音中是否包含字母 "a" ?

Does the pinyin of your predicted word contain the letter "u"?

Does the pinyin of your predicted word contain the letter "a"?

40. 兰兰和她的男朋友已经相恋8年了，打算明年结(婚hun)。（92% ）

Lanlan and her boyfriend have been in love for 8 years and next year plan to get married

您所预测的字的拼音中是否包含字母 "n" ?

您所预测的字的拼音中是否包含字母 "e" ?

Does the pinyin of your predicted word contain the letter "n"?

Does the pinyin of your predicted word contain the letter "e"?

41. 结婚后夫妻双方要对彼此忠诚，千万不要出(轨gui)。（92% ）

After marriage, the husband and wife should be loyal to each other and never cheat on each other.

您所预测的字的拼音中是否包含字母 "i" ?

您所预测的字的拼音中是否包含字母 "o" ?

Does the pinyin of your predicted word contain the letter "i"?

Does the pinyin of your predicted word contain the letter "o"?

42. 她和男朋友的感情非常地好，从来不吵(架jia)。（86% ）

She has a very good relationship with her boyfriend and they never quarrel

您所预测的字的拼音中是否包含字母 "a" ?

您所预测的字的拼音中是否包含字母 "e" ?

Does the pinyin of your predicted word contain the letter "a"?

Does the pinyin of your predicted word contain the letter "e"?

43. 这个世界上没有无缘无故的爱，也没有无缘无故的(恨hen) 。（89%）

There is no love without reason in this world, and likewise there is no hatred

您所预测的字的拼音中是否包含字母 "n" ?

您所预测的字的拼音中是否包含字母 "o" ?

Does the pinyin of your predicted word contain the letter "n"?

Does the pinyin of your predicted word contain the letter "o"?

44. 我喜欢喝甜的，请在咖啡里帮我多放点(糖tang) 。 （92% ）

I like to drink sweet food. Please put more sugar in my coffee

您所预测的字的拼音中是否包含字母 "g" ?

您所预测的字的拼音中是否包含字母 "u" ?

Does the pinyin of your predicted word contain the letter "g"?

Does the pinyin of your predicted word contain the letter "u"?

45. 我刚下飞机，就看到姑姑在不远处向我招(手shou) 。 （95%）

As soon as I got off the plane, I saw my aunt waving hands.

您所预测的字的拼音中是否包含字母 "u" ?

您所预测的字的拼音中是否包含字母 "i" ?

Does the pinyin of your predicted word contain the letter "u"?

Does the pinyin of your predicted word contain the letter "i"?

46. 我已经闷在家里好几天了，打算待会儿出去透透(气qi) 。 （92% ）

I have been bored at home for several days, and I plan to go out later to get some air

您所预测的字的拼音中是否包含字母 "i" ?

您所预测的字的拼音中是否包含字母 "a" ?

Does the pinyin of your predicted word contain the letter "i"?

Does the pinyin of your predicted word contain the letter "a"?

47. 小丽天生有一副好嗓门，我们都喜欢听他唱(歌ge) 。 （89% ）

Xiaoli is born with a good voice. We all like to listen to him sing songs

您所预测的字的拼音中是否包含字母 "e" ?

您所预测的字的拼音中是否包含字母 "i" ?

Does the pinyin of your predicted word contain the letter "e"?

Does the pinyin of your predicted word contain the letter "i"?

48. 这个女孩身材比例特别好，拥有一双让人羡慕的大长(腿tui) 。 （92%）

This girl has a particularly good figure and has a pair of enviable long legs

您所预测的字的拼音中是否包含字母 "i" ?

您所预测的字的拼音中是否包含字母 "o" ?

 Does the pinyin of your predicted word contain the letter "i"?

Does the pinyin of your predicted word contain the letter "o"?

49. 为了保持口腔健康，最好每天刷两次(牙ya) 。 （92%）

In order to maintain oral health, it is best to brush your teeth

您所预测的字的拼音中是否包含字母 "a" ?

您所预测的字的拼音中是否包含字母 "o" ?

Does the pinyin of your predicted word contain the letter "a"?

Does the pinyin of your predicted word contain the letter "o"?

50. 疫情期间我们要少出门，多通风，勤洗(手shou) 。 （89%）

During the epidemic, we should go out less, ventilate more, and wash hands

您所预测的字的拼音中是否包含字母 "u" ?

您所预测的字的拼音中是否包含字母 "g" ?

Does the pinyin of your predicted word contain the letter "u"?

Does the pinyin of your predicted word contain the letter "g"?

51. 站在一望无际的大草原上，我想去骑(马ma) 。 （89%）

Standing on the endless prairie, I want to ride a horse

您所预测的字的拼音中是否包含字母 "a" ?

您所预测的字的拼音中是否包含字母 "u" ?

Does the pinyin of your predicted word contain the letter "a"?

Does the pinyin of your predicted word contain the letter "u"?

52. 他不能参加比赛了，因为他的腰部受了重(伤shang) 。 （92%）

He can't take part in the competition because he hurt his waist

您所预测的字的拼音中是否包含字母 "g" ?

您所预测的字的拼音中是否包含字母 "o" ?

Does the pinyin of your predicted word contain the letter "g"?

Does the pinyin of your predicted word contain the letter "o"?

53. 这个小女孩多才多艺，不仅会唱歌还会跳(舞wu) 。（89%）

This little girl is versatile. She can not only sing but also dance

您所预测的字的拼音中是否包含字母 "u" ?

您所预测的字的拼音中是否包含字母 "a" ?

Does the pinyin of your predicted word contain the letter "u"?

Does the pinyin of your predicted word contain the letter "a"?

54. 这位德高望重的科学家刚进会场，观众就立马起立鼓(掌zhang) 。（92%）

As soon as this respected scientist entered the venue, the audience immediately stood up and applauded

您所预测的字的拼音中是否包含字母 "g" ?

您所预测的字的拼音中是否包含字母 "i" ?

Does the pinyin of your predicted word contain the letter "g"?

Does the pinyin of your predicted word contain the letter "i"?

55. 最近小偷很多，你走的时候千万不要忘了锁(门men) 。 （92%）

There have been a lot of thieves recently, so when you leave don't forget to lock the door

您所预测的字的拼音中是否包含字母 "n" ?

您所预测的字的拼音中是否包含字母 "o" ?

Does the pinyin of your predicted word contain the letter "n"?

Does the pinyin of your predicted word contain the letter "o"?

56. 虽然我脸上的伤好了，但是却留下了一道(疤ba) 。（89%）

Although the wound on my face healed, there was a (scar)

您所预测的字的拼音中是否包含字母 "a" ?

您所预测的字的拼音中是否包含字母 "g" ?

Does the pinyin of your predicted word contain the letter "a"?

Does the pinyin of your predicted word contain the letter "g"?

57. 五一期间出行人流量大，高速路上很容易堵(车che) 。 (95%)

During the May 1st period, there is a large flow of people, and it is easy to be blocked on the expressway

您所预测的字的拼音中是否包含字母 "e" ?

您所预测的字的拼音中是否包含字母 "a" ?

Does the pinyin of your predicted word contain the letter "e"?

Does the pinyin of your predicted word contain the letter "a"?

58. 他是一名户外运动爱好者，每到周末都要去爬(山shan) 。 (92%)

He is an outdoor sports enthusiast, and every weekend he has to climb (mountain)

您所预测的字的拼音中是否包含字母 "n" ?

您所预测的字的拼音中是否包含字母 "i" ?

Does the pinyin of your predicted word contain the letter "n"?

Does the pinyin of your predicted word contain the letter "i"?

59. 每个周一早上，我们学校都要组织同学们去广场上升国(旗qi) 。 (95%)

Every Monday morning, our school organizes students to go to the square to raise the national flag

您所预测的字的拼音中是否包含字母 "i" ?

您所预测的字的拼音中是否包含字母 "g" ?

 Does the pinyin of your predicted word contain the letter "i"?

Does the pinyin of your predicted word contain the letter "g"?

60. 这个星期我要去美术馆，因为里面展览了很多幅达芬奇的(画hua) 。(95%)

I'm going to the art museum this week, because there are many Leonardo da Vinci's painting

您所预测的字的拼音中是否包含字母 "a" ?

您所预测的字的拼音中是否包含字母 "n" ?

Does the pinyin of your predicted word contain the letter "a"?

Does the pinyin of your predicted word contain the letter "n"?

61. 雪莉是个性格内向的女孩，不喜欢和同学说(话hua) 。 (92%)

Shirley is an introverted girl who doesn't like talking

您所预测的字的拼音中是否包含字母 "a" ?

您所预测的字的拼音中是否包含字母 "i" ?

Does the pinyin of your predicted word contain the letter "a"?

Does the pinyin of your predicted word contain the letter "i"?

62. 马上就要交卷了，我还有两道题没做出来，急得直挠(头tou) 。(84%)

I'm about to hand in the paper, and I still have two questions to answer, so I'm in such a hurry and scratching my head

您所预测的字的拼音中是否包含字母 "u" ?

您所预测的字的拼音中是否包含字母 "a" ?

Does the pinyin of your predicted word contain the letter "u"?

Does the pinyin of your predicted word contain the letter "a"?

63. 赵丽颖是个十足的工作狂，忙起来总是顾不上吃(饭fan) 。 (89%)

Zhao Liying is a complete workaholic, and she always does not have time for food

您所预测的字的拼音中是否包含字母 "n" ?

您所预测的字的拼音中是否包含字母 "i" ?

Does the pinyin of your predicted word contain the letter "n"?

Does the pinyin of your predicted word contain the letter "i"?

64. 范冰冰是个劣迹艺人，因为她曾涉嫌偷(税shui) 。 (81%)

Fan Bingbing is a bad artist because she was once suspected of stealing taxes

您所预测的字的拼音中是否包含字母 "i" ?

您所预测的字的拼音中是否包含字母 "a" ?

Does the pinyin of your predicted word contain the letter "i"?

Does the pinyin of your predicted word contain the letter "a"?

65. 现在的很多明星和网红都在抖音上做直播带(货huo) 。(92%)

Many celebrities and Internet celebrities now make live broadcasts on Tiktok to sell goods.

您所预测的字的拼音中是否包含字母 "o" ?

您所预测的字的拼音中是否包含字母 "g" ?

Does the pinyin of your predicted word contain the letter "o"?

Does the pinyin of your predicted word contain the letter "g"?

66. 赶紧去楼顶把衣服收了，因为天要刮大(风feng) 。 (84%)

Go to the roof of the building quickly to collect your clothes, because there will be wind.

您所预测的字的拼音中是否包含字母 "g" ?

您所预测的字的拼音中是否包含字母 "u" ?

Does the pinyin of your predicted word contain the letter "g"?

Does the pinyin of your predicted word contain the letter "u"?

67. 他的度量实在太小了，因为一件小事就跟我翻(脸lian) 。(81%)

His tolerance is too small, and he would fall out with me over a trivial matter.

您所预测的字的拼音中是否包含字母 "n" ?

您所预测的字的拼音中是否包含字母 "o" ?

Does the pinyin of your predicted word contain the letter "n"?

Does the pinyin of your predicted word contain the letter "o"?

68. 如果你不按规则随便乱停车，就会被罚(款kuan) 。 (76%)

If you park randomly without following the rules, you will be fined

您所预测的字的拼音中是否包含字母 "n" ?

您所预测的字的拼音中是否包含字母 "o" ?

Does the pinyin of your predicted word contain the letter "n"?

Does the pinyin of your predicted word contain the letter "o"?

69. 李白是一个很有才华的人，他的一生作了很多首(诗shi) 。 (92%)

Li Bai was a very talented man and in his life wrote many poems

您所预测的字的拼音中是否包含字母 "i" ?

您所预测的字的拼音中是否包含字母 "g" ?

Does the pinyin of your predicted word contain the letter "i"?

Does the pinyin of your predicted word contain the letter "g"?

70. 春天来了，花丛中有很多蜜蜂在勤劳地采(蜜mi) 。 (86%)

Spring is coming, and there are many bees in the flowers working hard to collect honey

您所预测的字的拼音中是否包含字母 "i" ?

您所预测的字的拼音中是否包含字母 "n" ?

Does the pinyin of your predicted word contain the letter "i"?

Does the pinyin of your predicted word contain the letter "n"?

71. 老婆觉得这个衣服卖的太贵了，她正在和老板砍(价jia) 。 (86%)

My wife thinks this dress is too expensive, and she is negotiating with the boss about the price

您所预测的字的拼音中是否包含字母 "a" ?

您所预测的字的拼音中是否包含字母 "n" ?

Does the pinyin of your predicted word contain the letter "a"?

Does the pinyin of your predicted word contain the letter "n"?

72. 等车的时候要注意保持安全距离，不要越过黄(线xian) 。 (95%)

When waiting for the bus, be sure to keep a safe distance and do not cross the yellow (line)

您所预测的字的拼音中是否包含字母 "n" ?

您所预测的字的拼音中是否包含字母 "u" ?

Does the pinyin of your predicted word contain the letter "n"?

Does the pinyin of your predicted word contain the letter "u"?

73. 他是个数学天才，每次数学考试都能拿满(分fen) 。 (92%)

He is a math genius and on every math test he gets full marks

您所预测的字的拼音中是否包含字母 "n" ?

您所预测的字的拼音中是否包含字母 "a" ?

Does the pinyin of your predicted word contain the letter "n"?

Does the pinyin of your predicted word contain the letter "a"?

74. 昨天我过生日，妈妈给我做了满满的一桌(菜cai) 。 (89%)

It was my birthday yesterday, and my mother cooked a table of dishes

您所预测的字的拼音中是否包含字母 "i" ?

您所预测的字的拼音中是否包含字母 "g" ?

Does the pinyin of your predicted word contain the letter "i"?

Does the pinyin of your predicted word contain the letter "g"?

75. 美国已经有3000万人感染了新冠病毒，今天又有两千人被确(诊zhen) 。 (95%)

Thirty million people in the United States have been infected with the new coronavirus, and today 2,000 more people have been diagnosed

您所预测的字的拼音中是否包含字母 "n" ?

您所预测的字的拼音中是否包含字母 "o" ?

Does the pinyin of your predicted word contain the letter "n"?

Does the pinyin of your predicted word contain the letter "o"?

76. 弟弟参加了学校运动会的跑步项目，并且获得了一等(奖jiang) 。 (92%)

My younger brother participated in the running event of the school sports meeting and won the first prize

您所预测的字的拼音中是否包含字母 "g" ?

您所预测的字的拼音中是否包含字母 "u" ?

Does the pinyin of your predicted word contain the letter "g"?

Does the pinyin of your predicted word contain the letter "u"?

77. 脚踩在雪地上发出咯吱咯吱的声音，身后留下一串清晰的脚(印yin) 。 (89%)

You need to prepare a lot of materials for the final exam. The dean said you can go to his office to make copies

您所预测的字的拼音中是否包含字母 "n" ?

您所预测的字的拼音中是否包含字母 "a" ?

Does the pinyin of your predicted word contain the letter "n"?

Does the pinyin of your predicted word contain the letter "a"?

78. 给患者做手术前，医生都会先对伤口进行消(毒du) 。 (89%)

Before operating on a patient, the doctor will disinfect the wound

您所预测的字的拼音中是否包含字母 "u" ?

您所预测的字的拼音中是否包含字母 "g" ?

Does the pinyin of your predicted word contain the letter "u"?

Does the pinyin of your predicted word contain the letter "g"?

79. 放学后，校长召集所有的老师们去会议室开(会hui) 。(86%)

After school, the principal called all the teachers to the conference room for a (meeting)

您所预测的字的拼音中是否包含字母 "i" ?

您所预测的字的拼音中是否包含字母 "o" ?

Does the pinyin of your predicted word contain the letter "i"?

Does the pinyin of your predicted word contain the letter "o"?

80. 大家都说这场比赛的结果不公平，一定有黑(幕mu) 。 (84%)

Everyone said that the result of this game was unfair and that there must be some evil behind it

您所预测的字的拼音中是否包含字母 "u" ?

您所预测的字的拼音中是否包含字母 "e" ?

Does the pinyin of your predicted word contain the letter "u"?

Does the pinyin of your predicted word contain the letter "e"?

81. 哥哥今年炒股赚了很多钱，所以又买了一套(房fang) 。 (89%)

Brother made a lot of money from stock trading this year, so he bought another house

您所预测的字的拼音中是否包含字母 "g" ?

您所预测的字的拼音中是否包含字母 "e" ?

Does the pinyin of your predicted word contain the letter "g"?

Does the pinyin of your predicted word contain the letter "e"?

82. 你这篇作文文采不错，但是里面有很多错别(字zi) 。 (95%)

Your essay is of good literary quality, but there are many mistaken words.

您所预测的字的拼音中是否包含字母 "i" ?

您所预测的字的拼音中是否包含字母 "a" ?

Does the pinyin of your predicted word contain the letter "i"?

Does the pinyin of your predicted word contain the letter "a"?

83. 哥哥抢走了妹妹的布娃娃，妹妹哭着去找妈妈告(状zhuang) 。 (81% )

The brother took away the sister's doll. The sister cried and went to her mother to complain

您所预测的字的拼音中是否包含字母 "g" ?

您所预测的字的拼音中是否包含字母 "e" ?

Does the pinyin of your predicted word contain the letter "g"?

Does the pinyin of your predicted word contain the letter "e"?

84. 最近公司很忙，老板逼着我们天天加(班ban) 。 (92%)

The company is very busy recently, and every day the boss forces us to work overtime

您所预测的字的拼音中是否包含字母 "n" ?

您所预测的字的拼音中是否包含字母 "u" ?

Does the pinyin of your predicted word contain the letter "n"?

Does the pinyin of your predicted word contain the letter "u"?

85. 这份文件非常重要，你要再三检查千万不能出任何差(错cuo) 。(92%)

This document is very important. You should check it again and again to make sure there are no mistakes

您所预测的字的拼音中是否包含字母 "o" ?

您所预测的字的拼音中是否包含字母 "e" ?

Does the pinyin of your predicted word contain the letter "o"?

Does the pinyin of your predicted word contain the letter "e"?

86. 今天上学你要多穿些衣服，因为天气要降(温wen) 。（84%）

You have to wear more clothes when you go to school today, because the weather is going to drop (temperature)

您所预测的字的拼音中是否包含字母 "n" ?

您所预测的字的拼音中是否包含字母 "o" ?

Does the pinyin of your predicted word contain the letter "n"?

Does the pinyin of your predicted word contain the letter "o"?

87. 公司今年的效益不好，所以老板决定裁(员yuan) 。 （89%）

The company's performance this year is not good, so the boss decided to lay off employees

您所预测的字的拼音中是否包含字母 "n" ?

您所预测的字的拼音中是否包含字母 "e" ?

Does the pinyin of your predicted word contain the letter "n"?

Does the pinyin of your predicted word contain the letter "e"?

88. 对于男人而言，他们都喜欢在手腕上带一只表(表biao) 。（89%）

For men, they all like to wear a watch on their wrist

您所预测的字的拼音中是否包含字母 "o" ?

您所预测的字的拼音中是否包含字母 "g" ?

Does the pinyin of your predicted word contain the letter "o"?

Does the pinyin of your predicted word contain the letter "g"?

89. 我的表姐已经30岁了还没有男朋友，舅舅让她明天去相(亲qin) 。 （95%）

My cousin is 30 years old and has no boyfriend yet. Tomorrow my uncle asked her to go on a blind date

您所预测的字的拼音中是否包含字母 "n" ?

您所预测的字的拼音中是否包含字母 "a" ?

Does the pinyin of your predicted word contain the letter "n"?

Does the pinyin of your predicted word contain the letter "a"?

90. 由于通货膨胀的缘故，人民币在不断地贬(值zhi)。（92%）

Due to inflation, the renminbi is constantly depreciating

您所预测的字的拼音中是否包含字母 "i" ?

您所预测的字的拼音中是否包含字母 "u" ?

Does the pinyin of your predicted word contain the letter "i"?

Does the pinyin of your predicted word contain the letter "u"?

91. 这个女孩子心眼儿特别小，动不动就生(气qi) 。 （89%）

This girl is very petty and gets angry easily.

您所预测的字的拼音中是否包含字母 "i" ?

您所预测的字的拼音中是否包含字母 "a" ?

Does the pinyin of your predicted word contain the letter "i"?

Does the pinyin of your predicted word contain the letter "a"?

92. 他第一次一个人来到这个陌生的城市，晚上特别想爸妈想回(家jia) 。 （89%）

It was the first time he came to this strange city alone, and at night he especially missed his parents and wanted to go home

您所预测的字的拼音中是否包含字母 "a" ?

您所预测的字的拼音中是否包含字母 "o" ?

Does the pinyin of your predicted word contain the letter "a"?

Does the pinyin of your predicted word contain the letter "o"?

93. 他没有本事但是又死要面子，所以总在朋友面前吹(牛niu) 。 （86%）

He has no skills but wants to save face, so he always brags (niu) in front of his friends.

您所预测的字的拼音中是否包含字母 "u" ?

您所预测的字的拼音中是否包含字母 "e" ?

Does the pinyin of your predicted word contain the letter "u"?

Does the pinyin of your predicted word contain the letter "e"?

94. 如果你没有钱全款买房子也没关系，可以先付个首(付fu) 。 （89%）

If you don’t have the money to buy a house in full, it doesn’t matter. You can pay a down payment

您所预测的字的拼音中是否包含字母 "u" ?

您所预测的字的拼音中是否包含字母 "g" ?

Does the pinyin of your predicted word contain the letter "u"?

Does the pinyin of your predicted word contain the letter "g"?

95. 这个小女孩从小被父母娇生惯养，从来没有吃过(苦ku) 。（78%）

This little girl was spoiled and spoiled by her parents since she was a child, and she has never experienced hardships.

您所预测的字的拼音中是否包含字母 "u" ?

您所预测的字的拼音中是否包含字母 "o" ?

Does the pinyin of your predicted word contain the letter "u"?

Does the pinyin of your predicted word contain the letter "o"?

96. 这个好消息来得太突然，感觉就像做了一场(梦meng) 。（95%）

This good news came so suddenly, it felt like a dream

您所预测的字的拼音中是否包含字母 "g" ?

您所预测的字的拼音中是否包含字母 "u" ?

Does the pinyin of your predicted word contain the letter "g"?

Does the pinyin of your predicted word contain the letter "u"?

97. 我最近心情很不好，连着好几天晚上都失(眠mian) 。 （95%）

I have been in a bad mood recently and have been unable to sleep for several days in a row

您所预测的字的拼音中是否包含字母 "n" ?

您所预测的字的拼音中是否包含字母 "g" ?

Does the pinyin of your predicted word contain the letter "n"?

Does the pinyin of your predicted word contain the letter "g"?

98. 她嫁给了一个离过婚带孩子的男人，所以年纪轻轻就当了后(妈ma) 。 （86%）

She married a divorced man with children, so she became a mother at a young age.

您所预测的字的拼音中是否包含字母 "a" ?

您所预测的字的拼音中是否包含字母 "i" ?

Does the pinyin of your predicted word contain the letter "a"?

Does the pinyin of your predicted word contain the letter "i"?

99. 弟弟吃饭的时候太调皮，一不小心就打碎了一只(碗wan) 。 （81%）

My younger brother was too naughty when eating and accidentally broke a bowl

您所预测的字的拼音中是否包含字母 "n" ?

您所预测的字的拼音中是否包含字母 "o" ?

Does the pinyin of your predicted word contain the letter "n"?

Does the pinyin of your predicted word contain the letter "o"?

100. 我们做事情要脚踏实地勤勤恳恳，因为成功没有捷(径jing) 。 （86%）

We must be down-to-earth and diligent in doing things, because there is no shortcut

您所预测的字的拼音中是否包含字母 "g" ?

您所预测的字的拼音中是否包含字母 "a" ?

Does the pinyin of your predicted word contain the letter "g"?

Does the pinyin of your predicted word contain the letter "a"?

101. 儿子的脚长大了，我要带他去买双新(鞋xie) 。 （92%）

My son’s feet have grown up. I want to take him to buy a new pair of shoes

您所预测的字的拼音中是否包含字母 "e" ?

您所预测的字的拼音中是否包含字母 "u" ?

Does the pinyin of your predicted word contain the letter "e"?

Does the pinyin of your predicted word contain the letter "u"?

102. 小刚新交了一个女朋友，他们俩这周末要去公园约(会hui) 。 （92%）

Xiaogang has a new girlfriend. They are going to the park for a date

您所预测的字的拼音中是否包含字母 "i" ?

您所预测的字的拼音中是否包含字母 "e" ?

Does the pinyin of your predicted word contain the letter "i"?

Does the pinyin of your predicted word contain the letter "e"?

103. 袁华是一名高三的学生，再有两个多月就要参加高(考kao) 。 （95%）

Yuan Hua is a senior high school student and will take the high school entrance exam in more than two months.

您所预测的字的拼音中是否包含字母 "o" ?

您所预测的字的拼音中是否包含字母 "u" ?

Does the pinyin of your predicted word contain the letter "o"?

Does the pinyin of your predicted word contain the letter "u"?

104. 为了降低新冠病毒传播风险，国家提倡我们要尽快打疫(苗miao) 。 （97%）

In order to reduce the risk of the spread of the new coronavirus, the state advocates that we should vaccinate (miao) as soon as possible

您所预测的字的拼音中是否包含字母 "o" ?

您所预测的字的拼音中是否包含字母 "e" ?

Does the pinyin of your predicted word contain the letter "o"?

Does the pinyin of your predicted word contain the letter "e"?

105. 苹果手机像素非常高，我非常喜欢用它来拍(照zhao) 。 （95%）

Apple mobile phones have very high pixels and I like to use them to take photos

您所预测的字的拼音中是否包含字母 "o" ?

您所预测的字的拼音中是否包含字母 "n" ?

Does the pinyin of your predicted word contain the letter "o"?

Does the pinyin of your predicted word contain the letter "n"?

106. 明星吴越不喜欢化妆，她每次出席活动都是素(颜yan) 。 （95%）

Wu Yue doesn't like makeup, she always wears plain (Yan yan) every time she attends an event

您所预测的字的拼音中是否包含字母 "n" ?

您所预测的字的拼音中是否包含字母 "u" ?

Does the pinyin of your predicted word contain the letter "n"?

Does the pinyin of your predicted word contain the letter "u"?

107. 沈腾曾经又瘦又年轻，但是最终还是难逃中年发(福fu) 。 （76%）

Shen Teng was once thin and young, but in the end he couldn’t escape the fate of becoming fat.

您所预测的字的拼音中是否包含字母 "u" ?

您所预测的字的拼音中是否包含字母 "a" ?

Does the pinyin of your predicted word contain the letter "u"?

Does the pinyin of your predicted word contain the letter "a"?

108. 中国好声音在开始正式比赛之前，需要在全国范围内进行海(选xuan) 。（95%）

Before starting the official competition, The Voice of China needs to carry out the first-round of competitions across the country

您所预测的字的拼音中是否包含字母 "n" ?

您所预测的字的拼音中是否包含字母 "o" ?

Does the pinyin of your predicted word contain the letter "n"?

Does the pinyin of your predicted word contain the letter "o"?

109. 很多学生毕业之后没有找工作，而是选择了自主创(业ye) 。 （92%）

Many students did not find a job after graduation, but chose to start their own business

您所预测的字的拼音中是否包含字母 "e" ?

您所预测的字的拼音中是否包含字母 "a" ?

Does the pinyin of your predicted word contain the letter "e"?

Does the pinyin of your predicted word contain the letter "a"?

110. 玲玲非常喜欢宠物，家里边养了很多猫和(狗gou) 。 （95%）

Lingling likes pets very much. There are many cats and gou in the house

您所预测的字的拼音中是否包含字母 "u" ?

您所预测的字的拼音中是否包含字母 "i" ?

Does the pinyin of your predicted word contain the letter "u"?

Does the pinyin of your predicted word contain the letter "i"?

111. 现在互联网行业竞争激烈，如果你不好好干会立马失(业ye) 。（92% ）

The competition in the Internet industry is fierce now, if you don’t work hard, you will lose your job

您所预测的字的拼音中是否包含字母 "e" ?

您所预测的字的拼音中是否包含字母 "o" ?

Does the pinyin of your predicted word contain the letter "e"?

Does the pinyin of your predicted word contain the letter "o"?

112. 我的儿子什么都喜欢吃，吃饭从来不挑(食shi) 。 （89%）

My son likes to eat everything, and he is never picky about food

您所预测的字的拼音中是否包含字母 "i" ?

您所预测的字的拼音中是否包含字母 "a" ?

Does the pinyin of your predicted word contain the letter "i"?

Does the pinyin of your predicted word contain the letter "a"?

113. 作为一名职场上的打工人，我们最渴望的就是升职加(薪xin) 。（86%）

As a worker in the workplace, what we desire most is a promotion and a salary increase

您所预测的字的拼音中是否包含字母 "n"

您所预测的字的拼音中是否包含字母 "e" ?

Does the pinyin of your predicted word contain the letter "n"?

Does the pinyin of your predicted word contain the letter "e"?

114. 山西煤矿发生瓦斯爆炸事故，8名伤者正在医院被抢(救jiu) 。 （86%）

A gas explosion accident occurred in a coal mine in Shanxi, and in the hospital eight injured people were rescued

您所预测的字的拼音中是否包含字母 "u" ?

您所预测的字的拼音中是否包含字母 "a" ?

Does the pinyin of your predicted word contain the letter "u"?

Does the pinyin of your predicted word contain the letter "a"?

115. 这个女人插足了别人的婚姻，成为了一名人人唾骂的小(三san) 。（89%）

This woman meddled in other people’s marriages and became a mistress.

您所预测的字的拼音中是否包含字母 "n" ?

您所预测的字的拼音中是否包含字母 "i" ?

Does the pinyin of your predicted word contain the letter "n"?

Does the pinyin of your predicted word contain the letter "i"?

116. 他现在的妻子是二婚，并不是他的原(配pei) 。（84%）

His current wife is a second marriage, not his first wife.

您所预测的字的拼音中是否包含字母 "i" ?

您所预测的字的拼音中是否包含字母 "g" ?

Does the pinyin of your predicted word contain the letter "i"?

Does the pinyin of your predicted word contain the letter "g"?

117. 现在医美技术越来越发达，很多想要变美的女生选择去整(容rong) 。（89%）

Nowadays, medical and aesthetic technology is more and more developed, and many girls who want to look beautiful choose to have plastic surgery

您所预测的字的拼音中是否包含字母 "g" ?

您所预测的字的拼音中是否包含字母 "u" ?

Does the pinyin of your predicted word contain the letter "g"?

Does the pinyin of your predicted word contain the letter "u"?

118. 章泽天嫁给了京东总裁刘强东，成为了一名豪门阔(太tai)。（81%）

Zhang Zetian married Liu Qiangdong, the president of Jingdong, and became a rich married woman.

您所预测的字的拼音中是否包含字母 "i" ?

您所预测的字的拼音中是否包含字母 "g" ?

Does the pinyin of your predicted word contain the letter "i"?

Does the pinyin of your predicted word contain the letter "g"?

119. 每天放学后，王刚都要去操场找同学们踢足(球qiu) 。（95%）

Every day after school, Wang Gang goes to the playground to play football with his classmates

您所预测的字的拼音中是否包含字母 "u" ?

您所预测的字的拼音中是否包含字母 "o" ?

Does the pinyin of your predicted word contain the letter "u"?

Does the pinyin of your predicted word contain the letter "o"?

120. 按照新的法律规定，男性需要工作到65岁才能退(休xiu) 。 （89%）

According to the new law, men need to work until they are 65 years old before they can retire

您所预测的字的拼音中是否包含字母 "u" ?

您所预测的字的拼音中是否包含字母 "e" ?

Does the pinyin of your predicted word contain the letter "u"?

Does the pinyin of your predicted word contain the letter "e"?

**Appendix B**

**Stimuli for Experiment 2**

All 96 pairs of sentence contexts from Experiment 1 are listed below with the first one in each pair being more predictable and the second one being less predictable. The picture number corresponding to the target word in each pair is shown at the end of each sentence.

1. 小明拨打了120，没过多久就驶来了一辆(救护车 jiu hu che) 。 (96.88%)

Xiao Ming dialed 120, and not long after, arrived an ambulance.

今天不知道发生了什么事，这里停了很多辆(救护车jiu hu che) 。 (2.94%)

I don't know what happened here today, because there are many ambulances.

2. 在中国古代，商人做生意算帐都是用（算盘 suan pan）。（88.24%）

To perform mathematical function, businessmen in ancient China usually use abacus.

如果这些算术题不好算，你可以使用(算盘 suan pan) 。（0%）

If these arithmetic problems are difficult to solve, you can use an abacus.

3. 在上海浦东机场，平均每天起飞1000多架(飞机 fei ji )。（93.75%）

At Shanghai Pudong Airport, on average every day there are more than 1,000 airplanes.

这些恐怖分子胆子太大了，竟然试图劫持(飞机 fei ji) 。（2.94%）

These terrorists are very daring; they attempted to hijack an airplane.

4. 小姑娘的脸红扑扑的，看起来像一个熟透了的(苹果 ping guo ) 。（91.12%）

The little girl's face was rosy, looking like a ripe apple.

昨天我和妈妈去姑姑家做客，给他们带了一箱(苹果 ping guo) 。（9.09%）

Yesterday, my mom and I visited my aunt's house and brought a box of apples.

5. 陈雨菲是一名羽毛球运动员，每天都会练习打（羽毛球 yu mao qiu ）。（88.24%）

Chen Yufei is a badminton player and every day she practices playing badminton.

这家公司研发出了一批专供运动员比赛使用的(羽毛球 yu mao qiu)。 (0.00%)

To make athletes, this company has developed a batch of badminton.

6. 求婚现场，这个男生精心布置了许多只五颜六色的 （气球 qi qiu）。（90.91%）

At the marriage proposal scene, this young man carefully arranged many colourful balloons.

元旦放假期间，大街上很热闹，有一个老爷爷在卖（气球 qi qiu）。（4.55%）

During the New Year's holiday, the streets were lively, and an old man was selling balloons.

7. 外婆年纪大了，苹果咬不动，所以我给她剥了一根（香蕉 xiang jiao）。（91.18%）

Grandma is elderly and can't bite into apples, so I peeled her a banana.

我去医院看望姥姥，给她买了许多她最爱吃的(香蕉 xiang jiao) 。（0%）

I went to the hospital to visit my grandmother and bought her many bananas.

8. 和姚明一样，他最擅长的运动也是打（篮球 lan qiu ）。（100%）

Like Yao Ming, his favorite sport is playing basketball.

今天在商场里，爸爸给弟弟买了一个（篮球 lan qiu）。（4.55%）

Today, at the mall, Dad bought my little brother a basketball.

9. 这间卧室太小了只够一个人住，所以只放了一张单人（床 chuang）。（100.00%）

This bedroom is too small for more than one person, so there's only a single bed.

刚才我下楼倒垃圾，看到小区的垃圾堆旁边放了一张（床 chuang）。（13.64%）

When I went downstairs to take out the trash, next to the garbage pile I saw a bed.

10. 如果你想更清楚地看远处的东西，就需要使用（望远镜wang yuan jing）。 （90.63%）

If you want to see distant things more clearly, you need to use binoculars.

他沉默地望着天空，时不时地举起手中的（望远镜wang yuan jing）。 （0.00%）

He silently gazed at the sky, occasionally raising the binoculars.

11. 请你不要再没事找事了，你这完全就是鸡蛋里挑（骨头 gu tou）。（93.75%）

Please stop looking for trouble when there's none, it is like you're picking bones out of an egg.

老人惊奇地发现，山上的破庙里面居然有几根（骨头 gu tou）。（0.00%）

The elderly person was surprised to find that in the temple on the mountain there were a few bones.

12. 图书馆新进了一批书但是没地方摆放，所以需要买一些（书架 shu jia）。（90.24%）

The library has received a new batch of books, but there's no place to put them, so they need to buy some bookshelves.

刚拖过的地非常滑，所以走路的时候要小心，不要撞倒旁边的(书架 shu jia) 。（0%）

The recently cleaned floor is very slippery, so you need to be careful while walking not to bump into the bookshelves.

13. 小红是个懂事的孩子，吃完饭总是抢着帮妈妈洗（碗 wan）。（90.63%）

Xiao Hong is a sensible child; after every meal, she always eagerly helps her mom wash the dishes.

我和妹妹在客厅跑着玩，不小心打碎了桌子上的（碗 wan）。（0.00%）

When my sister and I were running and playing in the living room, we accidentally broke the dishes.

14. 这个人非常地讲义气，对朋友总是两肋插(刀 dao) 。（97.56%）

This person is very loyal and always stands by his friends.

列车安检员告诉我们，火车上不允许带(刀 dao) 。（6.25%）

The train security officer told us that knives are not allowed on the train.

15. 春天百花盛开，花丛中飞舞着许多美丽的（蝴蝶 hu die）。（100%）

In spring, flowers bloom abundantly, and above the flowers there are many flying butterflies.

她的手非常灵巧，能够绣出各种图案，比如（蝴蝶 hu die）。（0.00%）

Her hands are very skillful and can embroider various patterns, like butterflies.

16. 今天是妈妈的生日，我去蛋糕店给她买了一个生日（蛋糕 dan gao）。（100.00%）

Today is my mom's birthday, so I went to the cake shop to buy her a birthday cake.

妹妹每次从这家小店路过，都会趴在橱窗上看里面的（蛋糕 dan gao）。（4.55%）

Every time my sister passes by this small shop, she leans against the window to look at the cakes.

17. 我不知道今天是什么日子，因为我忘了看（日历 ri li）。（93.75%）

I don't know what day it is today because I forgot to check the calendar.

我的办公桌上摆放了很多物品，其中一个是（日历 ri li）。（0.00%）

There are many items on my office desk, and one of them is a calendar.

18. 新疆有许多我喜欢的动物，尤其是被称为“沙漠之舟”的（骆驼 luo tuo）。（94.12%）

Xinjiang has many animals I like, especially the camels.

这个小宝宝咯咯咯地笑起来，因为他看到不远处有一只健壮的(骆驼 luo tuo) （0.00%）

The little baby giggled because he saw a big camel.

19. 人们经常把老师比喻成为燃烧自己照亮别人的（蜡烛 la zhu）。（90.63%）

People often compare teachers to candles.

警察调查后发现这场大火的罪魁祸首竟然是一支（蜡烛 la zhu）。（9.09%）

After investigation, the police found that the culprit of the fire turned out to be a candle.

20. 他很怕老师，每次见到老师就像老鼠见到（猫 mao）。(96.88%)

He is very afraid of the teacher. Every time he sees the teacher, he is like a mouse seeing a cat.

幼儿园老师正在认真地教小朋友画一只（猫 mao）。(4.55%)

The kindergarten teacher is seriously teaching the children to draw a cat.

21. 他不是什么盖世英雄，而是一个滑稽可笑的跳梁(小丑 xiao chou ) 。(80.49%)

He is not a hero of the world, but a ridiculous jumping beam clown.

为了逗全家人开心，爸爸把自己乔装打扮成了一个(小丑 xiao chou) 。(25%)

To amuse the whole family, dad disguised himself as a clown.

22. 我今天早上起床后没有梳头发，因为我找不到(梳子 shu zi) 。(93.75%)

I didn’t comb my hair after getting up this morning because I couldn’t find comb.

淘气的妹妹偷偷溜进了我的房间，拿走了我的(梳子 shu zi) 。(0.00%)

My naughty sister sneaked into my room and took my comb.

23. 为了辨别方向，轮船在海上航行的时候需要用到（指南针 zhi nan zhen）。 (81.25%)

In order to identify the direction at sea, the ship needs to use compass.

我的爸爸是个海员，所以他很喜欢摆弄桌子上的（指南针 zhi nan zhen）。 (6.25%)

My dad is a sailor so he likes to fiddle with the compass.

24. 他在互联网公司上班，每个人的桌子上都放着一台（电脑 dian nao）。(93.75%)

He works in an Internet company, and on each table there is a computer.

妈妈叫他去吃饭，可他却像没听见一样继续玩（电脑 dian nao）。(4.55%)

His mother asked him to go to dinner, but he continued to play computer.

25. 星期天，我和爸爸去玉米地里帮奶奶掰（玉米 yu mi）。(85.29%)

On Sunday, my father and I went to the cornfield to help grandma break corn.

我去奶奶家玩，看到她的屋檐下挂了许多（玉米 yu mi）。(4.55%)

I went to my grandma’s house to play and under her roof I saw a lot of corn.

26. 他的办公室有一套专门用来招待客人坐的软软的（沙发 sha fa）。(90.63%)

To make guests more comfortable, in his office there is a soft sofa.

下个月我家要搬进新房，打算重新添置一套（沙发 sha fa）。(11.76%)

My family is moving into a new house next month and plans to buy a new set sofa.

27. 他勤勤恳恳，老老实实工作，就像一头甘愿奉献的老黄（牛 niu）。(95.45%)

He works diligently and honestly, just like a willing and dedicated cow.

她虽然看起来很成熟，但是实际上只比我小一岁，她的属相是（牛 niu）。(3.13%)

Although she looks very mature, she is actually only one year younger than me. Her zodiac sign is Ox.

28. 女孩子在家要注意保护隐私，一到晚上就要拉上房间的（窗帘 chuang lian）。(93.75%)

Girls should pay attention to protecting privacy at home, and at night should close the curtains.

我奶奶是一个裁缝，她把我给她买的一块布改成了一个（窗帘 chuang lian）(2.94%)

My grandma is a seamstress and she turned a piece of cloth I bought her into a curtain.

29. 男朋友送了她一枚戒指，上面镶嵌着一颗闪闪发光的（钻石 zuan shi）。(94.12%)

Her boyfriend gave her a ring with a sparkling diamond.

小偷趁主人不在家入室盗窃，偷走了放在保险柜里的（钻石 zuan shi）。(0%)

The thief broke into the house while the owner was not at home and stole the diamond.

30. 在两亿年前的侏罗纪时代，地球上的统治者是（恐龙 kong long）。(100.00%)

During the Jurassic period 200 million years ago, the rulers of the earth were dinosaurs.

让我惊讶的是，这部电影的主角竟然是一只（恐龙 kong long）。(0.00%)

To my surprise, the protagonist of this movie turned out to be a dinosaur.

31. 他从医学院毕业之后进入医院成为了一名外科（医生 yi sheng）。(94.12%)

After graduating from medical school, he entered the hospital and became a surgeon doctor.

他看起来其貌不扬，谁也没想到居然是一名（医生 yi sheng）。(0%)

He looked unattractive, no one expected that he was actually a doctor.

32. 玲玲非常喜欢宠物，家里边养了很多猫和（狗 gou）。(93.75%)

Lingling likes pets very much, and in her house she has many cats and dogs.

这个车主因为车速过快，撞倒了邻居家的（狗 gou）。(8.82%)

Because the driver was driving too fast, he knocked down his neighbor's dog.

33. 妈妈告诉我进别人房间之前，一定要记得先敲（门 men）。(100.00%)

My mother told me that before entering someone else’s room, I must remember to knock door.

我的爷爷是一个木匠，他现在正在帮客户打造(门 men) 。(0%)

My grandfather is a carpenter, and he is now helping customers build doors.

34. 宿舍里实在太吵了，他只能用耳塞塞住（耳朵 er duo）。(100%)

It was so noisy in the dormitory that he could only plug (his ears) with earplugs.

爷爷是一名抗战老兵，在战场上被炸伤了一只（耳朵 er duo）。(0.00%)

Grandpa is a veteran of the Anti-Japanese War and during the war there was a blast injury of his ears.

35. 双方力量悬殊过大，就好像蚂蚁和（大象 da xiang）。(91.18%)

The disparity in power between the two sides is too great, just like an ant and an elephant.

我和弟弟每次去动物园，都要去看（大象 da xiang）。(4.55%)

Every time my brother and I go to the zoo, we have to go see elephant.

36. 小凯考试考砸了，没精打采的，蔫的就像一个霜打的（茄子 qie zi）。(90.63%)

Xiao Kai failed in the exam, listless, and limp like a beaten eggplant eggplant.

今天菜市场有很多人，原来是新上市了一批新鲜的（茄子 qie zi）。(0%)

There are many people in the vegetable market today. It turns out that there is a new batch of fresh eggplant.

37. 爷爷写完信后， 把它小心折叠放入（信封 xin feng）。(88.24%)

After grandpa finished writing the letter, he carefully folded it and put it into the envelope.

在幼儿园里老师教小朋友用纸制作（信封 xin feng）。(0%)

In kindergarten, the teacher teaches children how to use paper to make envelope.

38. 她有一张鹅蛋脸和一双水汪汪的大（眼睛 yan jing）。(100%)

She has an oval face and a pair of watery big eyes.

我不得不承认，这是我见过的最漂亮的一双（眼睛 yan jing）。(13.64%)

I have to admit, this is the most beautiful pair of eyes.

39. 孔雀开屏的时候之所以美丽，是因为它有一身五颜六色的（羽毛 yu mao）。	(93.75%)

The reason why the peacock is beautiful when it opens its tail is because it has colourful feathers.

对于名人来说，一定不要做违反道德的事情，要爱惜自己的（羽毛 yu mao）。(11.76%)

For celebrities, they must not do anything that violates morality and cherish their own feathers.

40. 爷爷退休之后很清闲，每天都去河边钓（鱼 yu）。(97.06%)

Grandpa has a lot of leisure time after retirement. He goes to the river to catch fish.

厨房里传出阵阵香味，原来是妈妈在做红烧（鱼 yu）。(3.13%)

The smell came from the kitchen. It turned out to be my mother cooking braised fish.

41. 这个老板欺人太甚，工人们愤怒地握紧了（拳头 quan tou）。(96.88%)

The boss bullied people so much that the workers clenched their fists in anger.

我快速闪到一边，终于躲过了朝我打过来的(拳头 quan tou) 。(5.88%)

I quickly moved aside and finally dodged the fist.

42. 这种做法非常愚蠢，完全是搬起石头砸自己的(脚 jiao) 。(100%)

This approach is very stupid. It is completely shooting oneself in the foot.

今天他骑摩托车摔倒了，擦破了自己的(脚 jiao) 。(0%)

Today he fell while riding a motorcycle and scratched his foot.

43. 地球上现存的最高、脖子最长的动物就是（长颈鹿 chang jing lu）。(100%)

The tallest and longest-necked animal on earth is giraffe.

南京红山动物园今年从非洲引进了几只（长颈鹿 chang jing lu）。(2.94%)

This year from Africa, Nanjing Hongshan Zoo introduced several giraffes.

44. 现在很多学生都是高度近视，每天都要带（眼镜 yan jing）。(94.12%)

Many students nowadays are highly myopic and have to wear glasses.

出门时候因为太匆忙，我忘了带自己的（眼镜 yan jing）。(0%)

I was in such a hurry when I went out that I forgot to bring my own glasses.

45. 我们人类现在居住的星球叫（地球di qiu）。(100.00%)

The planet we humans live on now is called Earth.

我们要像善待自己一样去善待（地球di qiu）。(0.00%)

We need to be try to be kind to the Earth.

46. 今天下雪了，他的手冻得通红，因为他没有戴（手套 shou tao）。(100.00%)

It snowed today and his hands were red from the cold because he didn’t wear gloves.

我实在是拗不过妈妈，所以只好硬着头皮戴上（手套 shou tao）。(0.00%)

I really couldn't resist my mother, so I had to bite the bullet and put on gloves.

47. 我们都知道，葡萄酒的原材料是（葡萄 pu tao）。(95.45%)

We all know that the raw material of wine is grapes.

和哥哥一样，我们俩最讨厌的水果都是（葡萄 pu tao）。(0.00%)

Like my brother, the fruit we both hate the most is grapes.

48. 这对小夫妻很恩爱，每次出门老公都会牵着老婆的(手 shou) 。(94.12%)

This young couple is very loving, and every time they go outside, the husband will hold his wife’s hand.

她尴尬地笑了笑，一言不发地盯着母亲的(手 shou) 。(13.64%)

She smiled awkwardly and without saying anything, she just stared at her mother's hand.

49. 在图书馆要保持安静，想听歌曲必须要戴上（耳机 er ji）。(100%)

Keep quiet in the library and if you want to listen to songs, please wear headphones.

男友生日的时候，我送了他一副价格昂贵的（耳机 er ji）。(0.00%)

On my boyfriend’s birthday, I gave him an expensive pair of headphones.

50. 你已经铸成大错，千万不可一错再错，请赶紧悬崖勒（马 ma）。(100%)

You have already made a big mistake. You must not make the same mistake again and again. Please step back from the cliff horse.

这是我第一次来到大草原，以前从来没见过这么多的（马 ma）。(12.50%)

This is my first time coming to the prairie. I have never seen so many horses.

51. 具有很强跳跃能力并且刚出生会呆在母亲胸前口袋里的动物是（袋鼠 dai shu）。(100.00%)

The animal that has strong jumping ability and stays in the mother’s breast pocket when it is born is kangaroo.

在自然界有很多四肢敏捷、跳跃能力很强的动物，比如（袋鼠 dai shu）。(18.75%)

In nature, there are many animals with agile limbs and strong jumping abilities, such as kangaroos.

52. 小亮到家了却进不去家门，因为他忘了带（钥匙 yao shi）。（100%）

Xiao Liang got home but couldn’t get in because he forgot to bring key.

女生的包包真是万能的，能放各种东西比如（钥匙 yao shi）。（2.94%）

Girls’ bags are really versatile, they can hold all kinds of things such as keys.

53. 我姐姐说自己既能上得了厅堂，也能下得了（厨房 chu fang）。(97.06%)

My sister said that she can both go to the hall and go to the kitchen.

刚一进门，叔叔就热情地领我去看他们家的（厨房 chu fang）。(0%)

As soon as I walked in, my uncle enthusiastically showed me their kitchen.

54. 今天外面的风很大，非常适合去公园放（风筝 feng zheng）。(100%)

The wind outside is very strong today, which is very suitable for flying kite.

妹妹今天在公园里捡到了一个没人要的（风筝 feng zheng）。(2.94%)

My sister picked up an unwanted kite in the park.

55. 草原上飞奔过来一群被称作“草原之王”、正在愤怒咆哮的（狮子 shi zi）。(81%)

Over the grassland, came over a group of roaring lions.

邻居家爷爷擅长剪纸，不一会儿他就剪了一只栩栩如生的（狮子 shi zi）。(0.00%)

The neighbor’s grandfather is good at paper-cutting, and soon he cut a lifelike lion.

56. 为了防止在山上迷路，你们一定要带一张（地图 di tu）。(100%)

In order to prevent getting lost on the mountain, you must bring a map.

爸爸是一名工程师，他在工作中经常需要用到（地图 di tu）。(0%)

Dad is an engineer, so in his work he often needs to use map.

57. 夏季最能消暑解渴的水果就是又大又圆的无籽(西瓜 xi gua) 。(96.88%)

The fruit that best relieves summer heat and quenches thirst is the big, round seedless watermelon.

弟弟放学一回到家，就迫不及待啃起了桌子上的（西瓜 xi gua）。(2.94%)

As soon as my younger brother came home from school, he couldn't wait to eat the watermelon.

58. 传说中上半身是美女，下半身是鱼尾的生物叫（美人鱼 mei ren yu）。（88%）

According to legend, the creature whose upper body is a beautiful woman and whose lower body is a fish tail is called mermaid.

小时候，我最大的梦想就是变成海里的一条（美人鱼 mei ren yu）。（3.13%）

When I was a child, my biggest dream was to become a mermaid.

59. 湖面上没有一丝风，水面非常平静，像一面（镜子 jing zi）。（100%）

There is no wind on the lake, and the water is very calm, like a mirror.

家里的小猫咪太调皮了，打碎了桌子上的（镜子 jing zi）。（2.94%）

The kitten at home was too naughty and broke the mirror on the table.

60. 这个男明星晚年时看破红尘，出家当了（和尚 he shang）。（97.06%）

In his later years, this male star saw through the world of mortals and became a monk.

今天和妈妈逛街的时候我居然看到了两名（和尚 he shang）。（0.00%）

Today when I was shopping with my mother, I actually saw two monks.

61. 在西游记中，花果山上一共有四万多只调皮可爱的小（猴子 hou zi）。（100%）

In Journey to the West, on Huaguo Mountain there are more than 40,000 naughty and cute little monkeys.

这个男孩总是喜欢乱翻别人的东西，就像一只讨人嫌的（猴子 hou zi）。（4.55%）

This boy always likes to rummage through other people's things, like an annoying monkey.

62. 过去农村养猫不是为了当宠物，而是为了让猫来捉（老鼠 lao shu）。（97.01%）

In the past, cats were kept in rural areas not for pets, but for cats to catch mouse.

我们正在吃火锅，突然锅里面掉进去一只(老鼠 lao shu) 。（0.00%）

We were eating hot pot, suddenly in the pot there came a mouse.

63. 我们操作电脑一般都是通过键盘和（鼠标 shu biao）。（100%）

We usually operate computers through keyboard and mouse.

他想打开电脑工作，却怎么也找不到（鼠标 shu biao）。（14.71%）

He wanted to open the computer to work, but he couldn't find the mouse.

64. 他嗅觉高度灵敏，因为他有一个非常灵敏的（鼻子 bi zi）。（95.45%）

He has a highly sensitive sense of smell because he has a very sensitive nose.

赛场上，穿蓝色短裤的拳击运动员猛击对手的（鼻子 bi zi）。（0.00%）

In the ring, a boxer in blue shorts punches his opponent in the nose.

65. 在医院里面，有着很多穿着白大褂的医生和（护士 hu shi）。（93.75%）

In the hospital, there are many doctors and nurses.

这个女孩子说，自己长大后想要成为一名 （护士 hu shi）。（5.88%）

This girl said when she grew up she wanted to be a nurse.

66. 我们现代人写字用钢笔或者铅笔，而古代人写字用（毛笔 mao bi）。（91.18%）

We modern people use pens or pencils to write, while ancient people used brushes.

今天晚上我偶然发现，爷爷的书房里珍藏了很多（毛笔 mao bi）。（4.55%）

Tonight I accidentally discovered that my grandfather has a lot of brushes.

67. 大家都知道，我们国家的国宝动物是大（熊猫 xiong mao）。（100%）

As we all know, the national treasure animal of our country is the giant panda.

我长这么大从来没去过动物园，也没见过（熊猫 xiong mao）。（13.64%）

I have never been to a zoo or seen a panda.

68. 他们两个兴趣相投，形影不离，关系好到可以穿一条 (裤子 ku zi) 。（97.06%）

The two of them have similar interests and are inseparable. Their relationship is so good that they can wear a pair of pants.

上个月姐姐从国外出差回来，送了我一条昂贵的(裤子 ku zi) 。（0.00%）

Last month, my sister came back from a business trip abroad and gave me an expensive pair of pants.

69. “麻屋子，红帐子，里面住着个白胖子”这个谜语描述的是（花生 hua sheng）。 （90.63%）

The riddle "A hemp house with a red tent and a fat white man living inside" describes peanut.

河南是个农作物大省，种植了很多可以用来榨油的农作物，比如（花生 hua sheng）。 （14.71%）

Henan is a major agricultural province and has grown many crops that can be used for oil extraction, such as peanuts.

70. 在地球的南极地区生活着很多胖乎乎的、呆萌可爱的（企鹅 qi e）。（94.12%）

In the Antarctic region of the earth, there are many chubby, cute and cute penguins).

她穿着厚厚的衣服裹得严严实实的，看起来就像一只（企鹅 qi e）。（16.00%）

She was wrapped up in thick clothes and looked like a penguin.

71. 在这次音乐会上，我终于亲眼见到了朗朗在弹（钢琴 gang qin）。（100.00%）

At this concert, I finally saw Lang Lang playing piano.

空荡荡的房间里只剩下一台落满灰尘的(钢琴 gang qin) 。（4.00%）

In the empty room there is only a dusty piano.

72. 他每天吃吃睡睡，什么都不干，简直就像一头（猪 zhu）。（97.06%）

He eats and sleeps every day and does nothing. He is just like a pig.

让我感到意外的是，他居然在自己的别墅里养了一头（猪 zhu）。（9.09%）

What surprised me was that in his villa he actually raised a pig.

73. 他肯定不是一个人住，因为他的床上放了两个（枕头 zhen tou）。（96.88%）

He must not live alone because on his bed there are two pillows.

我不喜欢在奶奶的房间睡，因为她的床上没有（枕头 zhen tou ）。（11.76%）

I don't like sleeping in grandma's room because on her bed there is no pillow.

74. 商场里有两名小偷，请赶快拨打110求助(警察 jing cha) 。（88.00%）

There are two thieves in the mall. Please call 110 for help from police.

妈妈告诉我如果被坏孩子欺负了，就立即告诉(警察 jing cha) 。（4.55%）

My mother told me that if I am bullied by a bad boy, I should tell the police.

75. 万圣节到了，为了雕刻南瓜灯笼，我买了一个又大又圆的（南瓜 nan gua）。	（95.45%）

Halloween is here. In order to carve a pumpkin lantern, I bought a big, round pumpkin.

家里没有蔬菜了，我和奶奶准备去菜园里摘几个（南瓜 nan gua）。（0%）

There are no vegetables at home. My grandma and I are going to the vegetable garden to pick some pumpkins.

76. 去埃及旅游，我们最想看的就是世界八大奇迹之一的（金字塔 jin zi ta）。（88.24%）

When traveling to Egypt, what we most want to see is one of the eight wonders of the world: the Pyramids.

这个小男孩在沙滩上堆了一个沙堆，看起来就像一座（金字塔 jin zi ta）。（6.25%）

The little boy made a pile of sand on the beach that looked like a pyramid.

77. 乌龟虽然跑得很慢，但是因为坚持努力，最终跑赢了(兔子 tu zi ) 。（92.00%）

Although the tortoise ran very slowly, because of his persistence he finally defeated the rabbit.

今天和妹妹去森林公园玩，发现在草地里有一只(兔子 tu zi) 。（12.20%）

I went to the forest park to play with my sister today and in the grass found a rabbit.

78. 爸妈说饭菜吃不完也不用倒掉，可以把它们放进（冰箱 bing xiang）。（97.06%）

Parents say you don’t have to throw away the food if you can’t finish it. You can put it in the refrigerator.

去年过年的时候，我用自己的工资给爸妈买了一台（冰箱 bing xiang）。（0.00%）

During the Chinese New Year last year, I used my salary to buy my parents a refrigerator.

79. 有一句古诗是“不知细叶谁裁出，二月春风似(剪刀 jian dao)” 。（100%）

There is an ancient poem "I don't know who will cut out the thin leaves, like the spring breeze in February scissors".

他今天打开家里的工具箱找东西时，发现丢了一把(剪刀 jian dao) 。（24.00%）

When he opened the toolbox at home today to find something, he found that he had lost a pair of scissors.

80. 和其他爱美的女生一样，小丽喜欢夏天穿（裙子qun zi）。（90.63%）

Like other girls who love beauty, in summer Xiaoli likes to wear skirts.

今天和姐姐一起逛街，她给我买了一件（裙子qun zi）。（0.00%）

I went shopping with my sister today and she bought me a skirt.

81. 我要努力一步一步往上爬，就像背着一只重重的外壳的（蜗牛wo niu）。 (90.91%)

I will work hard to climb up step by step, like a snail.

一场大雨过后环境变得潮湿，路面上出现了很多只（蜗牛wo niu）。 (9.38%)

The environment became wet after a heavy rain, and on the road there are many snails.

82. 小时候我读过伊索寓言中一个故事，名字叫农夫与（蛇 she）。（100%）

When I was a child, I read a story from Aesop’s fables called The Farmer and the Snake.

我不敢一个人走这条路，因为这里有很多(蛇 she) 。（4.00%）

I dare not walk this road alone because there are many snakes.

83. 下雪天我们最喜欢的事情就是在雪地里堆（雪人 xue ren）。（94.12%）

Our favorite thing on snowy days is to build snowmen.

午夜十二点，小女孩蹑手蹑脚地起身去看(雪人 xue ren) 。（0%）

At twelve o'clock at midnight, the little girl got up quietly to watch Snowman.

84. 像贝克汉姆和C罗一样，我的两个弟弟也喜欢踢（足球 zu qiu）。（96.88%）

Like Beckham and Cristiano Ronaldo, my two younger brothers also like to play football.

个子矮小的她穿着一个不合身的外套，上面印着一个 (足球 zu qiu) 。（0%）

Short of stature, she wore an ill-fitting jacket emblazoned with a football.

85. 为了学地理，妈妈给我买了一个和地球真实形状一样的（地球仪 di qiu yi）。 （97.06%）

In order to learn geography, my mother bought me a globe.

这个小男孩太厉害了，居然用乒乓球做了一个小小的(地球仪 di qiu yi) 。 （13.64%）

This little boy is so awesome, as he used a table tennis ball to make a small globe.

86. 我的爸爸从年轻时候就喜欢收集信封上粘贴的（邮票 you piao）。（100%）

Since he was young, my dad has been collecting stamps.

让我感到诧异的是，姐姐的房间里到处都贴满了（邮票 you piao）。（0.00%）

What surprised me was that my sister’s room was covered with stamps.

87. 女朋友怕我上半身冷，就买毛线给我织了一件（毛衣 mao yi）。（84.85%）

My girlfriend was afraid that my upper body would get cold, so she bought wool and knitted me a sweater.

天气预报说今天要降温，妈妈让我多穿一件（毛衣 mao yi）。（0%）

The weather forecast says it will be cooler today, so my mother asked me to wear an extra sweater.

88. 这个狡猾商人终于按捺不住了，露出了狐狸（尾巴 wei ba）。（88.24%）

The cunning businessman finally couldn’t hold himself back anymore and revealed his fox tail.

姥姥给我买了一只小白兔，我特别喜欢它的（尾巴 wei ba）。（0.00%）

Grandma bought me a little white rabbit and I especially like its tail.

89. 校长办公室的桌子上有一台电脑和一部固定(电话 dian hua ) 。（92.00%）

On the desk in the principal’s office, there is only one computer and a telephone.

这个乡村学校设施很落后，全校上下只有一部(电话 dian hua) 。（11.76%）

The facilities of this rural school are very backward, and in the whole school there is only one (telephone).

90. 今天我们要在野外露宿，所以需要带一个（帐篷 zhang peng）。（91.12%）

Today we are going to sleep in the wild, so we need to bring a tent.

这种布料耐磨又防水，所以适合用来制作（帐篷 zhang peng）。（0.00%）

This kind of fabric is wear-resistant and waterproof, so it is suitable for making tents.

91. 如果你想精确地测量室内的温度，就需要一支（温度计 wen du ji）。（95.45%）

If you want to accurately measure the indoor temperature, you need a thermometer.

为了严格记录化学实验发生的条件，实验人员需要用到（温度计 wen du ji）。（2.94%）

In order to strictly record the conditions under which chemical experiments occur, experimenters need to use thermometers.

92. 在重要的场合，男人应该穿西装打（领带 ling dai）。（94.12%）

On important occasions, men should wear a suit and tie.

儿子已经二十岁了，但是还是不会系(领带 ling dai) 。（3.13%）

My son is twenty years old, but he still can’t tie a tie.

93. 早上的时候我没有刷牙，因为我找不到自己的（牙刷 ya shua）。（100.00%）

I didn’t brush my teeth in the morning because I couldn’t find my toothbrush.

爸爸每次出差住酒店回来，都会带回来很多（牙刷 ya shua）。（9.09%）

Every time my father comes back from a business trip and stays in a hotel, he will bring back a lot of toothbrushes.

94. 不要在这条铁路上玩耍，因为这里即将驶来一列（火车 huo che）。（93.75%）

Don’t play on this railway line because there will be a train.

你能不能快一点？再慢的话我们就赶不上这趟 (火车 huo che) 。（11.76%）

Can you hurry up? If we go any slower, we won't be able to catch this train.

95. 现在人们洗衣服不需要手洗了，因为家里有（洗衣机 xi yi ji）。（94.18%）

Nowadays, people no longer need to wash clothes by hand because they have a washing machine.

奶奶家里的电器少得可怜，只有一台破旧的(洗衣机 xi yi ji) 。（0.00%）

There are very few electrical appliances in grandma's house, only a worn-out washing machine.

96. 为了彰显气质，男人们都喜欢在手腕上佩戴一块（手表 shou biao）。（96.88%）

In order to show their temperament, on their wrist men like to wear a watch.

这个女生是个富二代，家里面有很多国际名牌的（手表 shou biao）。（0.00%）

This girl is a rich second generation, and her family owns many international brands watches.

**Appendix C**

**Stimuli for Experiment 3**

All 84 pairs of sentence contexts from Experiment 3 are listed below with the first one in each pair being more predictable and the second one being less predictable. The mean cloze value of the target word in each pair is shown at the end of each sentence.

1. 今天是母亲节，我要去花店给母亲买（花hua）。（81%）

Today is Mother's Day. I am going to the flower shop to buy my mom flowers.

这幅画的背景真美，里面有许多漂亮的（花hua）。（50%）

The background of this painting is so beautiful, because in the painting there are many beautiful flowers.

2. 今天的天空特别蓝，上边还飘着几朵白（云yun）。（95%）

The sky is particularly blue today, with a few white clouds.

小亮抬起头，看见天空中有很多的（云yun）。（61.9%）

Xiaoliang raised his head towards the sky and saw a lot of clouds.

3. 在大街上要注意文明，不要随地吐（痰tan）。（92%）

Be civilized on the street and don't spit.

我进病房时，正好看到一个病人在吐（痰tan）。（38.1%）

When I entered the ward, I happened to see a patient spitting.

4. 夏天到了，女孩子们都喜欢穿上漂亮的裙子去逛（街jie）。（89%）

Summer is here, and girls like to wear beautiful skirts and go shopping in the street.

妈妈每天下班后，都会开车经过这个热闹的（街jie）。（65%）

Every day after get off work, my mother drives by this busy street.

5. 吃鱼的时候要特别小心，因为里面有很多（刺ci）。（86%）

Be especially careful when eating fish because there are many thorns.

经过医生检查才发现，这个婴儿的眼睛里扎进去一根（刺ci）。（42.86%）

After a doctor's examination, it was discovered that the baby's eye had a thorn.

6. 因为我有糖尿病，所以我很少吃 (糖tang) 。（83%）

Because I have diabetes, I rarely eat sugar.

和其他小孩子一样，我弟弟也喜欢吃(糖tang) 。 （55%）

Like any other kid my brother likes sugar.

7. 不是我不想买这件衣服，而是因为口袋里没有（钱qian）。（95%）

It's not that I don't want to buy this dress, but because I don't have money.

在卫生间的抽屉里，我找到了前几天丢失了的 (钱qian）。（38.10%）

In the bathroom drawer, I found the lost money.

8. 杰克写给安娜的情书很真诚，字里行间都充满了对她的（爱ai）。（84%）

Jack's love letter to Anna is very sincere, and the lines are full of love.

无论男女，每个人都渴望拥有很多的（爱ai）。（61.90%）

Whether male or female, everyone desires to have a lot of love.

9. 你要多吃饭多运动，不然会很容易生（病bing）。（89%）

You have to eat more and exercise more, otherwise you will easily get sick.

小明今天给老师请假了没有来上课，因为他有(病bing）。（38.1%）

Xiao Ming asked the teacher for leave today and did not come to class because he was sick.

10. 李明是个素食主义者，他从来不吃（肉rou）。（92% ）

Li Ming is a vegetarian, he never eats meat.

这个学生每顿饭都会吃很多的（肉rou）。（40%）

At every meal, this student eats a lot of meat.

11. 小王打完篮球回来出了一身汗，所以第一件事就是去洗（澡zao）。（92%）

Xiao Wang was sweating after playing basketball, so the first thing he did was to take a bath.

晚上老公刚从外面回到家，他的老婆就开始催促他去洗（澡zao）。（52.38%）

As soon as my husband came home from outside at night, his wife started urging him to take a bath.

12. 艾米莉今年拿到了驾照，打算明年给自己买一台(车che)。（92%）

Emily got her driver's license this year and next year plans to buy herself a car.

王刚今年运气特别好赚了不少钱，所以打算明年买(车che) 。 （60%）

Wang Gang is very lucky this year and made a lot of money, so he plans to buy a car.

13. 夏天天气变化非常快，我们出门最好带一把(伞san)。（97%）

The weather changes very quickly in summer, so when we go out we'd better bring an umbrella.

无论春夏秋冬，我每次出门都会随身携带(伞san) 。（47.62%）

Regardless of spring, summer, autumn and winter, when I go outside I always carry an umbrella.

14. 今天是中秋节，大家吃完饭后一起坐在院子里赏(月yue)。（95%）

Today is the Mid-Autumn Festival. After eating, everyone sits in the yard to see the moon.

小时候我最喜欢的一件事就是和奶奶一起赏(月yue) 。（45%）

One of my favorite things as a kid was watching the moon.

15. 我姥姥生病了，我要去医院给她买(药yao)。（95%）

My grandma is sick, and I'm going to the hospital to buy her medicine.

我今天因为工作太忙就忘了给奶奶买(药yao) 。（52.38%）

Because I was too busy at work today, I forgot to buy medicine for grandma.

16. 妈妈早上刚拖完地，进门请换(鞋xie)。（92%）

Mom just finished mopping the floor in the morning, so when entering the door please change your shoes.

妈妈答应我，如果我这次考满分，就给我买新(鞋xie) 。(52.38%)

My mother promised me that if I get a perfect score in this test, she will buy me new shoes.

17. 以前通信技术不发达，人们之间联系主要靠写(信xin)。(89%)

A long time ago, the communication technology was underdeveloped, and the communication between people mainly depended on writing letters.

妈妈起床后发现了儿子昨天给她留在桌子上的(信xin) 。 (47.62%)

Mom woke up and found the letter her son left on the table.

18. 铃声响了，老师走进教室给同学们上(课ke)。(89%)

The bell rang, and the teacher walked into the classroom to teach lesson.

我明天不用早起，因为我明天上午不需要去上(课ke) 。（50%）

I don't have to get up early tomorrow because I don't need to go to class.

19. 早上吃的饭太咸了，现在我特别渴,想喝(水shui)。（92%）

The meal I ate in the morning was too salty. Now I am very thirsty and want to drink water.

晚饭的时候他几乎什么都没吃，只喝了一些 (水shui)

At dinner he hardly ate anything but drank some water. （35%）

20. 植树节到了，学校组织我们去山上种(树shu) 。 （92%）

Arbor Day is here, and the school organizes us to plant trees.

我看到公园门口的街道旁边种了很多(树shu) 。（55%）

Beside the street at the entrance of the park I saw a lot of trees.

21. 小红是个懂事的孩子，吃完饭总是抢着帮妈妈洗(碗wan)。（92% ）

Xiaohong is a sensible child, always rushing to help her mother wash dishes.

家里的猫咪跳到客厅的桌子上，打碎了一只(碗wan) 。（52.38%）

The family cat jumped on the living room table and broke a bowl.

22. 家里的窗户被打碎了，门锁也被撬了，肯定进了(贼zei)。（84%）

The windows at home were broken and the door lock was picked, so there must be a thief.

我放学回到家的时候，正好看到家里进了一个(贼zei) 。 （47.62%）

When I came home from school, I happened to see a thief.

23. 他实在太胖了，真的需要赶紧减(肥fei)。（92%）

He is too fat and really needs to lose weight.

我不想吃这块肉了，因为它太(肥fei) 。（52.38%）

I don’t want to eat this piece of meat because it’s too fat.

24. 为了锻炼身体，我每天吃完晚饭后都去操场跑(步bu)。（92%）

In order to exercise, every day on the playground after dinner I go for a run.

我也不知道她去哪里了，她只说要去一个人少的地方去散(步bu) 。（60%）

I don’t know where she went, she just said she was going for a walk.

25. 最近天气太热了，真期待能下一场大(雨yu)。（92%）

The weather has been too hot recently. I really look forward to a heavy rain.

最近天气太冷了，天气预报说将会有一场大(雨yu) 。（55%）

The weather has been too cold recently. The weather forecast says there will be heavy rain.

26. 春节快要到了，我期待学校赶紧放寒(假jia)。（95%）

The Spring Festival is coming soon, and I am looking forward to the winter vacation.

小刚接下来两星期都不用去上班，因为他要休(假jia) 。（55%）

Xiaogang doesn’t have to go to work for the next two weeks because he has to take a vacation.

27. 王刚考了班级倒数第一名，他现在很伤(心xin)。（92%）

Wang just took the exam and ranked last in the class. He is very sad now.

无论我怎么对他，他都不相信我是真(心xin) 。（52.38%）

No matter how I treat him, he doesn’t believe that I am sincere.

28. 爷爷退休之后很清闲，每天都去河边钓(鱼yu)。（92%）

Grandpa has a lot of leisure time after retirement. He goes to the river to going fishing.

夏天的时候，他最喜欢的事情就是去捉(鱼yu) 。（57.14%）

In summer, his favorite thing is to catch fish.

29. 今天是开学第一天，老师会给我们发新(书shu)。（86%）

Today is the first day of school, the teacher will send us a new book.

对于爷爷来说，他的唯一爱好就是看(书shu) 。（50%）

For Grandpa, his only hobby is reading books.

30. 爸爸给我买的这双鞋太小了，穿起来磨(脚jiao)。（95% ）

The pair of shoes my father bought for me are too small, and they wear out their feet.

他最近一个月都不能下床走路，因为他在篮球赛中伤到了(脚jiao) 。（52.38%）

He couldn't get out of bed and walk for the last month because in a basketball game he hurt his feet.

31. 我想把这个西瓜切开，请去厨房给我拿一把(刀dao)。（95%）

I want to cut this watermelon, please go to the kitchen and get me a knife.

路人趁这个醉汉不注意，一把抢过了他手里挥舞着的(刀dao) 。（47.62%）

A passer-by took advantage of the drunk man's inattention and snatched the knife.

32. 我们要节约用电，白天光线充足的时候不要开(灯deng)。（89%）

We must save electricity, and when there is sufficient light during the day, please do not turn on (lights)

他再次提醒我最后离开房间的时候要记得关(灯deng) 。（60%）

When I finally leave the room he reminded me again to turn off the lights.

33. 我们要讲文明守秩序，在食堂打饭的时候不要插(队dui)。（92%）

We must talk about civility and order, and when eating in the cafeteria don't intervene queuing.

小亮从教室窗户里看到三年级的同学正在操场上排(队dui) 。（61.9%）

From the window of the classroom, Xiao Liang saw on the playground the third grade students queuing.

34. 他刚做过手术，医生嘱咐他不要抽烟喝(酒jiu)。（92%）

He just had an operation, and the doctor told him not to smoke or drink wine.

舅舅来我家拜年时，给我们送了一箱(酒jiu) 。（60%）

When my uncle came to my house to pay New Year's greetings, he gave us a case of wine.

35. 兰兰和她的男朋友已经相恋8年了，打算明年结(婚hun)。（92%）

Lanlan and her boyfriend have been in love for 8 years and next year plan to get married.

这个男孩和女孩正在商量该如何处理这段短暂的感情，他们不知道是否应当结(婚hun) 。57.14%

The boy and the girl are discussing how to deal with this short-term relationship, and they don't know whether they should get married.

36. 结婚后夫妻双方要对彼此忠诚，千万不要出(轨gui)。（92%）

After marriage, the husband and wife should be loyal to each other and never cheat on each other.

晚上吃饭的时候，他给自己妻子说他不会出(轨gui) 。（47.62%）

When having dinner at night, he told his wife that he would not cheat.

37. 这个世界上没有无缘无故的爱，也没有无缘无故的(恨hen) 。 （89% ）

There is no love without reason in this world, and likewise there is no hatred.

这个男人的一番话让在场的所有人都对他充满了(恨hen) 。（38.1%）

This man’s words made everyone present hate.

38. 我刚下飞机，就看到姑姑在不远处向我招(手shou) 。 （95% ）

As soon as I got off the plane, I saw my aunt waving hands.

我很羡慕我的姐姐，因为她有一双纤细的(手shou) 。（65%）

I envy my sister because she has a pair of slender hands.

39. 为了保持口腔健康，最好每天刷两次(牙ya) 。 （92%）

In order to maintain oral health, it is best to brush your teeth.

在医院里，医生正在用一个小镜子查看病人的(牙ya) 。（61.9%）

In the hospital, the doctor is using a small mirror to look at the patient's teeth.

40. 站在一望无际的大草原上，我想去骑(马ma) 。 （89%）

Standing on the endless prairie, I want to ride a horse.

这个身材高大体格健壮的蒙古男人手里牵着(马ma) 。（61.9%）

This tall and strong Mongolian man is holding a horse.

41. 他不能参加比赛了，因为他的腰部受了重(伤shang) 。 （92%）

He can't take part in the competition because he hurt his waist.

让人愤怒的是，这名怀着身孕的女子竟然被她老公打(伤shang) 。（38.10%）

What makes people angry is that this pregnant woman was injured by her husband.

42. 这位德高望重的科学家刚进会场，观众就立马起立鼓(掌zhang) 。 （92%）

As soon as this respected scientist entered the venue, the audience immediately stood up and applauded.

无论今天儿子的表演水平到底怎么样，我们都要给他鼓(掌zhang) 。（42.86%）

No matter what the performance level of our son is today, we will applaud.

43. 最近小偷很多，你走的时候千万不要忘了锁(门men) 。 （92%）

There have been a lot of thieves recently, so when you leave don't forget to lock the door.

他慢慢抬起身子，用眼神示意旁边的护士去关(门men) 。（47.62%）

He slowly raised his body and signaled the nurse next to him to close the door with his eye.

44. 虽然我脸上的伤好了，但是却留下了一道(疤ba) 。（89%）

Although the wound on my face healed, there was a scar.

如果你仔细看的话会发现她的脸上有很深的(疤ba) 。（50%）

If you look closely, on her face there are deep scars.

45. 他是一名户外运动爱好者，每到周末都要去爬(山shan) 。 （92%）

He is an outdoor sports enthusiast, and every weekend he has to climb mountain.

我们都没有注意到，这里居然还有一座(山shan) 。（57.14%）

None of us noticed that there is actually a mountain.

46. 这个星期我要去美术馆，因为里面展览了很多幅达芬奇的(画hua) 。（95%）

I'm going to the art museum this week, because there are many Leonardo da Vinci's painting.

警察经过两周的调查发现，这名贪官的家里藏了很多名(画hua)。(60%)

After two weeks of investigation, the police found that in the corrupt official's home there were many famous paintings.

47. 雪莉是个性格内向的女孩，不喜欢和同学说(话hua) 。 (92%)

Shirley is an introverted girl who doesn't like talking.

我不是很了解这个女生，只知道她平时没有很多(话hua) 。(52.38%)

I don’t know this girl very well, I only know that she doesn’t talk.

48. 马上就要交卷了，我还有两道题没做出来，急得直挠(头tou) 。(84%)

I'm about to hand in the paper, and I still have two questions to answer, so I'm in such a hurry and scratching my head.

小亮今天爬山的时候不小心被一块落下来的石头砸伤了(头tou) 。(45%)

Xiaoliang was accidentally hit by a falling stone while hiking today and injured his head.

49. 赵丽颖是个十足的工作狂，忙起来总是顾不上吃(饭fan) 。 (89%)

Zhao Liying is a complete workaholic, and she always does not have time for food.

王亮告诉我说他待会儿要去公司给女朋友送(饭fan) 。(50%)

Wang Liang told me that he would go to the company later to deliver his girlfriend food.

50. 范冰冰是个劣迹艺人，因为她曾涉嫌偷(税shui) 。 (81%)

Fan Bingbing is a bad artist because she was once suspected of stealing taxes.

工作后爸爸妈妈一再叮嘱我，一定要按时交(税shui) 。 (55%)

After working, my parents repeatedly told me that I must pay tax.

51. 现在的很多明星和网红都在抖音上做直播带(货huo) 。(92%)

Many celebrities and Internet celebrities now make live broadcasts on Tiktok to sell goods.

因为疫情这个小区明天就要被封锁了，我们要赶紧去囤(货huo) 。(61.90%)

Because of the epidemic, this community will be blocked tomorrow, so we have to stock up goods.

52. 赶紧去楼顶把衣服收了，因为天要刮大(风feng) 。 (84%)

Go to the roof of the building quickly to collect your clothes, because there will be wind.

我特别讨厌这个地区的天气，因为经常有大(风feng) 。(50%)

I particularly hate the weather in this area because there is often wind.

53. 他的度量实在太小了，因为一件小事就跟我翻(脸lian) 。(81%)

His tolerance is too small, and he would fall out with me over a trivial matter.

昏暗的灯光下，小琴泪眼朦胧地看着他的(脸lian) 。(60%)

Under the dim light, with tearful eyes Xiaoqin looked at his face.

54. 如果你不按规则随便乱停车，就会被罚(款kuan) 。 (76%)

If you park randomly without following the rules, you will be fined.

刚刚收到一封邮件，看到发货方催促我们打(款kuan) 。(57.14%)

I just received an email and saw that the shipper urged us to pay.

55. 李白是一个很有才华的人，他的一生作了很多首(诗shi) 。 (92%)

Li Bai was a very talented man and in his life wrote many poems.

这个可爱的小男孩是我的外甥，我的姐姐正在教他背(诗shi) 。(50%)

This cute little boy is my nephew and my sister is teaching him to recite poetry.

56. 春天来了，花丛中有很多蜜蜂在勤劳地采(蜜mi) 。 (86%)

Spring is coming, and there are many bees in the flowers working hard to collect honey.

听到这话，他高兴地不得了，感觉就像吃了(蜜mi) 。(40%)

Hearing this, he was so happy that he felt like he had eaten honey.

57. 他是个数学天才，每次数学考试都能拿满(分fen) 。 (92%)

He is a math genius and on every math test he gets full marks.

小明今天在医院附近乱停车，所以被交警扣了(分fen) 。(52.38%)

Xiao Ming parked randomly near the hospital today, so the traffic police deducted his points.

58. 昨天我过生日，妈妈给我做了满满的一桌(菜cai) 。 (89%)

It was my birthday yesterday, and my mother cooked a table of dishes.

暑假在家吃饭时，妈妈不停地往我的碗里夹(菜cai) 。(50%)

When I was eating at home during the summer vacation, my mother kept adding vegetables.

59. 美国已经有3000万人感染了新冠病毒，今天又有两千人被确(诊zhen) 。 (95%)

Thirty million people in the United States have been infected with the new coronavirus, and today 2,000 more people have been diagnosed.

正在吃饭的医生撂下筷子就走，因为护士告诉他医院有急(诊zhen) 。(61.9%)

The doctor who was eating put down his chopsticks and left because the nurse told him that the hospital had an emergency.

60. 脚踩在雪地上发出咯吱咯吱的声音，身后留下一串清晰的脚(印yin) 。 (89%)

Our feet made a creaking sound on the snow, leaving a series of clear footprints.

期末考试需要准备很多资料，院长说可以去他的办公室复(印yin) 。(47.62%)

You need to prepare a lot of materials for the final exam. The dean said you can go to his office to make copies.

61. 给患者做手术前，医生都会先对伤口进行消(毒du) 。 (89%)

Before operating on a patient, the doctor will disinfect the wound.

我不敢吃陌生人提供的饭菜，因为我担心里面被人下(毒du) 。(52.38%)

I dare not eat food provided by strangers because I am worried that it will be poisoned.

62. 哥哥今年炒股赚了很多钱，所以又买了一套(房fang) 。 （89%）

Brother made a lot of money from stock trading this year, so he bought another house.

去年过年的时候，我在父母的资助下买了(房fang) 。（47.62%）

During the Chinese New Year last year, with the support of my parents I bought a house.

63. 你这篇作文文采不错，但是里面有很多错别(字zi) 。 （95% ）

Your essay is of good literary quality, but there are many mistaken words.

上完课，弟弟跑过来对我说他不认识这个(字zi) 。（65%）

After class, my younger brother came to me and said he didn’t know the word.

64. 哥哥抢走了妹妹的布娃娃，妹妹哭着去找妈妈告(状zhuang) 。（81%）

The brother took away the sister's doll. The sister cried and went to her mother to complain.

哥哥在军队立了大功，国家颁发给他一个奖(状zhuang) 。（40%）

My brother has made great contributions in the army, and the country gave him an award.

65. 最近公司很忙，老板逼着我们天天加(班ban) 。 （92%）

The company is very busy recently, and every day the boss forces us to work overtime.

张亮装病请了假，因为他今天太累不想去上(班ban) 。（52.38%）

Zhang Liang pretended to be sick and asked for leave because he was too tired today and didn’t want to go to work.

66. 今天上学你要多穿些衣服，因为天气要降(温wen) 。（84%）

You have to wear more clothes when you go to school today, because the weather is going to drop temperature.

天气预报说再过几天这个地区要降(温wen) 。（38.10%）

The weather forecast says that the temperature in this area will drop by.

67. 我的表姐已经30岁了还没有男朋友，舅舅让她明天去相(亲qin) 。 （95%）

My cousin is 30 years old and has no boyfriend yet. Tomorrow my uncle asked her to go on a blind date.

今天是重要的日子，早上五点他就起床准备去迎(亲qin) 。（50%）

Today is an important day, he wakes up at five o'clock in the morning and prepares to greet his relatives.

68. 由于通货膨胀的缘故，人民币在不断地贬(值zhi) 。（92%）

Due to inflation, the renminbi is constantly depreciating.

她花50元买了三个苹果，却发现它们又酸又小，所以觉得这些苹果不(值zhi) 。（47.62%）

She bought three apples for 50 yuan, but found that they were sour and small, so she felt that the apples were not worth the prize.

69. 他第一次一个人来到这个陌生的城市，晚上特别想爸妈想回(家jia) 。 （89%）

It was the first time he came to this strange city alone, and at night he especially missed his parents and wanted to go home.

晚上女儿独自一个人在学校，她难过地告诉好朋友说她想(家jia) 。（42.86%）

The daughter was alone at school at night, and she sadly told her best friend that she was homesick.

70. 他没有本事但是又死要面子，所以总在朋友面前吹(牛niu) 。 （86%）

He has no skills but wants to save face, so he always brags (niu) in front of his friends.

他非常地倔强固执，就像一头(牛niu) 。（60%）

He is very stubborn and stubborn, like a cow.

71. 这个好消息来得太突然，感觉就像做了一场(梦meng) 。（95%）

This good news came so suddenly, it felt like a dream.

不知道是不是最近烦心事太多，他总是频繁做(梦meng) 。（45%）

I don’t know if he has too many worries recently, but he always dreams.

72. 我最近心情很不好，连着好几天晚上都失(眠mian) 。 （95%）

I have been in a bad mood recently and have been unable to sleep for several days in a row.

他没有告诉别人他最近看起来精神不好是因为他失(眠mian) 。（42.86%）

He didn’t tell others that he seemed to be in a bad mood recently because he had insomnia.

73. 她嫁给了一个离过婚带孩子的男人，所以年纪轻轻就当了后(妈ma) 。 （86%）

She married a divorced man with children, so she became a mother at a young age.

看到身后这个五十岁左右的女人，这名男子惊讶地叫了一声(妈ma)。（60%）

Seeing the fifty-year-old woman behind him, the man called out "Mom" in surprise.

74. 袁华是一名高三的学生，再有两个多月就要参加高(考kao) 。 （95%）

Yuan Hua is a senior high school student and will take the high school entrance exam in more than two months.

老师说要从小学和初中就打牢基础，才能更好地迎接高(考kao) 。（52.38%）

The teacher said that we need to lay a solid foundation from elementary school and junior high school in order to better prepare for the college entrance examination.

75. 为了降低新冠病毒传播风险，国家提倡我们要尽快打疫(苗miao) 。 （97%）

In order to reduce the risk of the spread of the new coronavirus, the state advocates that we should vaccinate (miao) as soon as possible.

在当下时刻，这个科研团队最主要的任务就是研究疫(苗miao) 。（52.38%）

At the moment, the main task of this scientific research team is to study vaccines

76. 明星吴越不喜欢化妆，她每次出席活动都是素(颜yan) 。 （95%）

Wu Yue doesn't like makeup, she always wears plain (Yan yan) every time she attends an event.

从这张照片中我们可以看出这个女明星的素(颜yan) 。（61.9%）

From this photo, we can see that the female star has no makeup.

77. 很多学生毕业之后没有找工作，而是选择了自主创(业ye) 。 （92%）

Many students did not find a job after graduation, but chose to start their own business.

截止日期马上就要到了，系里催促我们尽快提交自己的作(业ye) 。（50%）

Deadlines are approaching and the department is urging us to submit our own assignments.

78. 玲玲非常喜欢宠物，家里边养了很多猫和(狗gou) 。 （95%）

Lingling likes pets very much. There are many cats and gou in the house

和她妈妈一样，丽丽最喜欢的宠物也是(狗gou) 。（52.38%）

Like her mother, Lili's favorite pet is also a dog.

79. 作为一名职场上的打工人，我们最渴望的就是升职加(薪xin) 。（86%）

As a worker in the workplace, what we desire most is a promotion and a salary increase.

早会上公司的老板突然告诉王刚，说公司决定给他降(薪xin) 。（40%）

At the morning meeting, the boss of the company suddenly told Wang Gang that the company decided to cut his salary.

80. 山西煤矿发生瓦斯爆炸事故，8名伤者正在医院被抢(救jiu) 。 （86%）

A gas explosion accident occurred in a coal mine in Shanxi, and in the hospital eight injured people were rescued.

记者闻讯赶到医院时，两名歹徒正在医院进行抢(救jiu) 。（40%）

When the reporter heard the news and rushed to the hospital, two gangsters were being rescued in the hospital.

81. 他现在的妻子是二婚，并不是他的原(配pei) 。（84%）

His current wife is a second marriage, not his first wife.

队长和一群人正在讨论该如何对这些钱进行分(配pei) 。（52.38%）

The captain and a group of people are discussing how to distribute the money.

82. 现在医美技术越来越发达，很多想要变美的女生选择去整(容rong) 。（89%）

Nowadays, medical and aesthetic technology is more and more developed, and many girls who want to look beautiful choose to have plastic surgery.

现在的女孩子都特别爱美，她们一有空就会去做美(容rong) 。（45%）

Girls nowadays are very fond of beauty, and 45% of them will go for beauty treatments whenever they have time.

83. 每天放学后，王刚都要去操场找同学们踢足(球qiu) 。（95%）

Every day after school, Wang Gang goes to the playground to play football with his classmates.

儿子回家炫耀说，对方根本接不住他的(球qiu) 。（47.62%）

My son came home and showed off that the opponent couldn’t catch his ball.

84. 按照新的法律规定，男性需要工作到65岁才能退(休xiu) 。 （89%）

According to the new law, men need to work until they are 65 years old before they can retire.

刘伟给学校人事处打电话，说自己打算明年退(休xiu) 。（60%）

Liu Wei called the school personnel office and said that next year he planned to retire.
